# Identification of the early and late responder genes during the generation of induced pluripotent stem cells from mouse fibroblasts

**DOI:** 10.1371/journal.pone.0171300

**Published:** 2017-02-02

**Authors:** Jihwan Park, Yoo-Wook Kwon, Seokjin Ham, Chang-Pyo Hong, Seonghye Seo, Moon Kyung Choe, So-I Shin, Choon-Soo Lee, Hyo-Soo Kim, Tae-Young Roh

**Affiliations:** 1 Department of Life Sciences, Pohang University of Science and Technology (POSTECH), Pohang, Republic of Korea; 2 National Research Laboratory for Stem Cell Niche, Seoul National University College of Medicine, Seoul, Republic of Korea; 3 Innovative Research Institute for Cell Therapy and Cardiovascular Center & Department of Internal Medicine, Seoul National University Hospital, Seoul, Republic of Korea; 4 Department of Molecular Medicine and Biopharmaceutical Sciences, Graduate School of Convergence Science and Technology, and College of Medicine or College of Pharmacy, Seoul National University, Seoul, Republic of Korea; 5 Division of Integrative Biosciences and Biotechnology, Pohang University of Science and Technology (POSTECH), Pohang, Republic of Korea; University of Texas at Austin Dell Medical School, UNITED STATES

## Abstract

**Background:**

The generation of induced pluripotent stem cell (iPSC), a substitute for embryonic stem cell (ESC), requires the proper orchestration of a transcription program at the chromatin level. Our recent approach for the induction of pluripotent stem cells from fibroblasts using protein extracts from mouse ESCs could overcome the potential tumorigenicity risks associated with random retroviral integration. Here, we examine the epigenetic modifications and the transcriptome of two types of iPSC and of partially reprogrammed iPSCs (iPSCp) generated independently from adult cardiac and skin fibroblasts to assess any perturbations of the transcription program during reprogramming.

**Results:**

The comparative dissection of the transcription profiles and histone modification patterns at lysines 4 and 27 of histone H3 of the iPSC, iPSCp, ESC, and somatic cells revealed that the iPSC was almost completely comparable to the ESC, regardless of their origins, whereas the genes of the iPSCp were dysregulated to a larger extent. Regardless of the origins of the somatic cells, the fibroblasts induced using the ESC protein extracts appear to be completely reprogrammed into pluripotent cells, although they show unshared marginal differences in their gene expression programs, which may not affect the maintenance of stemness. A comparative investigation of the iPSCp generated by unwanted reprogramming showed that the two groups of genes on the pathway from somatic cells to iPSC might function as sequential reprogramming-competent early and late responders to the induction stimulus. Moreover, some of the divergent genes expressed only in the iPSCp were associated with many tumor-related pathways.

**Conclusions:**

Faithful transcriptional reprogramming should follow epigenetic alterations to generate induced pluripotent stem cells from somatic cells. This genome-wide comparison enabled us to define the early and late responder genes during the cell reprogramming process to iPSC. Our results indicate that the cellular responsiveness to external stimuli should be pre-determined and sequentially orchestrated through the tight modulation of the chromatin environment during cell reprogramming to prevent unexpected reprogramming.

## Introduction

iPSCs generated from somatic cells are attractive sources for the development of patient-specific regenerative medicines, as well as for drug discovery and toxicology testing in the near future. The first reprogramming of somatic cells into a pluripotent state was achieved using the ectopic expression of key transcription factors, such as Oct4, Sox2, c-Myc and Klf4 [1–3]. Great efforts have been made to improve the reprogramming efficiency and to reduce the potential risks arising from viral transduction. Various reprogramming protocols using non-integrating adenoviruses, plasmid transfection, recombinant proteins, and ESC-derived protein extracts have been introduced [4–7]. In addition, iPSC lines have been generated from multiple cell types, including hematopoietic progenitors, neural cells, pancreas, stomach and liver cells, fibroblasts, and keratinocytes [8–13].

The post translational modifications of histones, such as methylation, acetylation, phosphorylation, and ubiquitination, have been identified as the key regulatory mechanisms of the gene expression program. The high resolution genome-wide maps of diverse histone modifications have presented clear evidence of their involvement in many genomic functions and chromatin organization [14–18]. Among the many histone modifications studied to date, the roles of histone H3 tri-methylations at lysine 4 (H3K4me3) and lysine 27 (H3K27me3) are well-characterized regarding gene activation, repression, and a poised chromatin state [19–22]. All functional DNA elements marked by histone modifications have been extensively annotated by several international consortia, such as the Encyclopedia of DNA Elements (ENCODE), the NIH Roadmap Epigenomics Program, and the International Human Epigenome Consortium, *etc*., and by many individual groups, including ours [16, 23–28]. Moreover, the bivalent chromatin state characterized by the coexistence of two counteracting histone modifications, H3K4me3 and H3K27me3, represents the poised chromatin state of essential genes responsible for maintaining the stemness and differentiation potential of stem cells, as well as the cell type-specific genes of differentiated cells, such as T cells [15, 29–32]. A recent study also showed that epigenetic reprogramming should precede transcriptional re-activation and full reprogramming [33]. Various lines of research evidence indicate that epigenetic reprogramming is an essential process in the induction of pluripotent stem cells.

It has been shown that iPSCs globally recover ESC-like epigenetic states and ESC-like gene expression patterns [19, 34, 35]. However, a couple of studies indicated that the iPSCs have a certain degree of variance of their epigenomes and transcriptomes compared with ESCs [36, 37]. Some recent reports have proposed that the variances between the two cell types are laboratory-specific, implying that the discrepancies observed in the previous studies might be due to the experimental variation or the different cell culture condition [38–40]. In addition, the characterization of 20 human ESC lines and 12 iPSC lines revealed their global similarity and the existence of cell-line-specific outliers that may cause unwanted differentiation [41]. It has also been reported that partially reprogrammed cells that achieve a stable state between somatic cells and fully reprogrammed cells could be generated using the ectopic expression of defined factors [19, 42]. A proposed reprogramming mechanism is the step-wise transition from the differentiated to the pluripotent state, as follows: 1) induction of proliferation and down-regulation of fibroblast-specific transcription, 2) acquisition of epithelial cell character and the activation of some ESC markers, and 3) final activation of pluripotency-related genes [19, 43]. The immunofluorescence labelling study using partial iPSCs showed that a decrease in the number of heterochromatin foci precedes Nanog expression and euchromatin reorganization during reprogramming [44]. It is also shown that somatic cell-specific genes are more efficiently suppressed and that ESC-specific transcriptional regulators are poorly reprogrammed in partial iPSCs [42]

Previously, we have generated fully reprogrammed iPSCs from adult mouse skin and cardiac fibroblasts using a single treatment of mouse ESC-derived soluble proteins. Without genetic manipulation or foreign DNA, the somatic cells lost their character and became ESC-like cells, which were shown to be truly de-differentiated iPSCs using many different methods [7]. Interestingly, only cellular proteins extracted from C57BL/6 ESCs could be successfully reprogrammed into iPSCs, but not those from E14 ESCs from a genetic background of 129. The comparative proteomic analysis of iPSC and mESC showed that the reprogramming-competent C57BL/6-ESC expressed large amounts of proteins regulating protein synthesis and metabolism [45]. Meanwhile, the incomplete reprogramming of somatic cells gave rise to partial iPSCs (iPSCps) due to unexpected transcriptional control. To further assess the protein-based iPSCs, we obtained genome-wide profiles of histones H3K4me3 and H3K27me3 using ChIP-Seq; we also measured the gene expression levels of two types of iPSCs from cardiac and skin fibroblasts, mouse ESC and iPSCp from skin fibroblasts. Without a doubt, the chromatin states were dramatically changed during reprogramming into the pluripotent state, and the epigenetic modifications were accordingly associated with the changes of gene expression. The comparative analysis revealed that the iPSC acquired the pluripotent state epigenetically and transcriptionally. By focusing on the iPSCp, we could identify the reprogramming-competent responder genes, and we categorized them into the early and late responders responsible for explaining the step-wise reprogramming process. Our results could provide insights into the functional understanding of the epigenetic modifications involved in the differential responsiveness of genes during the induction of pluripotency.

## Materials and methods

### Cell culture and reprogramming

All protocols for stem cell culture and animal treatment were previously established [7]. The C57BL/6-background mouse ES cells (SCRC-1002, American Type Culture Cells (ATCC)) were cultured with a Mitomycin C-treated (Sigma-Aldrich, St. Louis, MO) STO feeder cell layer on 0.1% gelatin-coated tissue culture dishes (Sigma-Aldrich, St. Louis, MO). The adult cardiac fibroblasts (cFB) were prepared using the enzymatic digestion of the hearts harvested from 8-week-old C57BL/6 mice (The Jackson Laboratory, Bar Harbor, ME) and were incubated with anti-c-kit microbeads (Miltenyi Biotec, Auburn, CA). The c-kit-negative cardiac fibroblast cells were cultured for at least 4 passages with Dulbecco’s Modified Eagle’s Medium (DMEM) containing 10% fetal bovine serum (FBS) and antibiotics. The skin fibroblast, sFB was primarily cultured from the dermis of 8-week-old C57BL/6 and sFB-G from C57BL/6 mice harboring Oct4-promoter-GFP or Actin-promoter-GFP from the Jackson Laboratory, Bar Harbor, ME. Feeder cell-free ES cells and protein-iPS cells were harvested and used to prepare chromatin and isolate mRNAs. The C56BL/6 ES cells were permeabilized using streptolysin O (Sigma-Aldrich, St. Louis, MO), and the soluble proteins were extracted. After the fibroblasts were treated with the ES cell protein extract, the primary colonies were observed, reseeded onto an STO feeder layer, and subcultured. Passage 5 to 7 cells (culture days 45–55) were used to obtain enough number of cells preparing chromatin and RNA. The partial iPS cells, which show similar morphologies to the ES cell colonies at early passages but have different shapes after later passages, were collected. All animal experiments were performed after receiving approval from the Institutional Animal Care and Use Committee (IACUC) of the Clinical Research Institute of Seoul National University Hospital, Korea.

### Chromatin immunoprecipitation

Chromatin immunoprecipitation was performed according to our previous publications [16, 25]. Briefly, native mononucleosome-sized chromatin was prepared using Microccocal Nuclease digestion (Sigma, St. Louis, MO) in an ice-cold digestion buffer (50 mM Tris-HCI, pH 7.6, 1 mM CaCl_2_, 0.2% Triton X-100, 5 mM butyrate, 1 x proteinase inhibitor cocktail, and 0.5 mM PMSF). The chromatin was immunoprecipitated with antibodies specific to H3K4me3 (ab8580, Abcam, Cambridge, UK) and H3K27me3 (07–449, Millipore, Billerica, MA) in RIPA buffer (10 mM Tris, pH 7.4, 1 mM EDTA, 0.1% SDS, 0.1% Na-Deoxycholate, and 1% Triton X-100). The proteinase K-treated DNA was purified using phenol/chloroform extraction and dissolved in TE buffer. The enrichment of the purified DNA was validated using PCR.

### DNA library construction for high-throughput sequencing and ChIP-Seq analysis

The sequencing library was generated using the manufacturer’s instructions (Illumina, San Diego, CA), with some modifications [25]. After completing the sequencing, the image analysis, base calling, and alignment were performed using CASAVA 1.6 (Illumina, San Diego, CA). The sequence reads were mapped onto the mouse reference genome (NCBI37/mm9). The sequence data were deposited into the Sequence Read Archive (SRA) at the National Center for Biotechnical Information (NCBI) (GSE58965). To quantitate the enrichment of H3K4me3 and H3K27me3 in each gene, the tag density was calculated for the gene loci. The normalized tag densities of a 200-bp window were used to compare the distributions of the histone enrichment around the gene transcription start site (TSS) of all 22,584 RefSeq genes. To compare the ChIP-Seq data sets generated by different laboratories, we downloaded the histone modification ChIP-Seq data from the NCBI SRA database (GSE12241 and GSE15519).

### Gene expression analysis

The gene expression profiling was performed using Affymetrix GeneChip^®^ Mouse Gene 1.0 ST oligonucleotide arrays (Affymetrix, Santa Clara, CA) and Mouse Whole-genome BeadChips (Illumina, San Diego, CA). The sample was prepared according to the manufacturer’s instructions. After selecting valid probes, 15,608 annotated genes were used for the comparative analysis. To identify the differentially expressed genes, one-way ANOVA test was used with the following criteria: p-value < 0.01, and fold change ≥ 2. Public microarray data were downloaded from the NCBI GEO database (GSE13770 [7], GSE24930 [46], GSE17004 [47], GSE27814 [48], GSE22908 [49], GSE24046 [50], and GSE27087 [51], GSE10871 [19], GSE45352 [43]). Data sets from different labs were quantile-normalized for the comparison. After removing the control probes, the hierarchical clustering with Euclidean distance and complete linkage clustering was performed.

## Results

### Global chromatin signatures of somatic and pluripotent cells

**Fig 1:**
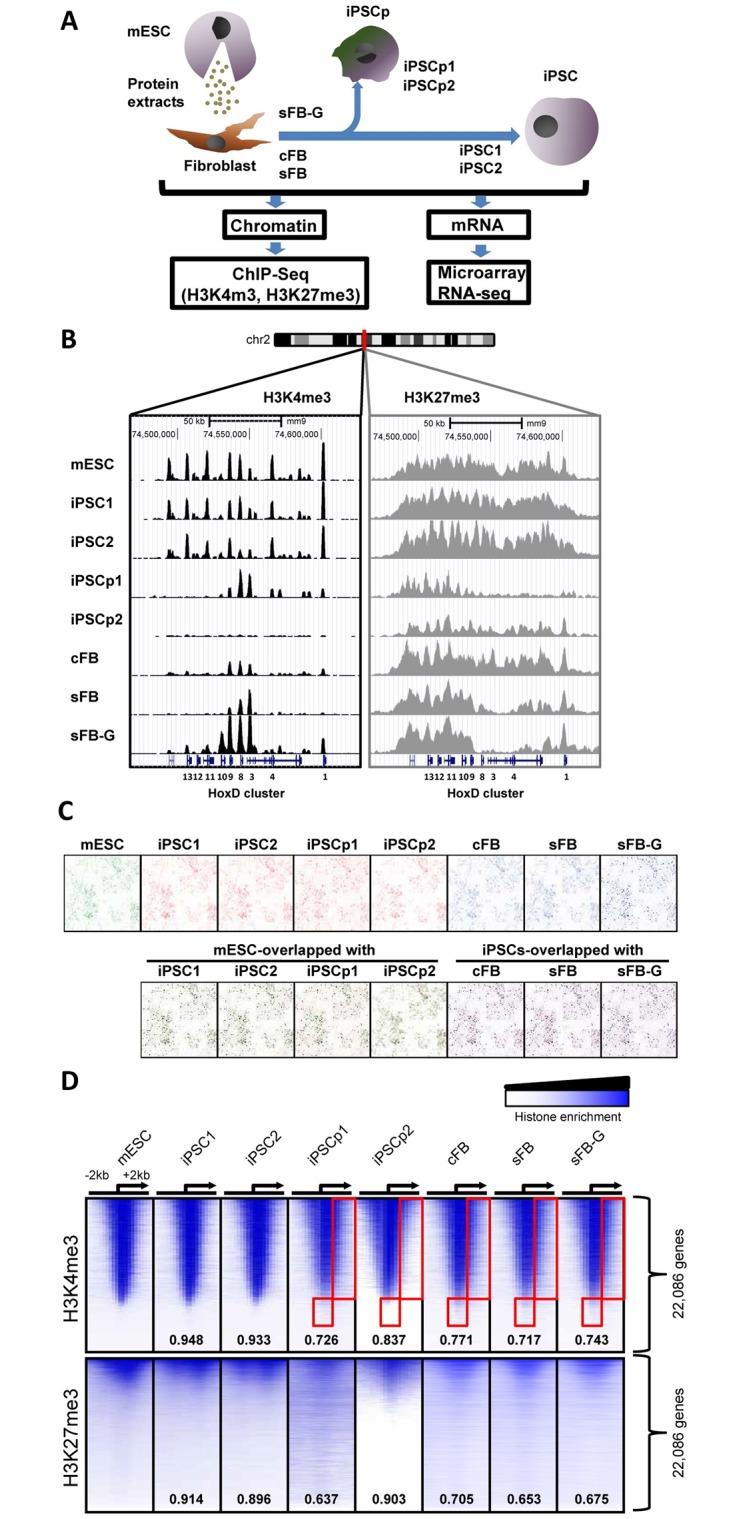
Global histone modification signatures of pluripotent and somatic cells. (A) Experimental overview of the genome-wide analysis of the histone modifications of mESC, iPSCs and the original somatic cells using ChIP-Seq and their gene expression measured via microarray and RNA-seq. (B) The histone modification profiles of the Hox D cluster genes are shown using the UCSC genome browser. The genomic position of the region is indicated on top of the map. (C) Chromosome-wide H3K4me3 peak patterns are compared in different cells. The Hilbert plots of H3K4me3 enrichments at chromosome 1 are visualized using the HilberVis program. (D) The comparison of histone modification patterns between different mESCs and iPSCs. The heatmap represents the distributions of the histone modification near the TSS of 22,086 RefSeq genes. The rows in the all data sets are sorted using the tag density of the mESCs from the highest to the lowest. The position of the TSS and the direction of transcription are denoted using an arrow. The numbers inside the heatmap indicate the Pearson correlation coefficients compared with the mESCs.

The primary fibroblast cells were isolated from mouse C57BL/6 strain and cell reprogramming was performed by treating with protein extracts from C57BL/6-ESC. The epigenetic and transcriptional statuses were examined using the isolated mono-nucleosomes and mRNAs from cFB and its iPSCs (iPSC1), sFB and its iPSCs (iPSC2), and sFB-G and its partial iPSC (iPSCp1 and iPSCp2); all reprogramming was performed using a one-time treatment of the somatic cells with the protein-extracts from mouse ESCs (Fig 1A). On the pathway to pluripotency, all genes might not change their epigenetic profile and expression patterns at the same time. To validate this hypothesis, two partially reprogrammed cells, iPSCp1 and iPSCp2, were obtained by selecting cell masses that had a different morphology from the mESCs at later passages. Using ChIP-Seq with histone H3K4me3 and H3K27me3 antibodies, the genomic enrichment of histone modifications was generally in accordance with the expected patterns of histone modifications. During cell reprogramming, the somatic cells assimilated into the epigenetic niche of ESCs. For the Hox D cluster genes, which are sequentially expressed and play an important role in controlling the developmental stages, their peak patterns in iPSC1 and iPSC2 bore a close resemblance to those of mESCs (Fig 1B). In cFB and sFB cells, the H3K4me3 enrichment was detected in the Hoxd3, d8, and d9 genes, but in iPSCs and mESCs, almost all Hox D cluster genes showed H3K4me3 enrichment. iPSCp1 still possessed some H3K4me3 peaks in Hox D cluster but iPSC2, not. In contrast to iPSCs, the H3K4me3 patterns of iPSCp1 were similar to those of the somatic cells, with some resemblance to somatic cells; for example, iPSCp1 had high levels of H3K4me3 in the 5’ region of Hoxd3. The H3K27me3 enrichment in the Hox D cluster was also reprogrammed during iPSC induction. A large amount of H3K27me3 level was changed in both iPSCP1 and iPSCP2. The chromosome-wide peak patterns of H3K4me3 were shown using Hilbert plots in Fig 1C (chromosome 1 only) and S1 Fig (all chromosomes). The patterns of the iPSCs almost completely overlapped with the patterns of the mESCs and were clearly different from those of their somatic origin cells, as expected. However, many peaks of mESC-iPSCp and iPSCp-sFB-G did not overlap with each other and many of them appeared only in iPSCp, but not in mESC or sFB-G, indicating that iPSCp1 and iPSCp2 were not completely reprogrammed into pluripotent cells and were even divergent from both mESC and sFB-G. The distributions of global histone modifications around TSSs were compared and were not apparently distinguishable from each other (Fig 1D). However, the differential enrichments of H3K4me3 in the gene body regions of somatic cells (red box area) seemed to be assimilated into the epigenetic modification patterns of mESCs by the induction of pluripotency, but those in iPSCp remained unchanged. The H3K27me3 profiles in cFB, sFB, and sFB-G showed much broader distributions and weaker enrichment correlations, particularly in the H3K27me3-rich genes in mESCs and iPSCs. The H3K27me3 levels in iPSCp were closer to those of somatic cells. In detail, the Pearson correlation coefficients of H3K4me3 enrichment between the two cell types generally showed a highly positive correlation (r > 0.8), suggesting that every genomic region was not totally different and that only a small fraction had an aberrant level of H3K4me3 (S2A Fig). The highest linkage of each pair was mESC-iPSC1 (r = 0.982) and the lowest one was iPSC1-sFB (r = 0.806). The overall distributions of H3K27me3 correlations were widely dispersed and the levels were low (S2B Fig). Their correlation coefficients were relatively smaller than those of H3K4me3, but the ranks were similar to those of H3K4me3; the highest coefficients were from pairs of mES and iPSCs, and the lowest coefficients were from pairs of mESC and somatic fibroblast cells.

### Gene expression program directed by histone modifications during cell reprogramming

**Fig 2:**
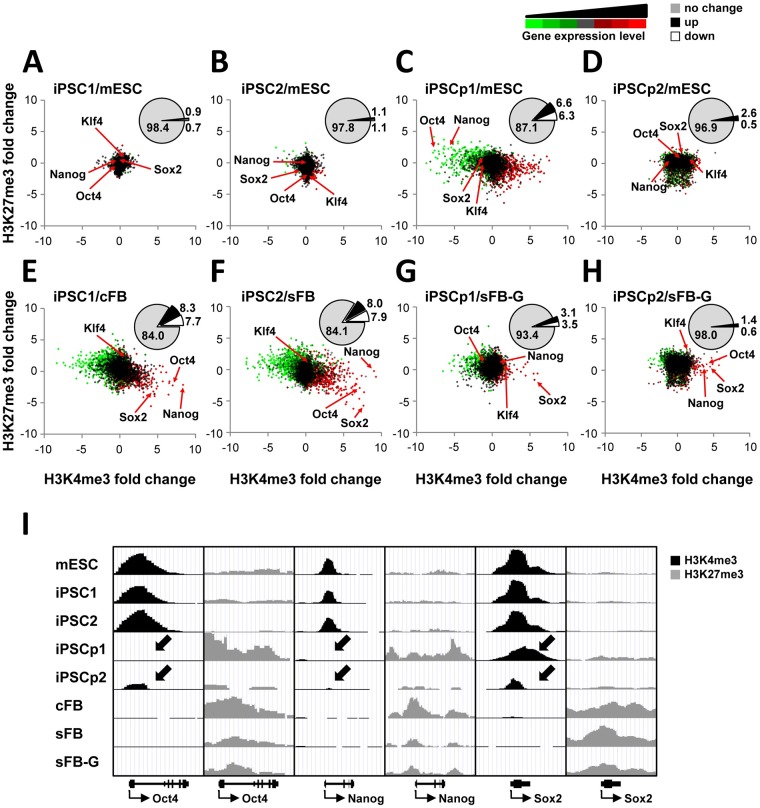
Characterization of epigenetic signatures related to gene expression in iPSCs. (A-F). The scatter plots show the fold changes in H3K4me3, H3K27me3, and gene expression during reprogramming. Each dot represents a gene with p-value less than 0.01. The X and Y axes indicate the fold changes in H3K4me3 and H3K27me3 (log2 scale), respectively. The color code shows the gene expression level from low to high (green-black-red). The percentage of differentially expressed genes is shown in the inset pie chart (ANOVA test p-value<0.01, fold change>2). (G) The chromatin states of the key transcription factor genes, such as the Oct4, Nanog, and Sox2, are shown on the UCSC genome browser.

As reported by many previous studies, histone modifications are tightly correlated with gene expression programs [14, 15]. To examine whether the dynamic changes in the gene expression program were accompanied by alterations in histone methylation during cell reprogramming, the gene expression and histone methylation levels were illustrated in the color-coded scatter plots (Fig 2A–2F). Most genes shown in black were converged at the center of each plot; thus, the histone modification and gene expression levels of iPSC1 and iPSC2 were almost the same as those of the mESCs (Fig 2A and 2B). The number of genes whose expression and histone modification levels were unchanged was approximately 98% in the comparison of iPSCs with mESCs, and approximately only 1% of the genes were either up-regulated or down-regulated (Fig 2A and 2B insets). In contrast, the two iPSCs were far away from their somatic cells, supporting the hypothesis that the reprogramming process completely transformed the cell types or lineages with respect to the gene expression program and epigenetic status (Fig 2E and 2F). The percentages of up- or down-regulated genes were increased to approximately 8% each; thus, 16.0% and 15.9% of the genes were epigenetically reconstituted and differentially expressed in iPSC1 and iPSC2, respectively (Fig 2E and 2F insets).

Many diverging genes were identified in the comparison between iPSCp and mESC, and the overall profile was completely different from those shown in Fig 2A or 2B. Both iPSCp1 and iPSCp2 seemed closer to their somatic cells regarding epigenetic behavior and transcription levels (Fig 2G and 2H). Only 87.1% of the iPSCp1 genes maintained similar expression levels to mESC, and 12.9% of genes were up- or down-regulated. Interestingly, iPSCp2 showed higher similarity to mESC (96.9%, Fig 2D). Compared to sFB-G, the iPSCp1 showed 93.4% similarity of gene expression and histone modification levels. This gene-wide comparison between iPSCp and mESC revealed that the histone modification and gene expression levels of many genes were not consistent with each other, raising the possibility of divergent cell reprogramming.

Focusing on the key transcription factors involved in stem cell maintenance, such as Nanog, Sox2, Oct4, and Klf4, there was little difference between iPSC and mESC in both histone modifications and expression levels (Fig 2I and S3 Fig). During reprogramming, the H3K4me3 levels and expression of these genes in iPSC1 were increased, whereas the H3K27me3 levels were decreased. It is worth noting that in iPSCp1, the transcriptional reprogramming of Oct4 and Nanog was not induced because they were still buried in the repressive chromatin region enriched with H3K27me3 (Fig 2G and S4 Fig). Interestingly, in iPSCp1, Sox2 was highly methylated at histone H3K4; accordingly, its expression level was also up-regulated. Constrast to iPSCp1, iPSCp2 showed partial histone modification chages and mRNA levels of key transcription factors almost reached the level of iPSCs, suggesting that iPCP2 might be at the later stage of reprogramming.

### Differential analysis of the genes aberrantly expressed in iPSCs

**Fig 3:**
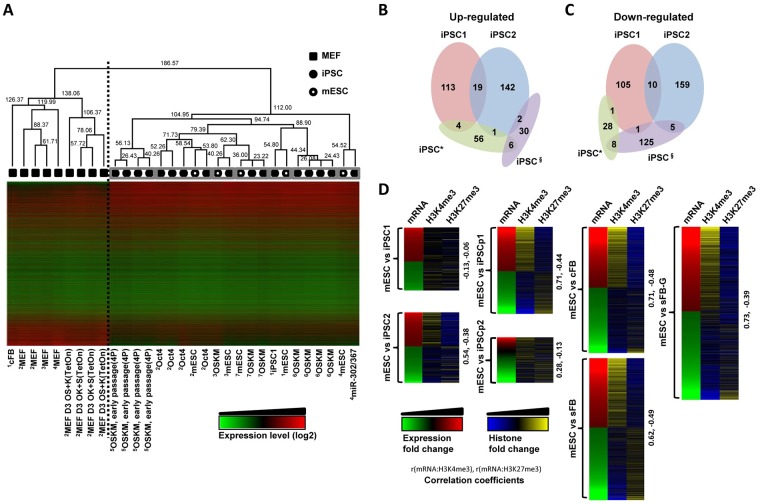
Comparative analysis of the protein-based iPSCs and other pluripotent cells. (A) Hierarchical clustering of the gene expression microarray data for the mESCs (open circles), iPSCs (filled circles), and fibroblasts (filled squares) from different laboratories. The data sets of the mESCs and iPSCs obtained from the same laboratory are marked using grey boxes. The iPSC induction methods are labelled for each iPSC data set: ^1^GSE13770, ^2^GSE24930, ^3^GSE17004, ^4^GSE27814, ^5^GSE22908, ^6^GSE24046, and ^7^GSE27087. (B-C) The Venn diagram shows the overlap of the differentially expressed genes between mESCs and iPSCs. For comparison, two iPSC* (GSE24046) and iPSC^§^ (GSE27814) data sets are also incorporated. (D) The gene expression levels and histone modifications of the differentially expressed genes defined in (B-C) are shown and the Pearson correlation coefficients are presented on the right side of each heatmap.

As shown in Figs 1 and 2, the protein-based iPSC and mESC were indistinguishable with respect to their epigenetic patterns and expression programs. To further assess the identity of iPSC after the cell reprogramming process, the gene expression profile of iPSC1 was compared with other publicly available iPSC data (Fig 3A). The relative similarity of the gene expression profiles among cell types was calculated using the hierarchical clustering analysis. The distance between iPSCs and somatic cells was the longest of the two large groups and was separated using a dotted line (node height = 186.57); pairs of iPSCs and mESCs cultured in the same laboratory were closely located (marked by grey boxes, node height < 75), indicating that the somatic cells and culture conditions of the iPSCs from different laboratories might lead to this minor divergence in the gene expression program, as considered in previous reports [38, 39]. Even the four transcription factor-driven (OSKM; Oct4, Sox2, Klf4, and c-Myc) iPSCs from two independent groups [50, 51] were positioned in different places on the hierarchical clustering of gene expression (node height = 94.74). Moreover, all mESCs were not clustered with each other, but were individually located next to each iPSC with which a research group maintains the mESCs. The gene expression levels of the iPSCs were broadly comparable. However, to examine how similar the iPSCs from different laboratories are or if the differences might be negligible, the differentially expressed genes (DEGs) of iPSCs relative to mESCs were compared; the common DEGs in iPSC1 and iPSC2 occupied only 6.9% (19/274) of the up-regulated genes and 3.6% (10/274) of the down-regulated genes (Fig 3B and 3C). Compared with the public data sets, there were no common DEGs among iPSC1, iPSC2, iPSC* [50], and iPSC^§^ [48]. A further examination of the histone modification levels of the DEGs revealed that the DEGs in iPSC2 but not in iPSC1 showed expression levels corresponding to the histone modification changes; the correlation was higher in iPSC2 than in iPSC1 (Fig 3D). In the comparison of somatic cells with mESCs, the fold changes in expression were well-correlated with the alterations in the histone modification levels. There was almost no correlation of the histone modification levels and the gene expression levels of mESCs vs. iPSC1, implying that the expression of these minor genes might not be affected by H3K4me3 and H3K27me3. A relatively good correlation of mRNA expression and histone modification levels was observed in iPSCps and in its somatic cell, sFB-G, where the expression levels of many genes reflect the degree of histone modifications. The differentially expressed genes are annotated in S4 Fig. The highly expressed genes of iPSCs were involved in RNA processing and splicing, mRNA/DNA metabolic process, ribonucleoprotein complex biogenesis, and chromosome organization. The fibroblast-specific genes were down-regulated and were associated with cell adhesion, vasculature development, blood vessel development, and extracellular matrix organization. These significant biological functions that were associated with the up- or down-regulated genes of iPSCs were also compatible with mESC-specific functions.

### Early and late responder genes identified from a comparative analysis of partial iPSCs and mESCs

**Fig 4:**
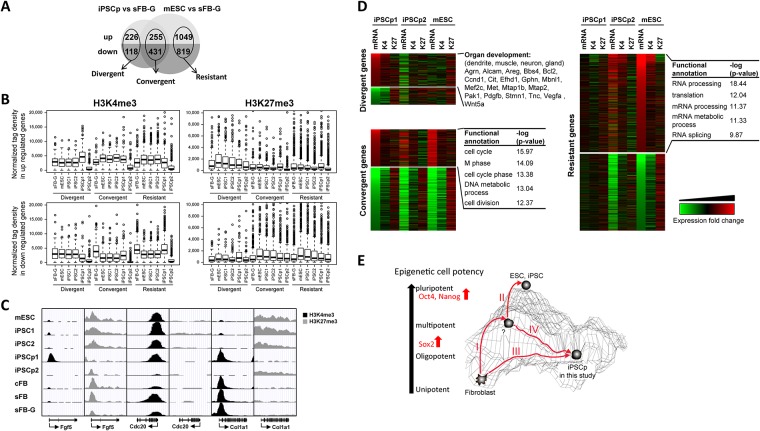
Identification of early and late responder genes from the partial iPSC analysis. (A) Categorization of the differentially expressed genes among iPSCps, mESCs, and sFB-G into three groups: the divergent, the convergent, and the resistant genes. (B) The H3K4me3 and H3K27me3 levels were plotted, depending on the responsiveness of the genes to the induction of pluripotency (C) The UCSC genome browser shows the chromatin states of the divergent (Fgf5), convergent (Cdc20), and resistant (Col1a1) genes in all cell types. The positions and directions of transcription of the genes are indicated below the panel. (D) The three groups are listed according to their expression and histone modification fold change values. The gene ontology analysis shows highly significant biological functions associated with the differentially expressed genes. (E) A model for the step-wise acquisition of pluripotency. Sox2 was identified as an early responder gene.

iPSC1 and iPSC2 were almost identical to mESCs at the transcriptional and epigenetic levels, with minor variations. The comparison of the histone modification and gene expression levels of the iPSCp1 with those of mESCs displayed a deep disparity. A further comparative analysis led to the categorization of differentially expressed genes into three groups: the divergent, the convergent, and the resistant genes (Fig 4A and S1–S6 Tables). Among the 2,554 differentially expressed genes in the mESCs compared to sFB-G (1,250 down-regulated and 1,304 up-regulated genes in the mESCs), 255 up-regulated genes were also increased in the iPSCp1, and 431 down-regulated genes were also decreased in the iPSCp1; these were called the “convergent” or the “early responder” genes before the start of pluripotency because the expression levels of these genes in iPSCp1 were comparable to the expression levels in the mESCs. The 226 up-regulated and 118 down-regulated genes in iPSCp1 vs. sFB-G were regarded as “divergent” genes because only iPSCp-specific regulated genes fell into this category. The third category was the “resistant” or the “late responder” genes, which were up- or down-regulated in mESC but not in iPSCp. The correlation coefficient was also calculated for the gene expression levels between mESC/sFB-G and iPSCp/sFB-G and was r = 0.546 for the down-regulated genes and r = 0.037 for the up-regulated genes, indicating that, in the partial iPSC, the expression of somatic cell-specific genes was efficiently repressed and that ESC-specific genes were activated during reprogramming.

As shown in Fig 4A, among the up-regulated genes in iPSCp1 and mESC relative to sFB-G, the average H3K4me3 level of the divergent genes was the highest in iPSCp1 and those of convergent and resistant genes were higher in pluripotent cells, such as mESC, iPSC1, and iPSC2, than in iPSCp1 and sFB-G (Fig 4B). The H3K27me3 level was positively correlated with the down-regulation of gene expression, such that among the down-regulated genes in iPSCp1 and mESC compared with sFB-G, the convergent and resistant gene groups had higher H3K27me3 levels in the pluripotent cells, but not in iPSCp1 and sFB-G. In iPSCp1, the divergent genes contained more H3K27me3 marks than the convergent or resistant genes.

As examples of these categories, both fibroblast growth factor 5 (Fgf5) and Sox2 were marked only by H3K27me3 in sFB-G. The Fgf5 gene belongs to the divergent gene group possessed high enrichment of H3K4me3 and a high level of expression in only iPSCp1, but not mESC (Fig 4C and S5 Fig). Sox2 was transcriptionally reprogrammed as de-differentiation was induced, and its H3K4me3 level was high in iPSCp; therefore, Sox2 could be classified as an early responder gene (Fig 2G). The epigenetic state and expression of the collagen type 1 alpha 1 (Col1a1) gene in iPSCp1 were not affected by pluripotency induction, and it was one of the resistant genes. Cell division cycle protein 20 (Cdc20) maintained a high level of H3K4me3 and expression before and after induction (Fig 4C and S5 Fig).

The biological functions of the divergent, convergent and resistant genes grouped using the propensity of fibroblast cells to obtain pluripotency revealed the following features (Fig 4D and S1–S7 Tables): many divergent genes were associated with organ development, such as dendrite, muscle, neuron, and gland, and many divergent genes were significantly involved in cytoskeleton and cancer-related pathways (S7 Table); these results imply that this iPSCp was far away from iPSCs and thereby partially differentiated. However, the convergent genes were connected with stem cell-related functions and could be regarded as early responder genes on the pathway from fibroblasts to iPSCs. From the gene ontology analysis of the convergent genes, the most significant biological functions were cell cycle, M phase, DNA metabolic process, and cell division (Fig 4D). The resistant genes, which were detected only in the mESCs but not in our iPSCps, might be late responder genes that are turned on or off at the last step of the reprogramming process. The resistant or late responder genes were linked to RNA processing, splicing, and metabolic process (Fig 4D). These three group genes were further compared with other published data (S6 Fig). The similar degree of differences between iPSCp1 and iPSCp2 was also detected in the comparison among iPSCp1, MCV6 and MCV8. The number of genes showing over 2-fold differences in gene expression was increased in resistant gene group during Dox-induced iPSC generation. Together, these results indicate that the iPSCps seem to have partial cellular potency and a cancer cell-like capability.

## Discussion

The aim of this study was to characterize the epigenetic status of protein-based iPSCs and to identify the responder genes during the induction of pluripotency by comparing iPSCs with partial iPSCs. We extracted soluble proteins from mouse ESCs on the C57BL/6 background and used them to induce pluripotency. These protein-based iPSCs were easy to generate, highly efficient, and their potential safety has already been validated [7]. The analysis of differential proteomes using Isobaric Tags for Relative and Absolute Quantification (iTRAQ) technique identified 1,883 proteins in iPSCs. Among them, 225 differentially expressed proteins were determined; they were mainly associated with the regulation of protein synthesis and metabolism, suggesting that there is a threshold that protein synthetic machinery must exceed to initiate reprogramming [45]. Here, we have mapped two major histone modifications involved in the determination of cell types and development, H3K4me3 and H3K27me3, of two types of iPSCs, partial iPSCs, and adult origin fibroblasts, as well as ESCs, which were used as a reference. The overall epigenetic profiles and gene expression data of iPSCs were almost indistinguishable from those of ESCs (Figs 1 and 2, S1 Fig). The H3K4me3 enrichment around TSSs was a bit broader in cFB and sFB than in iPSCs and ESCs, indicating that the active genes in the pluripotent cells are marked by high levels of active histone modifications at narrow regions and clearly separated from inactive genes (Fig 1D). These results are consistent with the tendency toward an overall increase in the active histone modification levels, such as H3K4me3, at the active loci in ESCs and iPSCs, but almost no changes in the cellular H3K27me3 levels between the somatic cells and pluripotent cells [44]. The hierarchical clustering of somatic and pluripotent cells indicates that the iPSCs were grouped with ESCs, depending on where the cells were maintained rather than the cell line origin (Fig 3A). There were no common genes detected that could account for the minor differences between our iPSCs and other groups’ iPSCs (Fig 3B and 3C). The comparison of the DNA methylation and gene expression levels revealed that there were more cell-line-specific outliers in iPSCs than in ESCs, despite their global similarity [41]. This handful of disparities may have important practical implications, such as the validation of a large number of iPSC lines and the continuous monitoring of cell line quality. However, as shown in Fig 3 and other groups’ reports [38, 39], the discrepancy seems to be largely dependent on the laboratory or specificity of the original cell line. The uncontrolled laboratory-specific variables and the cell passage-dependent changes caused by minutely different culture environments are also expected to contribute to the differential gene expression levels that were observed.

Interestingly, we observed distinct signatures that distinguish partial iPSCs from fully reprogrammed iPSCs. The Sox2 gene was reprogrammed prior to the expression of other pluripotency genes, such as Oct4 and Nanog, in the iPSCps. Other partial iPSCs are also known to fail to express many endogenous pluripotency genes, including Oct4 and Nanog [52]. Sox2 is known to be one of several genes that are highly expressed in pluripotent cell lines with relatively low variation [41] and are not regulated by DNA methylation, whereas the pluripotency regulators, such as Oct4 and Nanog, are repressed through DNA hypermethylation in somatic cells [19, 43]. Different from the conventional iPSC technology based on viral transduction of key transcription factors, where the initial induction of exogenous Oct4 and Nanog is usually observed at the early reprogramming stage, our protein-based iPSC approach might trigger the expression of endogenous Sox2 at early stage and sequentially followed by other factors (S3 Fig). There are three groups of differentially expressed genes identified using the comparison between iPSCp/sFG-G and mESC/sFB-G (Fig 4A). The divergent genes have different expression patterns from both somatic cells and mESCs and most of them are associated with functions in organ development. The KEGG (Kyoto Encyclopedia of Genes and Genomes) pathway analysis revealed that many divergent genes were associated with several cancer types (S7 Table). The expression levels of the convergent genes are perturbed at the early stage of reprogramming and eventually reach the range of the ES-specific gene expression program. The resistant genes in our iPSCps are not turned on or off but they should be regulated to become fully reprogrammed into the pluripotent state at the last step of the reprogramming process. The existence of divergent and resistant genes in the iPSCps might be an obstacle to complete cell reprogramming and may cause tumorigenicity when incomplete iPSCs are used in the clinic. We identified the tumorigenic potential of our iPSCps and showed that the *in vivo* use of the iPSCps in a mouse led to tumor formation (data not shown). Consistent with a previous report [42], the down-regulation of somatic cell-specific genes occurred more efficiently in the iPSCps than the up-regulation of ESC-specific genes, as deduced from the higher correlation value of mESC/sFB-G and iPSCp/sFB-G in somatic cell-specific genes (0.546) than in ESC-specific genes (0.037).

Based on these results, we suggest that the cellular reprogramming was accompanied by the step-wise alterations of the epigenetic status to reach a pluripotent status (Fig 4E). In this model, the iPSCs could be formed by pathway I and II in a step-wise manner. For some reason, the somatic cells could not overcome the threshold of pluripotency and remained at an oligopotent or multipotent stage in only pathway I. The iPSCps of this study did not have the middle stage between the somatic fibroblasts and the mESCs, and they also expressed some divergent gene groups. These iPSCps might be obtained using pathway III alone or a combination of pathways I and IV. The divergent gene group should determine the cellular fate of iPSCps regarding differentiation and tumorigenic potency.

When the cell reprogramming process is complete, the epigenetic signatures and gene expression programs are almost identical to those of ESCs. Our protein-based iPSC induction method using ESC soluble proteins can successfully establish iPSC lines that are almost indistinguishable from the ESCs epigenetically. The differences between protein-based iPSCs and ESCs may occur at similar or lower levels than other iPSC induction methods, but seemingly do not lead to significant functional differences. Importantly, the assessment of partial iPSCs provides more convincing evidence that the reprogramming process should undergo a step-wise transition that is tightly controlled by the early and the late responder genes in response to the induction signal during reprogramming. Taken together, our results indicate that cell fate determination should be accompanied by accurate epigenetic changes.

## Conclusions

The epigenetic characterization of protein-based iPSCs and partial iPSCs compared with fibroblasts and mESCs revealed several features. The iPSCs were almost epigenetically and transcriptionally identical to the mESCs, with negligible discrepancies. A comparative study of the partial iPSCs indicated that cellular reprogramming to generate iPSCs requires a step-wise epigenetic acquisition of pluripotency. The responsiveness of the cells to the induction of pluripotency should be determined by the presence of early (convergent) and late (resistant) responder genes in the pathway from fibroblasts to iPSCs. The potential tumorigenicity of iPSCs might be caused by incomplete reprogramming, during which the third group of divergent genes were dysregulated to a larger extent. In summary, the genome-wide comparison could suggest more convincing evidence that complete transcriptional reprogramming should be accompanied by faithful epigenetic changes in a step-wise manner and the cells’ responsiveness to external stimuli should be properly regulated to prevent unwanted reprogramming.

## Supporting information

S1 Fig Chromosome-wide ChIP-Seq profiles of iPSCs visualized by Hilbert curves. (PDF)

S2 Fig Pair-wise comparison between the cell types of H3K4me3 and H3K27me3. (PDF)

S3 Fig Gene expression fold changes of transcription factors. (PDF)

S4 Fig Functional annotation analysis using differentially expressed genes in iPSCs/fibroblasts and mESC/fibroblasts. (PDF)

S5 Fig Relative gene expression levels to somatic cells. (PDF)

S6 Fig Three group of genes and gene ontology analysis (A) and comparative expression analysis (B). (PDF)

S1 Table Gene expression fold changes and histone modification enrichment levels for the divergent genes (up) in iPSCp. (PDF)

S2 Table Gene expression fold changes and histone modification enrichment levels for the convergent genes (up) in iPSCp. (PDF)

S3 Table Gene expression fold changes and histone modification enrichment levels for the resistant genes (up) in iPSCp. (PDF)

S4 Table Gene expression fold changes and histone modification enrichment levels for the divergent genes (down) in iPSCp. (PDF)

S5 Table Gene expression fold changes and histone modification enrichment levels for the convergent genes (down) in iPSCp. (PDF)

S6 Table Gene expression fold changes and histone modification enrichment levels for the resistant genes (down) in iPSCp. (PDF)

S7 Table KEGG pathways for the divergent, convergent, and resistant genes in iPSCp. (PDF)
