# Supplementary material for: Identification of the early and late responder genes during the generation of induced pluripotent stem cells from mouse fibroblasts
Source: PLoS One. 2017 Feb 2;12(2):e0171300. doi: 10.1371/journal.pone.0171300 (PMC5289558; doi:10.1371/journal.pone.0171300)

S1 Fig

A

H3K4me3

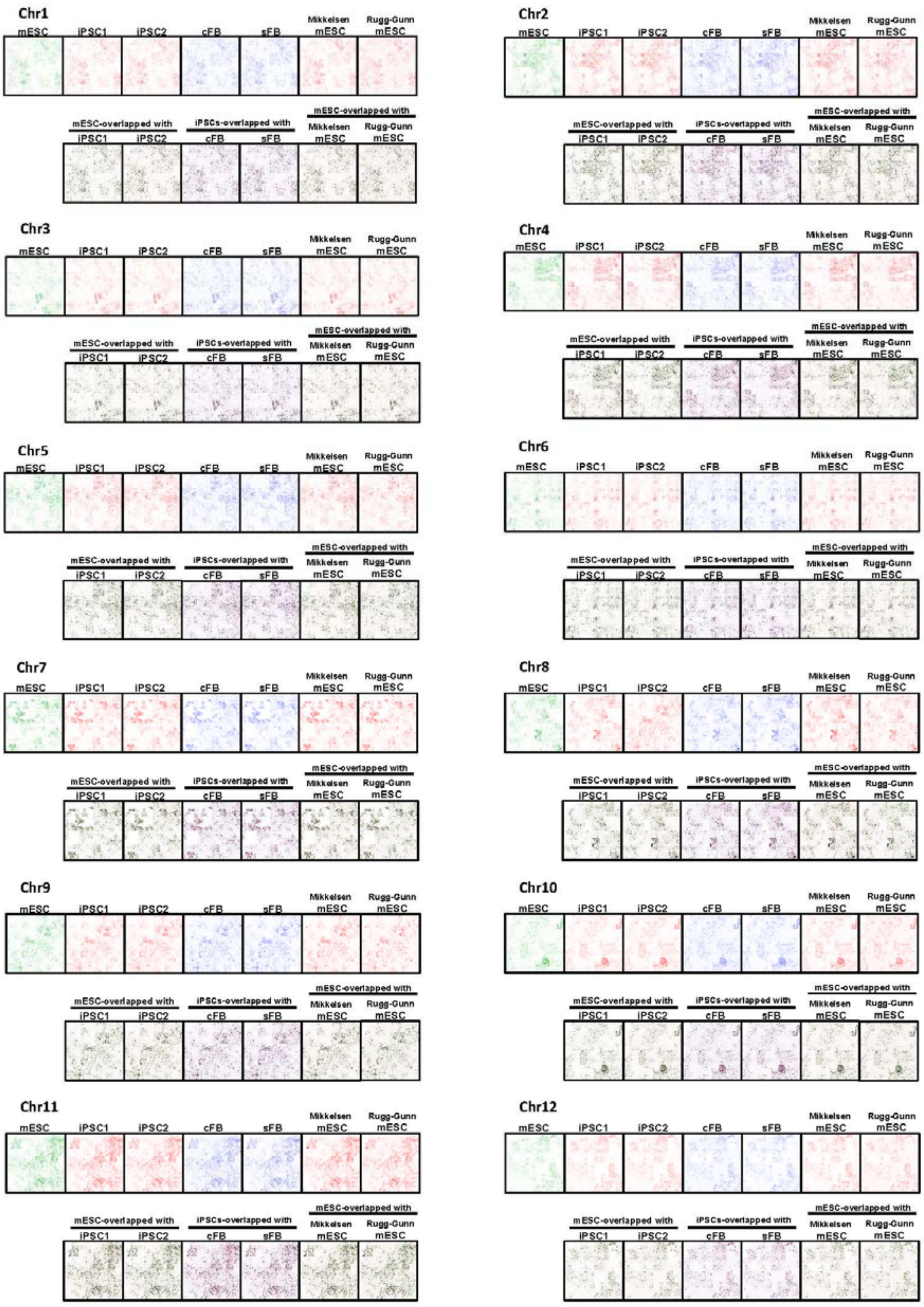

S1 Fig

A (continued)

H3K4me3

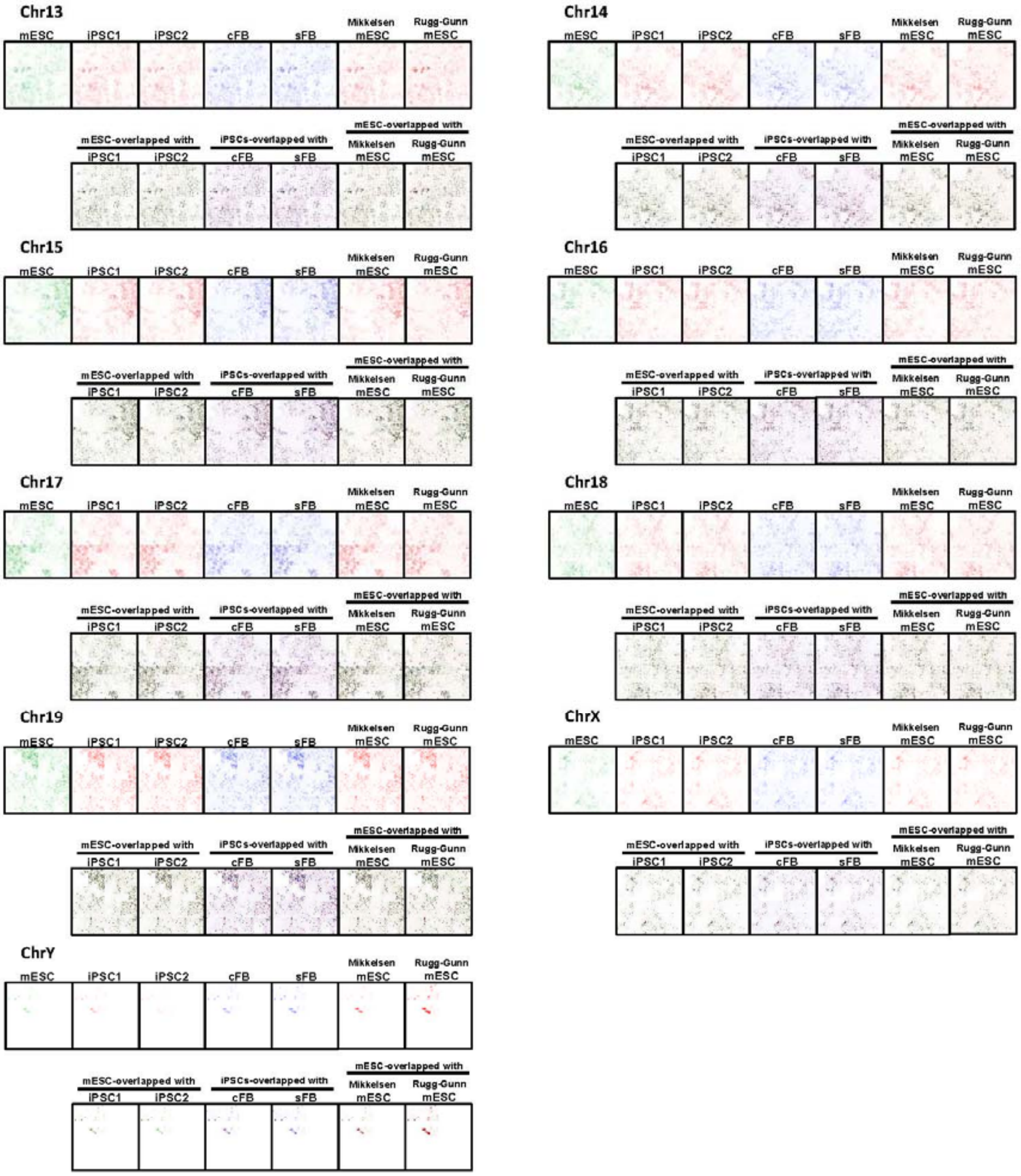

S1 Fig

B

H3K27me3

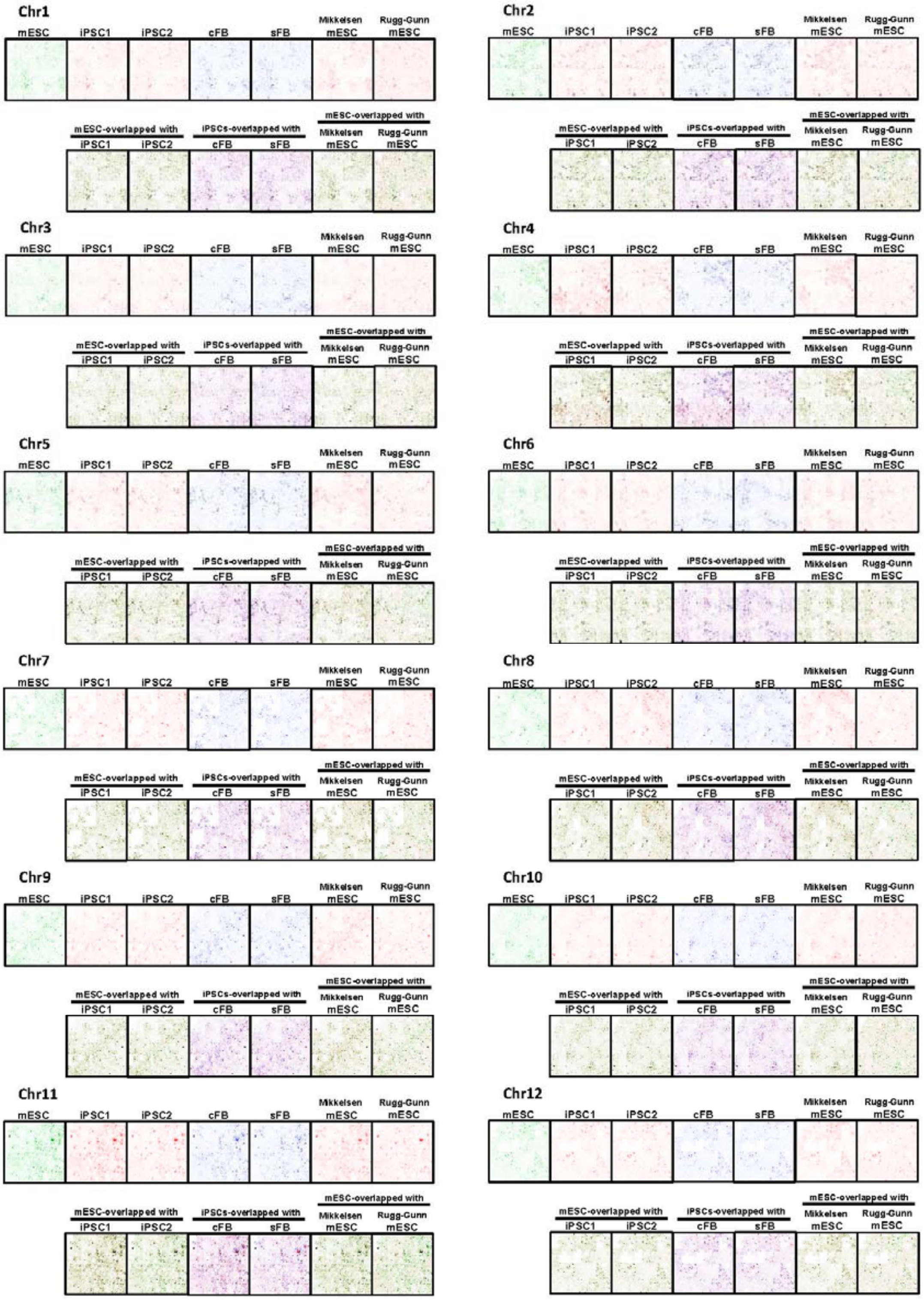

S1 Fig

B (continued)

H3K27me3

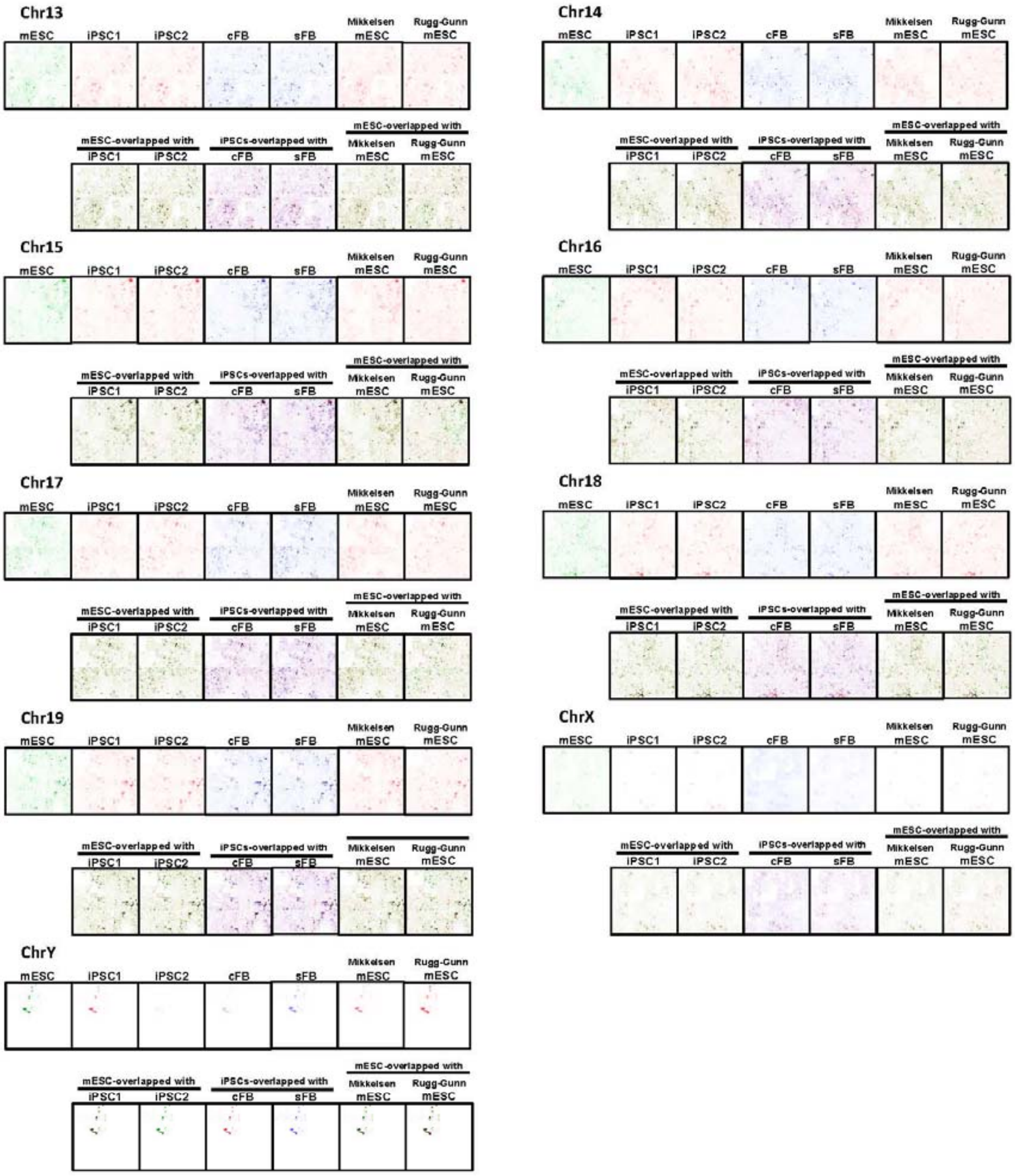

S1 Fig

C

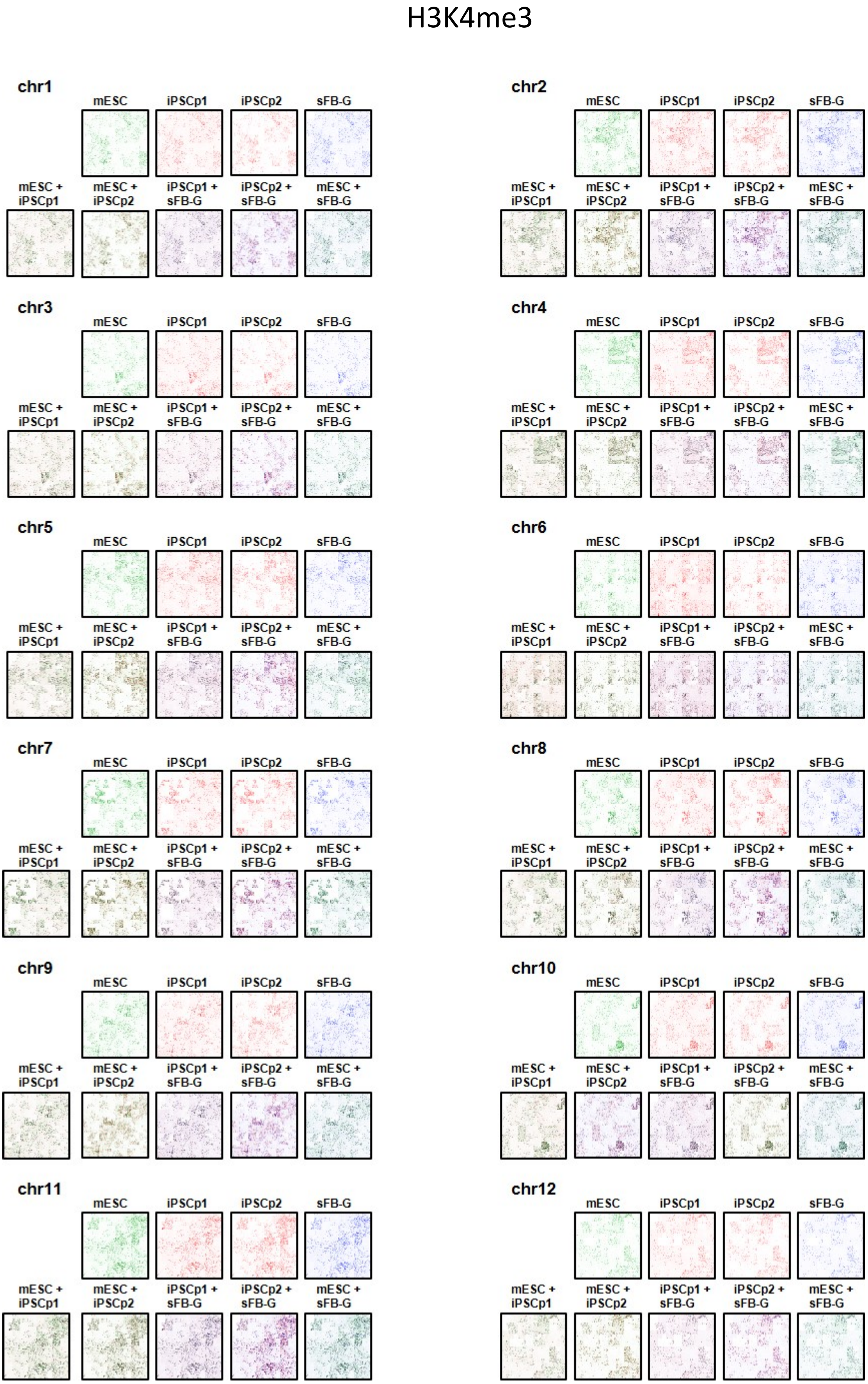
H3K4me3
chr1
chr2
chr3
chr4
chr5
chr6
chr7
chr8
chr9
chr10
chr11
chr12
mESC
iPSCp1
iPSCp2
sFB-G
mESC + iPSCp1
mESC + iPSCp2
iPSCp1 + sFB-G
iPSCp2 + sFB-G
mESC + sFB-G

S1 Fig

C (continued)

H3K4me3

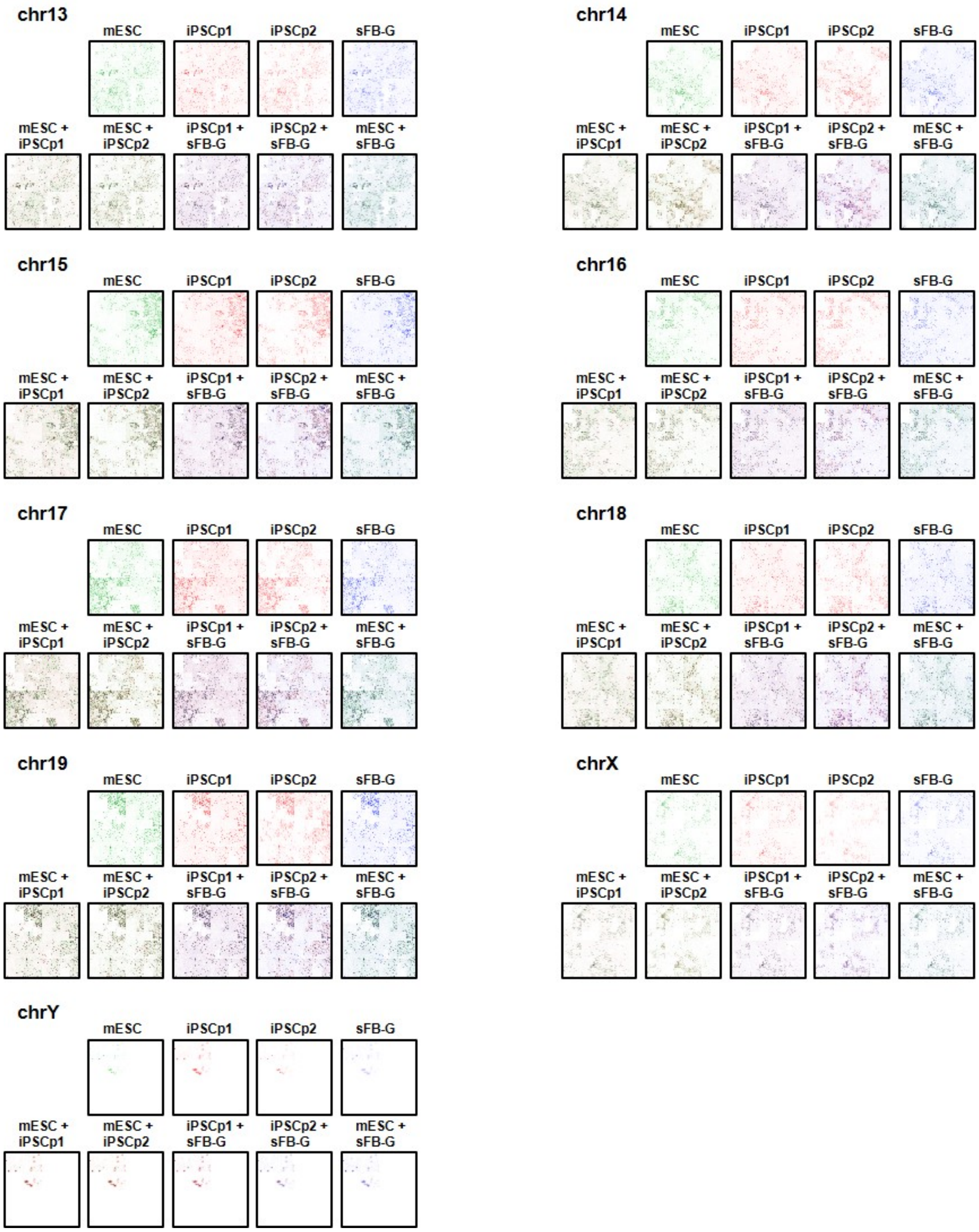

S1 Fig

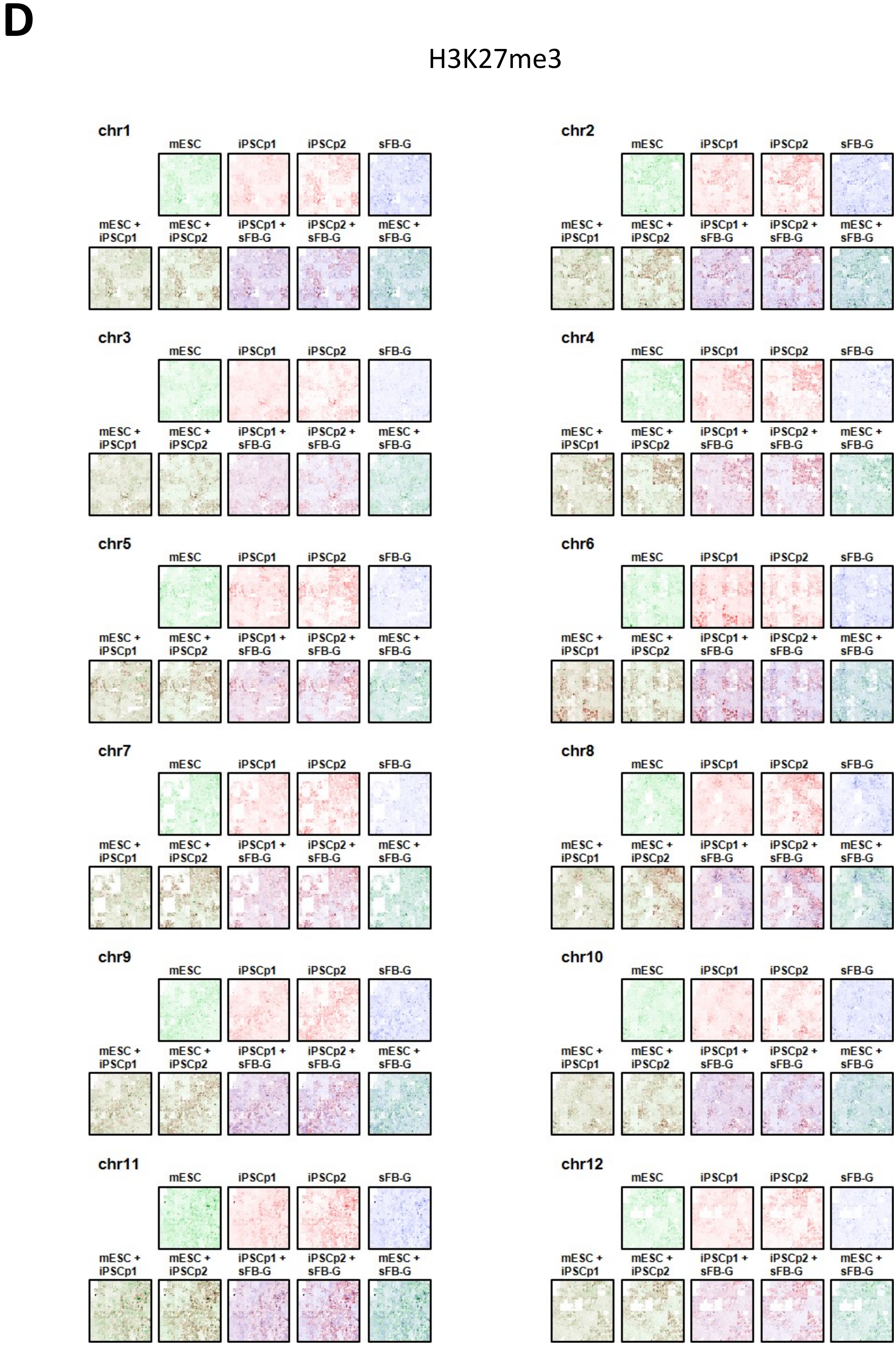
D
H3K27me3
chr1
mESC
iPSCp1
iPSCp2
sFB-G
mESC + iPSCp1
mESC + iPSCp2
iPSCp1 + sFB-G
iPSCp2 + sFB-G
mESC + sFB-G
chr2
chr3
chr4
chr5
chr6
chr7
chr8
chr9
chr10
chr11
chr12

S1 Fig

D (continued)

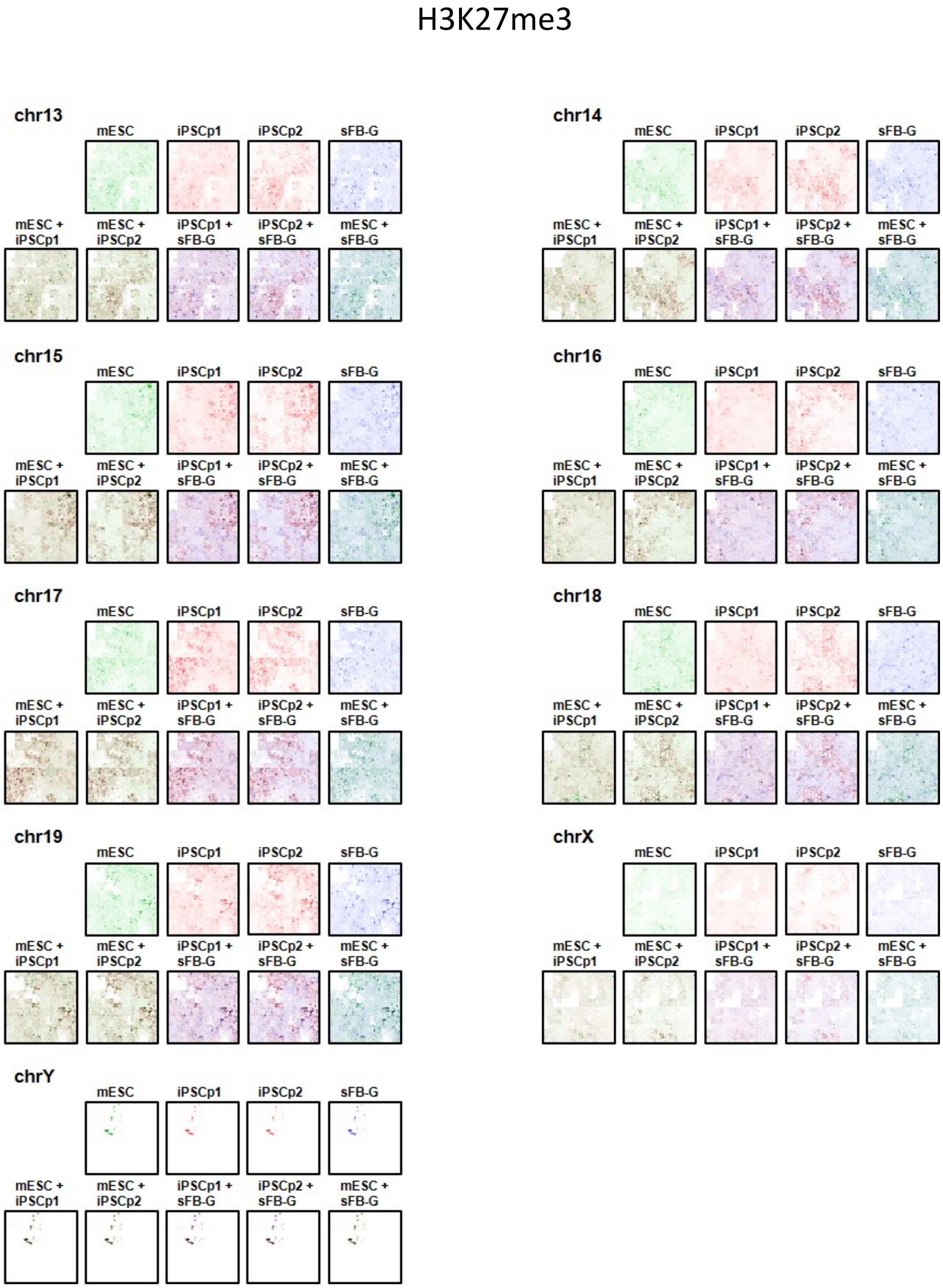

Supplement: S1 Fig — (PDF) [file pone.0171300.s001.pdf]
