# Supplementary figures and images for: Identification of the early and late responder genes during the generation of induced pluripotent stem cells from mouse fibroblasts
Source: PLoS One. 2017 Feb 2;12(2):e0171300. doi: 10.1371/journal.pone.0171300 (PMC5289558; doi:10.1371/journal.pone.0171300)

S2 Fig

**A**

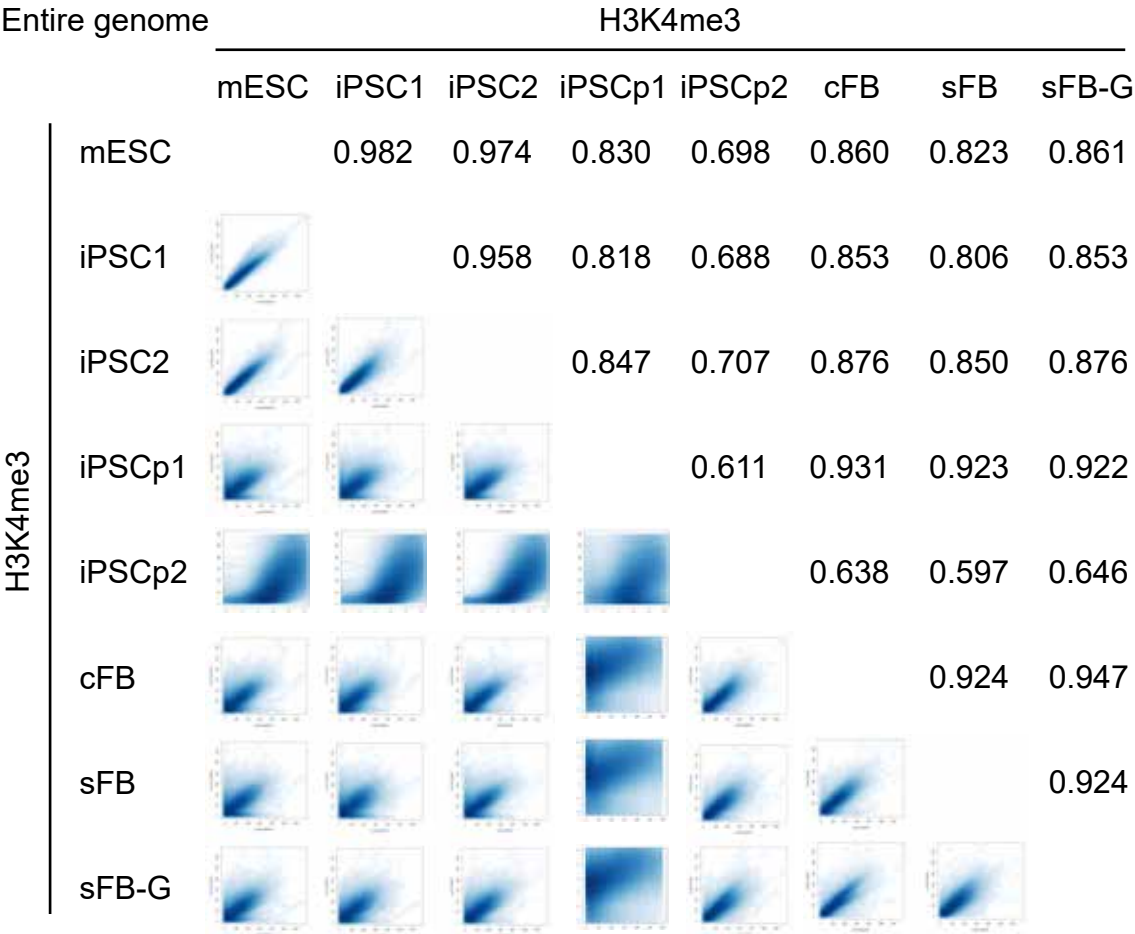

**B**

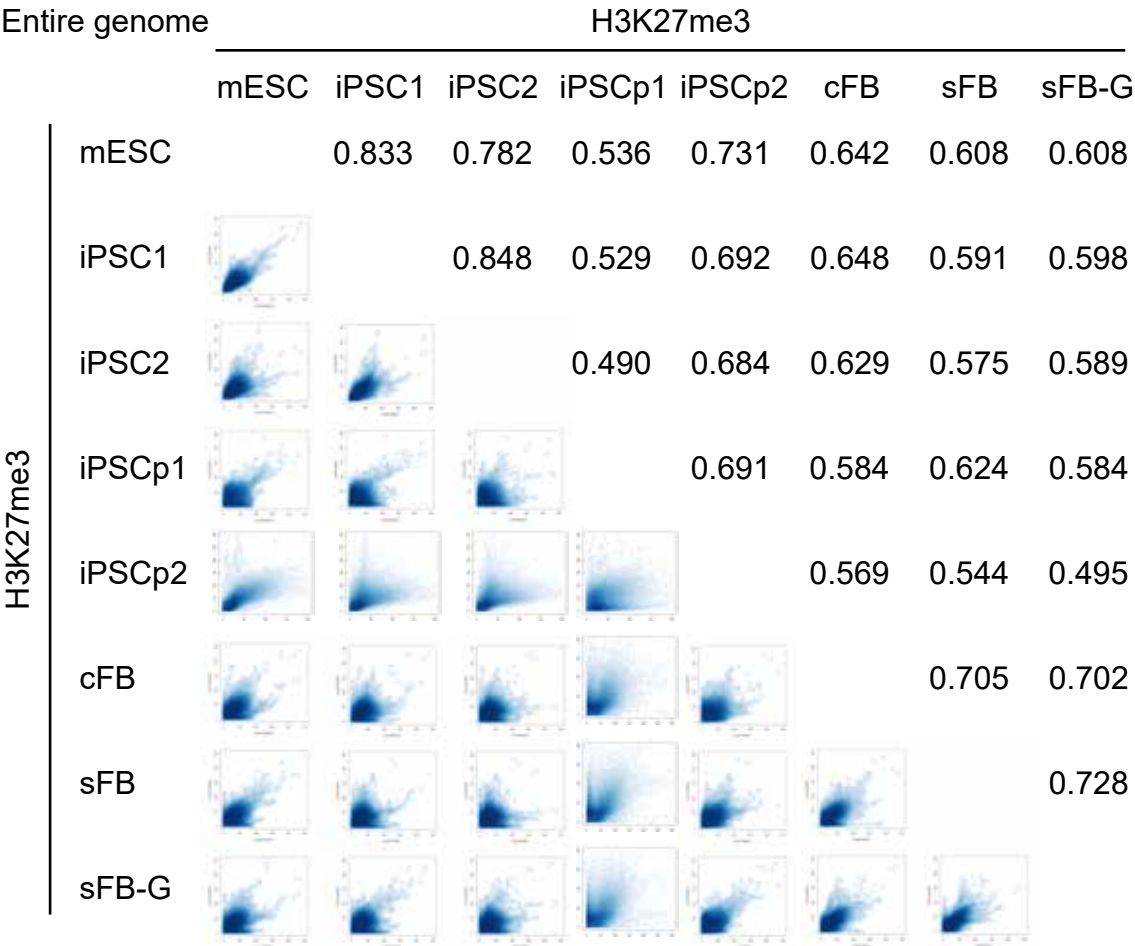

Supplement: S2 Fig — (PDF) [file pone.0171300.s002.pdf]

S3 Fig

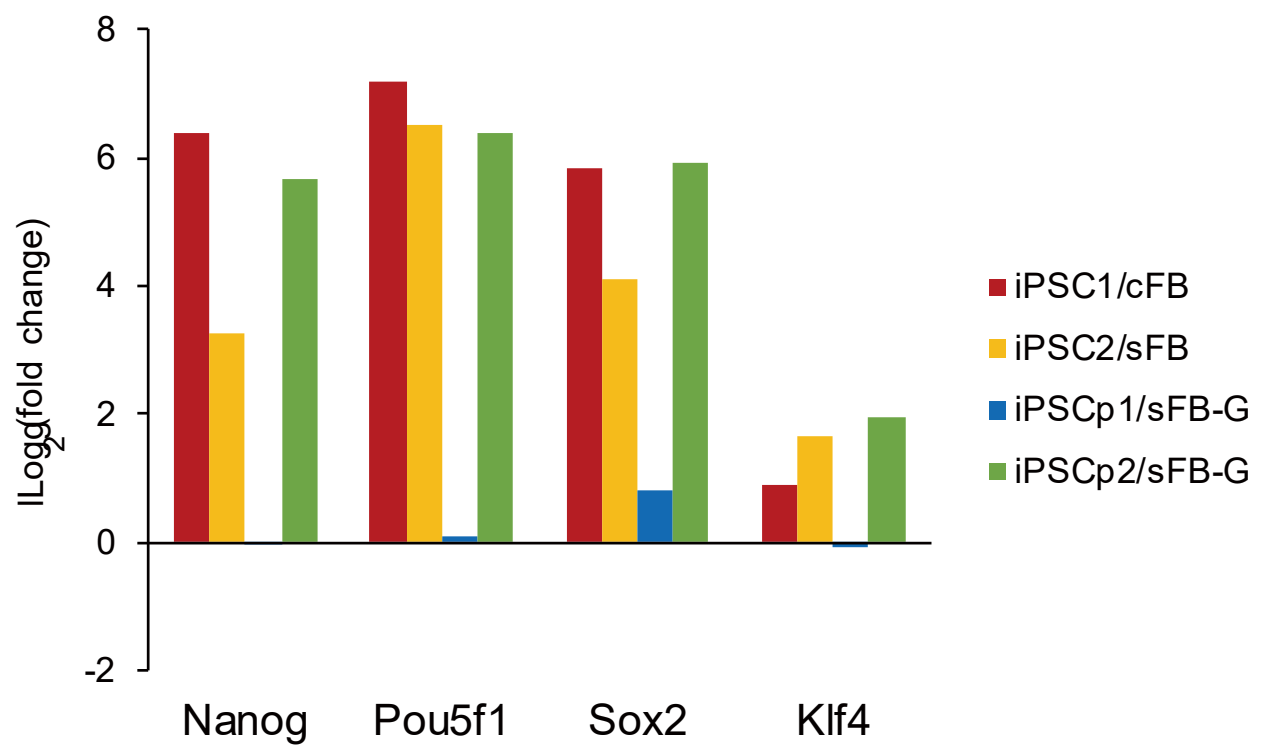

Supplement: S3 Fig — (PDF) [file pone.0171300.s003.pdf]

S4 Fig

**A**

**Up**

**iPSC1/cFB**

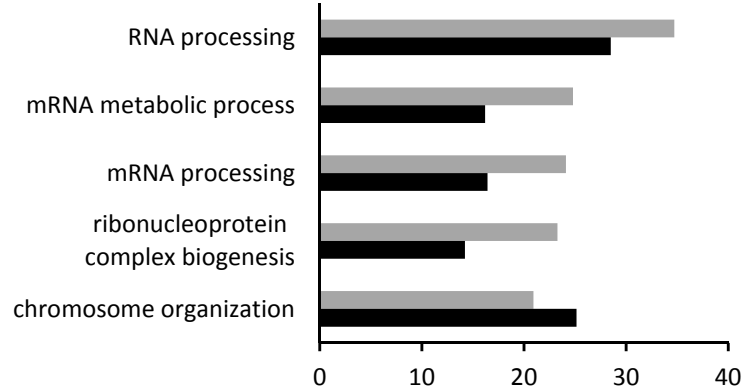

**B**

■ iPSCs/fibroblasts  
■ mESC/fibroblasts

**iPSC2/sFB**

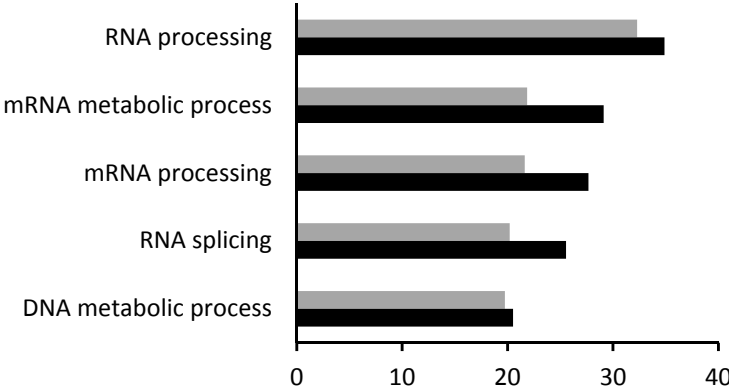

**C**

**Down**

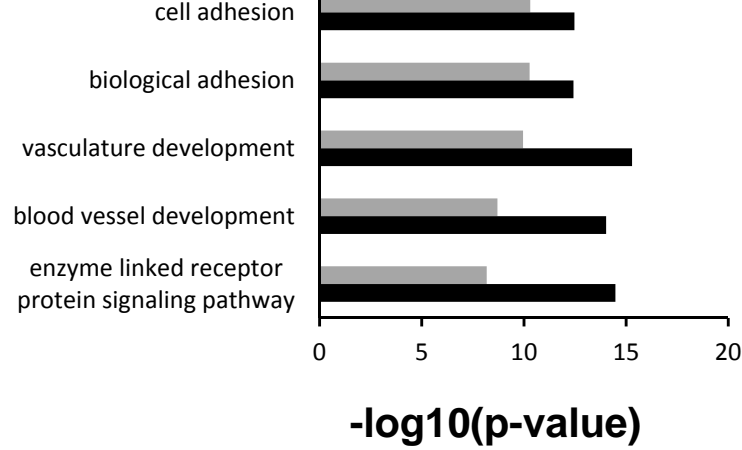

**D**

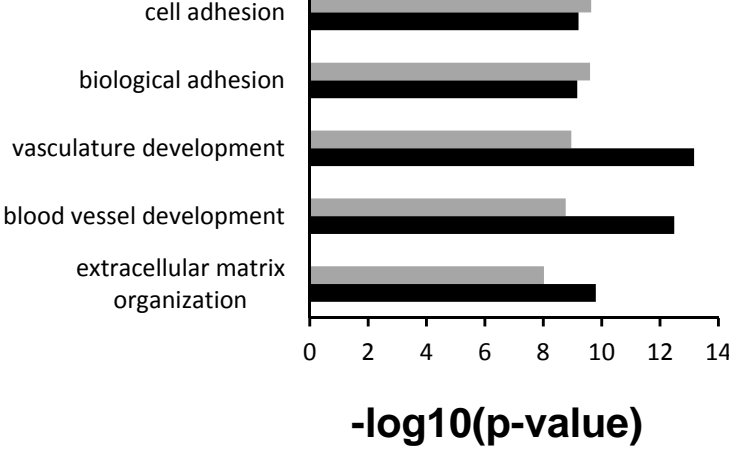

Supplement: S4 Fig — (PDF) [file pone.0171300.s004.pdf]

S5 Fig

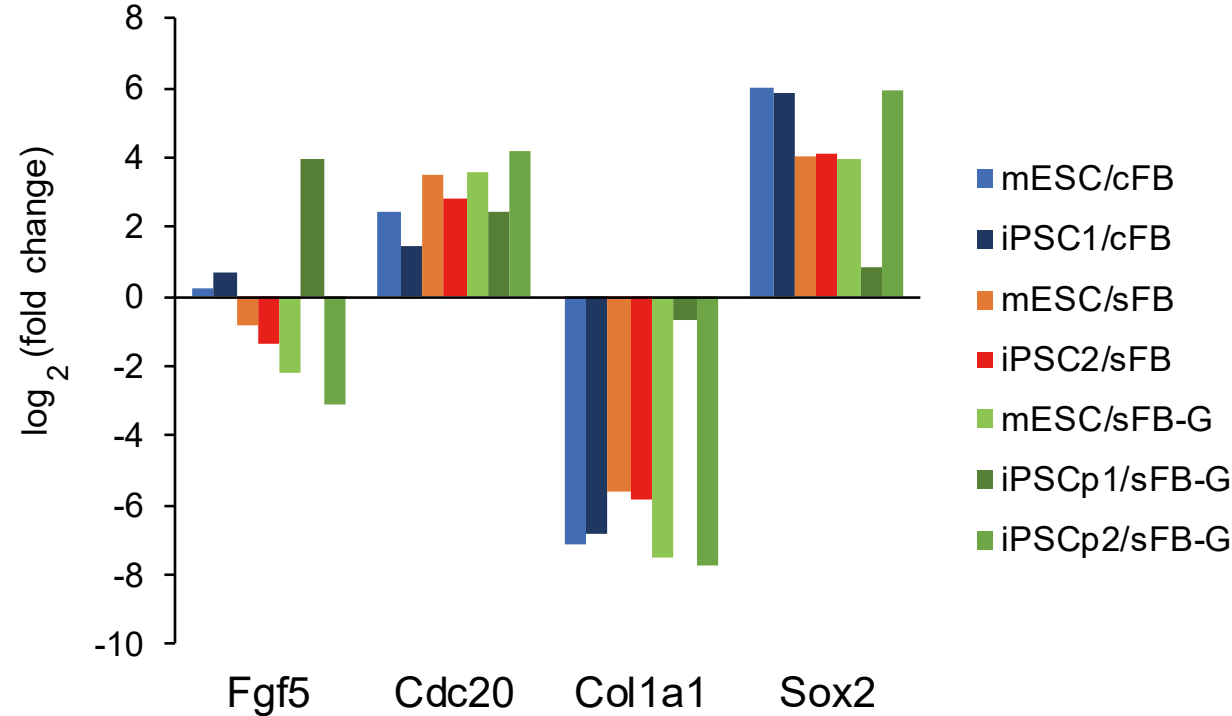

Supplement: S5 Fig — (PDF) [file pone.0171300.s005.pdf]

S6 Fig

A

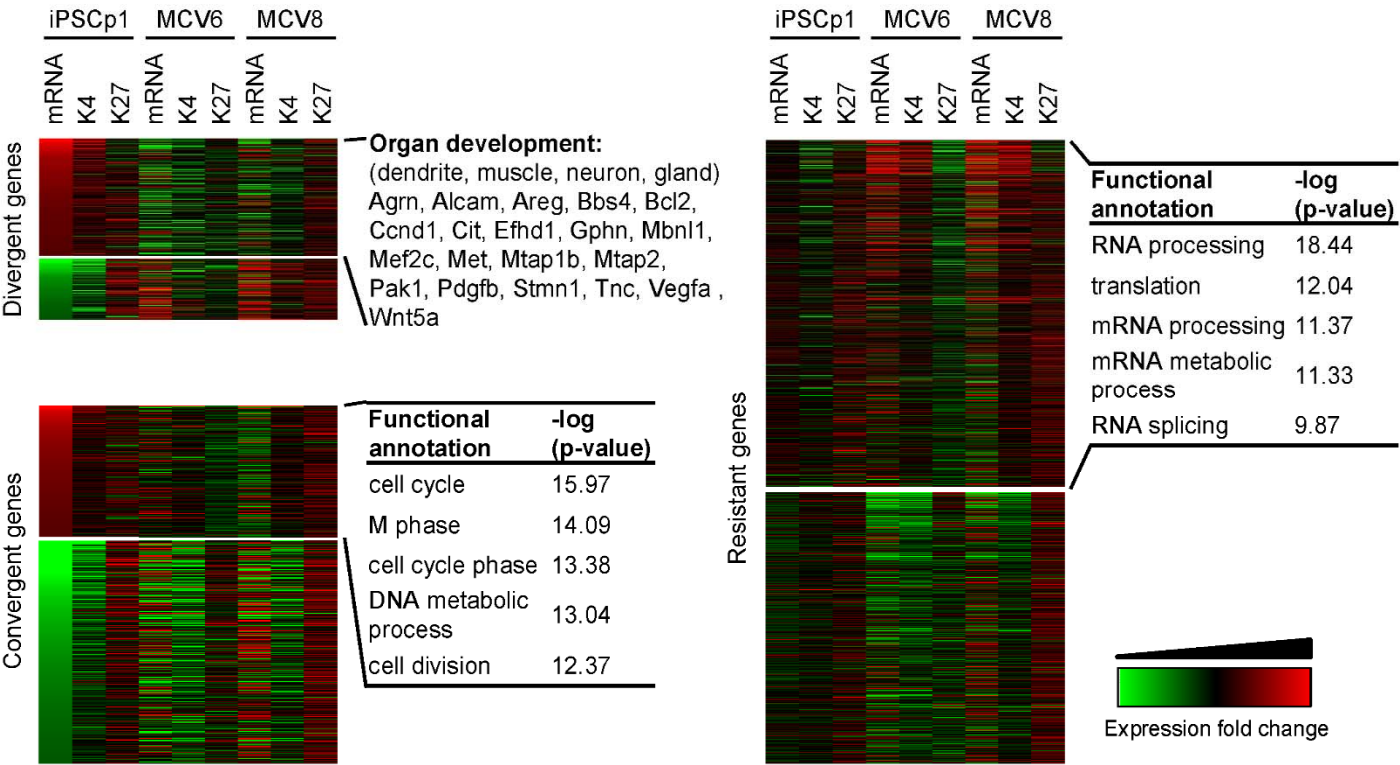

B

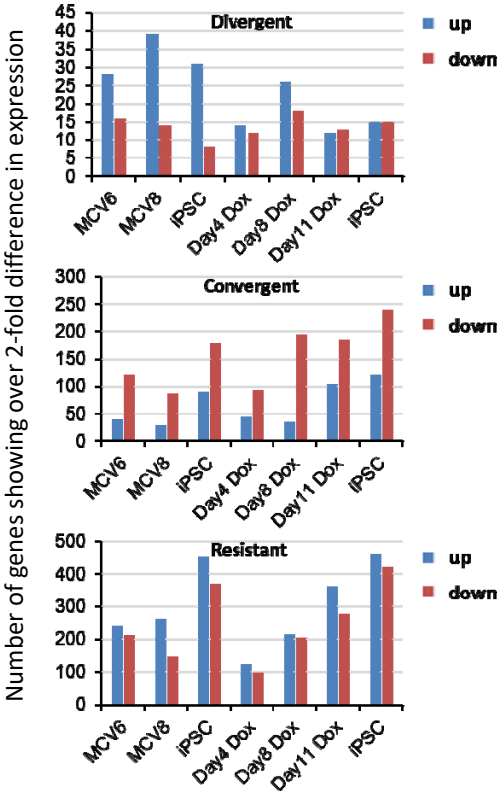

\* MCV6 and MCV8 data obtained from Nature. 2008 Jul 3;454(7200):49-55

\* Day4, 8, 11 Dox and IPSC in Fig. S6B from Nature. 2013 Oct 3;502 (7469):65-70

Supplement: S6 Fig — (PDF) [file pone.0171300.s006.pdf]
