# Supplementary material for: Identification of the early and late responder genes during the generation of induced pluripotent stem cells from mouse fibroblasts
Source: PLoS One. 2017 Feb 2;12(2):e0171300. doi: 10.1371/journal.pone.0171300 (PMC5289558; doi:10.1371/journal.pone.0171300)
Supplement: S1 Table — (PDF) [file pone.0171300.s007.pdf]

S1 Table. Divergent up

Gene information		mRNA fold change		H3K4me3 enrichment			H3K27me3 enrichment		
RefSeq gene	gene symbol	iPSCp/sFB-G	mESC/sFB-G	mESC	iPSCp	sFB-G	mESC	iPSCp	sFB-G
NM_010203	Fgf5	3.940	-2.189	1220	14664	231	2803	681	1029
NM_007945	Eps8	3.611	0.408	4649	14311	5786	3765	5085	3886
NM_133719	Metrn	3.282	0.195	1752	5569	2422	142	191	78
NM_009704	Areg	3.212	0.387	513	933	353	1067	445	841
NM_007421	Adssl1	3.123	0.115	2186	3530	1215	2344	738	561
NM_029662	Mfsd2	2.984	0.296	693	1972	1022	2020	447	405
NM_016701	Nes	2.876	-0.143	2785	9597	450	3049	381	730
NM_011857	Odz3	2.772	-0.679	8668	5982	2422	14173	11381	11713
NM_177914	Dgkk	2.708	-0.028	382	1186	498	2424	3310	3806
NM_001001979	Megf10	2.688	-1.514	1670	3940	2099	6047	3770	9223
NM_028186	Nkd2	2.648	-1.535	3074	4983	2588	2941	1305	1775
NM_032400	Sucnr1	2.638	-0.014	10	895	20	231	90	138
NM_008846	Pip5k1b	2.536	-0.038	10362	10663	9071	9332	11147	10869
NM_011990	Slc7a11	2.527	-1.295	223	2204	706	2361	1642	1110
NM_011252	Rbmx	2.504	-0.583	1638	2395	2093	38	50	70
NM_011267	Rgs16	2.458	-1.689	1025	13659	2908	812	223	154
NM_001033484	Iqgap3	2.421	0.205	1993	1849	1069	947	941	361
NM_025282	Mef2c	2.409	-0.503	1254	7683	2398	6786	3785	6653
NM_175096	Stbd1	2.381	-0.304	1414	1747	1372	700	536	170
NM_007603	Capn6	2.366	-0.760	134	968	154	541	645	906
NM_025459	Fam134b	2.357	0.601	247	394	291	1291	1051	3471
NM_027881	Osbpl3	2.351	0.578	946	1618	842	2173	2678	2229
NM_008632	Mtap2	2.251	0.151	2348	2998	2107	8290	5287	11928
NM_008398	Itga7	2.237	-0.191	1280	3941	912	2205	874	927
NM_019971	Pdgfc	2.220	0.189	4546	7310	4110	3927	3144	2983

NM_178772	Aadacl1	2.152	-1.347	2970	5218	3072	1725	1432	839
NM_013703	Vldlr	2.138	0.003	3122	4799	2751	2214	1399	2442
NM_001012401	Hspb6	2.137	-1.803	1280	2364	1465	86	14	61
NM_008343	Igfbp3	2.039	0.822	2983	2929	1582	1627	469	1328
NM_009610	Actg2	2.007	-1.415	245	489	264	1343	1661	1785
NM_133897	Lrrc8c	1.981	0.351	3413	6296	2230	3010	3150	1456
NM_021604	Agrn	1.973	0.585	1211	413	119	522	465	247
NM_011057	Pdgfb	1.946	-0.072	4538	8332	3614	3685	1646	2827
NM_019634	Tspan7	1.925	-0.432	791	1694	1316	1879	1971	2243
NM_145990	Cdk5rap2	1.912	0.790	4077	5060	2599	3834	5114	3517
NM_013749	Tnfrsf12a	1.902	-0.097	2671	5874	4741	491	201	208
NM_133187	1110032E23Rik	1.902	-1.427	532	10719	3016	1679	901	790
NM_016721	Iqgap1	1.846	0.129	5018	6566	4095	2117	1642	913
NM_028889	Efhd1	1.839	0.453	2647	1141	1146	4676	2448	5118
NM_021278	Tmsb4x	1.816	0.064	1593	3022	2095	59	25	41
NM_144862	Lims2	1.808	-0.950	2255	2210	2027	3117	1262	1923
NM_013468	Ankrd1	1.795	0.829	52	728	75	509	354	341
NM_027711	Iqgap2	1.792	0.852	4363	4314	3924	9400	8232	14594
NM_009468	Dpysl3	1.790	0.165	3018	3030	2468	2001	1455	2350
NM_172628	Sh3tc2	1.783	0.108	870	2650	1269	2184	1729	1872
NM_008761	Fxyd5	1.776	-1.063	215	1448	1249	1154	200	90
NM_007631	Ccnd1	1.775	-0.985	4410	6369	3360	1274	294	35
NM_019972	Sort1	1.772	0.423	2618	3026	1696	2859	2461	1493
NM_011828	Hs2st1	1.753	-0.274	6372	8953	5425	2887	2819	1544
NM_007498	Atf3	1.749	-1.220	4801	6138	4544	2547	323	816
NM_021324	Ttyh1	1.747	-0.297	2026	2769	983	642	879	423
NM_207650	Dtna	1.738	0.354	1373	5935	1814	7589	6963	12891
NM_008641	Mast2	1.735	-0.482	3085	5056	3068	2390	2458	1926
NM_172637	Hectd2	1.734	-0.336	3577	3706	3516	2292	1961	2643

NM_011123	Plp1	1.724	-1.824	20	2995	116	229	465	203
NM_008216	Has2	1.700	-0.945	1504	6351	5254	1573	878	1193
NM_009103	Rrm1	1.693	0.834	2536	3352	2082	324	505	434
NM_023476	Tinagl	1.661	0.075	798	1095	774	1558	462	174
NM_207209	Sec24b	1.657	-0.471	3218	5923	3496	1287	1325	853
NM_011824	Grem1	1.649	-2.505	2075	6171	4699	1974	320	1042
NM_052993	C1galt1	1.647	-0.303	1593	7725	3624	601	935	744
NM_080558	Ssfa2	1.620	-0.584	4454	6860	4729	953	474	892
NM_009784	Cacna2d1	1.619	0.416	3230	4884	2649	9365	8663	7699
NM_019636	Tbc1d1	1.615	0.785	7098	8883	5554	8068	4218	2277
NM_197990	1700025G04Rik	1.615	-0.310	5110	5759	6518	6926	4305	5618
NM_011893	Sh3bp2	1.613	-1.440	3287	5334	4311	2651	1095	383
NM_022890	Cldn12	1.599	0.638	2601	5300	2694	281	188	117
NM_013546	Hebp1	1.594	0.218	2723	3001	1819	1602	1119	1052
NM_001040088	Sytl2	1.585	-2.790	142	136	373	685	549	503
NM_177876	Vps37b	1.580	0.344	2928	5944	3621	657	673	216
NM_026470	Spata6	1.576	-0.643	1612	4171	2129	1671	1422	1413
NM_001033198	Ankrd50	1.570	0.433	83	37	28	122	152	66
NM_021389	Sh3kbp1	1.569	-2.082	705	2211	1419	5281	3599	3245
NM_133942	Plekha1	1.563	0.899	4123	4792	3201	1000	857	656
NM_007610	Casp2	1.561	0.168	2301	5861	3257	328	471	513
NM_146150	Nrd1	1.560	0.933	5030	5530	3655	1159	1056	745
NM_027828	Fam110c	1.558	-0.397	1311	1569	1887	1036	602	582
NM_011738	Ywhah	1.531	0.544	3861	6686	2906	372	153	84
NM_001081229	Tsc22d2	1.529	0.137	7796	21298	11462	1337	757	800
NM_008591	Met	1.528	-0.253	2430	8303	3844	4849	3049	2907
NM_010699	Ldha	1.526	0.655	1796	2975	2583	239	173	93
NM_009415	Tpi1	1.525	0.388	1283	6555	3701	70	156	49
NM_009673	Anxa5	1.515	-2.982	2575	9197	3952	1595	741	204

NM_007943	Eps15	1.513	0.433	2588	4326	2423	1933	1680	1206
NM_007666	Cdh6	1.508	-0.191	2093	3884	1923	7457	4707	5045
NM_008638	Mthfd2	1.503	0.784	3546	7243	4285	165	529	285
NM_011607	Tnc	1.502	-3.569	451	763	379	2186	1454	1041
NM_172579	Sipa1l1	1.502	-0.076	1661	1599	1120	3810	4975	2953
NM_025730	Lrrk2	1.501	-1.360	969	7548	4369	5489	4005	4144
NM_178726	Ppm1l	1.500	0.318	5023	4937	3472	7654	5138	4727
NM_021385	Rad18	1.482	0.414	3380	4892	2826	1233	1675	1567
NM_009524	Wnt5a	1.466	-0.409	2702	5603	5601	5343	915	1184
NM_030249	Cttnbp2nl	1.450	0.085	2674	6305	3196	959	1064	535
NM_008590	Mest	1.428	-0.603	163	1789	721	227	542	1358
NM_019958	Rgs17	1.423	-0.321	2141	2066	3530	3834	3176	3036
NM_007759	Crabp2	1.420	0.852	583	592	161	974	128	375
NM_009655	Alcam	1.417	-0.017	1903	5965	3204	5847	5001	14202
NM_010833	Msn	1.413	-0.569	1126	6863	4283	821	851	844
NM_175093	Trib3	1.372	0.147	3657	7200	3269	305	128	136
NM_027652	D5Wsu178e	1.357	0.045	1862	4640	2956	1069	742	471
NM_009274	Srpk2	1.351	0.881	821	3471	1578	1961	2299	1564
NM_016795	Srpk1	1.351	0.995	5583	5565	6317	698	1026	645
NM_138741	Sdpr	1.342	-1.098	189	3823	1601	877	267	503
NM_013898	Timm8a1	1.333	0.716	1178	1627	2021	37	40	38
NM_145495	Rin1	1.331	-1.440	1822	6776	5216	560	232	186
NM_011863	Papss1	1.326	-1.986	1730	3186	2033	1719	1821	746
NM_001031814	2610207I05Rik	1.325	0.009	5017	5243	3809	1252	2318	1458
NM_172865	Manea	1.323	-1.741	2662	4186	2469	404	413	288
NM_145494	Me2	1.322	-0.022	3276	4026	3652	1099	1392	1179
NM_053123	Smarca1	1.317	0.748	1312	1507	1250	1582	1527	2281
NM_026883	1500011H22Rik	1.308	0.901	4175	3140	2714	228	255	128
NM_026632	Rpa3	1.296	0.759	3583	4876	4286	60	84	66

NM_025674	Tcf19	1.295	-1.164	1111	4234	4292	155	230	115
NM_007948	Ercc1	1.292	0.913	2080	1627	1621	264	260	114
NM_027418	Mapk6	1.288	-0.237	4321	8425	6088	516	720	457
NM_133919	Aff1	1.283	0.552	5347	8363	4858	3042	1845	1240
NM_001038701	Gabrb3	1.283	0.145	4514	2750	1166	5145	5025	4710
NM_008654	Myd116	1.282	-0.665	3639	8660	4020	127	48	10
NM_007837	Ddit3	1.275	-2.073	4333	14807	5858	160	193	114
NM_010723	Lmo4	1.269	-1.697	3613	10137	5222	2678	336	205
NM_019581	Gtpbp2	1.267	-1.649	2031	6670	3265	242	430	274
NM_173402	Rgs12	1.266	0.180	3543	4448	2985	3469	2261	1113
NM_009017	Raet1b	1.265	0.553	1309	2127	1472	5292	5753	4030
NM_133348	Acot7	1.264	0.341	3948	5514	3997	3228	2150	885
NM_019390	Lmna	1.261	-1.241	1315	1980	879	1101	239	64
NM_197981	5930416I19Rik	1.254	0.745	3442	5546	3850	128	93	77
NM_028979	Cyp2j9	1.252	-1.013	192	1624	597	600	682	551
NM_011843	Mbc2	1.246	-0.453	4011	8391	6051	823	599	378
NM_007836	Gadd45a	1.233	-0.098	7435	12399	5814	81	182	66
NM_029437	Ckap5	1.230	-0.154	243	316	392	985	1124	1504
NM_138747	Nol1	1.228	0.654	5379	5461	4687	277	588	258
NM_001034964	Sorbs1	1.226	-0.651	1976	4936	1391	2894	3208	3046
NM_183315	Ctxn1	1.217	0.448	2364	1931	1495	81	87	21
NM_011877	Ptpn21	1.217	-0.589	1765	2645	1609	1929	1379	630
NM_001025250	Vegfa	1.216	-0.349	5076	13563	9395	1352	546	391
NM_011785	Akt3	1.207	-0.236	558	1099	1069	4504	3916	5867
NM_026580	Otub2	1.201	0.039	2070	3738	1910	519	539	241
NM_010863	Myo1b	1.196	-0.071	6313	5091	5470	4229	4376	3972
NM_019670	Diap3	1.193	0.863	3978	4681	6076	7658	7978	12222
NM_175306	Phactr4	1.189	0.086	3564	4778	3060	1112	1335	590
NM_177730	Impad1	1.185	0.539	3531	4573	3363	456	520	377

NM_001040695	Uevld	1.181	0.371	2828	2801	2346	542	637	392
NM_009741	Bcl2	1.180	-0.260	3218	4654	3151	7103	4345	11450
NM_008350	Il11	1.176	0.306	898	630	530	1088	253	255
NM_198111	Akap6	1.173	-0.283	2762	6143	2209	16384	11053	12154
NM_029166	Uhrf1bp1l	1.172	-0.003	2953	5628	4011	1477	1938	1726
NM_011319	Sars	1.167	0.673	3997	7012	3054	311	379	184
NM_030723	Pum2	1.165	0.778	3199	5239	2630	1078	1661	1269
NM_053090	Drctnnb1a	1.161	-0.556	3753	4325	3523	2049	1549	881
NM_008188	Thumpd3	1.157	0.710	4747	6921	4199	274	640	579
NM_026384	Dgat2	1.155	0.355	1335	1460	1081	2720	963	545
NM_008862	Pkia	1.154	-2.218	1232	2432	1424	2104	1366	1190
NM_009029	Rb1	1.153	0.592	5531	8914	8848	3080	2869	3695
NM_023117	Cdc25b	1.150	0.130	2106	3067	3512	421	201	208
NM_024433	Mtap	1.146	0.006	3394	4828	3184	779	1229	436
NM_027295	Rab28	1.144	-0.942	4034	5059	3828	1238	1463	1056
NM_145578	Ube2m	1.142	0.826	4343	4374	3363	91	129	18
NM_134054	1110002B05Rik	1.142	-0.424	2724	3641	2461	467	325	207
NM_198411	2610204M08Rik	1.139	-1.437	2604	5678	4463	2431	922	276
NM_134094	Ncald	1.136	-1.241	4699	8422	6002	19805	25301	15252
NM_172694	Megf9	1.135	-0.768	3353	7051	3698	3114	1783	1829
NM_173764	Tapt1	1.134	-0.264	4199	4464	2835	1195	1307	643
NM_008994	Pex2	1.133	-1.367	2444	4746	2920	600	294	223
NM_183171	Fez1	1.131	-1.493	252	257	255	1283	716	817
NM_007981	Acsl1	1.129	0.676	3449	3220	2437	2174	1835	1048
NM_009088	Polr1a	1.127	0.688	3490	5314	3216	989	2126	1472
NM_177282	Mical2	1.127	-0.922	1773	4286	5177	6960	3045	1317
NM_172952	Gphn	1.125	-0.113	6216	6377	5508	9319	6978	7330
NM_198710	Sypl	1.123	-0.454	2803	3848	2715	727	494	408
NM_025451	Camk2n1	1.123	-0.207	5361	5106	2877	878	278	176

NM_009465	Axl	1.122	-3.781	922	6201	3463	1701	731	495
NM_007708	Cit	1.118	0.412	5841	4033	2425	3642	3578	1692
NM_009156	Sepw1	1.116	0.096	3208	5882	3193	209	162	48
NM_012055	Asns	1.116	0.857	4943	8380	4267	279	504	452
NM_016661	Ahcy	1.113	-0.216	2546	2170	2211	263	307	318
NM_198420	D15Wsu169e	1.113	0.375	6685	6607	6878	2821	2956	2718
NM_144526	6720460F02Rik	1.113	0.325	2028	3149	2482	481	101	111
NM_016807	Sdcbp	1.112	-1.794	1620	5150	3015	452	458	286
NM_011977	Slc27a1	1.109	-0.228	5429	2893	2249	991	434	690
NM_008380	Inhba	1.107	-1.851	186	5931	6076	1671	280	580
NM_001025438	Camk2d	1.103	-0.825	5398	12397	6998	6247	4727	2775
NM_020007	Mbnl1	1.098	-1.789	3367	11060	5674	2560	2246	1868
NM_146152	Ipo13	1.098	-0.457	4088	5789	4447	562	264	166
NM_011779	Coro1c	1.095	0.916	5960	5353	3571	1449	1769	671
NM_013509	Eno2	1.094	-1.507	2525	4606	3974	939	429	254
NM_011035	Pak1	1.091	0.946	423	676	893	1591	1106	1130
NM_019914	Mllt11	1.084	-1.474	3915	7137	4377	161	383	84
NM_175937	Cpeb2	1.081	-0.194	5422	12203	5981	1767	820	733
NM_019471	Mmp10	1.078	-0.280	50	32	88	145	156	394
NM_019808	Pdlim5	1.073	0.264	3166	6448	3869	2550	2900	2339
NM_153563	6330569M22Rik	1.072	0.443	2929	3517	3057	539	509	215
NM_134006	Rdh5	1.068	-0.193	180	1164	539	112	259	181
NM_024495	Car13	1.068	0.162	709	2151	1181	500	491	346
NM_026878	Rasl11b	1.067	0.813	3151	3628	2037	259	256	178
NM_009506	Vegfc	1.062	-1.116	3106	4432	2992	1535	1699	1670
NM_010470	Hp1bp3	1.060	-1.108	7243	6759	4496	482	689	192
NM_011100	Prkacb	1.055	-0.388	4982	6151	4293	1601	1742	979
NM_030690	Rai14	1.054	0.419	6431	7171	5383	2802	4516	4273
NM_029337	Ep400	1.050	0.701	4674	3908	3100	1616	2219	1236

NM_008634	Mtap1b	1.049	-0.333	5236	9461	9789	3006	2562	2296
NM_001045514	Akna	1.049	0.458	432	1201	1447	1309	1140	434
NM_053084	Trim32	1.046	-0.328	1559	2597	1537	433	210	165
NM_175325	Bbs4	1.046	0.898	1245	1736	1548	589	474	641
NM_146258	Stard13	1.040	-3.183	1328	15412	7301	5516	3060	3611
NM_178061	Mobkl2b	1.035	0.965	5285	2731	2062	7164	6983	9966
NM_021494	Dennd5a	1.034	-1.221	3209	5084	2686	1336	1310	711
NM_008624	Mras	1.033	-0.252	2745	3978	4682	3196	1031	1123
NM_144900	Atp1a1	1.033	0.939	3974	5659	3585	596	792	428
NM_008397	Itga6	1.032	0.883	5221	4867	3268	1649	1525	6172
NM_028410	Prkrir	1.028	-0.053	3313	4510	3034	277	66	199
NM_019641	Stmn1	1.023	0.704	2547	4202	2263	246	106	94
NM_133927	Itfg2	1.021	-0.159	2193	2861	1990	359	546	242
NM_028013	Endod1	1.020	-2.232	2444	3570	3310	1684	591	917
NM_172476	Tmc7	1.019	0.890	1507	2242	1591	3252	1547	872
NM_176841	Ccdc88a	1.017	-0.433	4086	4857	5395	3198	2871	3792
NM_007530	Bcap29	1.016	-0.216	1359	2468	1106	783	755	719
NM_030706	Trim2	1.012	0.759	3630	2953	978	2102	1205	764
NM_011942	Lypla2	1.010	0.329	3253	4142	2957	107	105	43
NM_029621	2410004L22Rik	1.010	0.536	2685	2721	4044	443	382	349
NM_008487	Arhgef2	1.008	0.206	4029	9133	4023	879	854	181
NM_025829	Eif4e3	1.007	-1.261	2005	4491	3528	3891	2535	1430
NM_008536	Tm4sf1	1.007	-0.421	80	9275	2064	628	201	197
NM_001039057	Kcnj15	1.007	-0.914	354	3077	571	1594	1186	1509
NM_153489	Ubap2l	1.004	0.932	4392	5091	3894	843	811	519
NM_023290	Mkrn2	1.003	0.821	2026	3074	2250	387	683	295
NM_011629	Nr2c1	1.003	-0.083	2361	5844	4472	1799	1372	1545
