# Supplementary material for: Identification of the early and late responder genes during the generation of induced pluripotent stem cells from mouse fibroblasts
Source: PLoS One. 2017 Feb 2;12(2):e0171300. doi: 10.1371/journal.pone.0171300 (PMC5289558; doi:10.1371/journal.pone.0171300)
Supplement: S2 Table — (PDF) [file pone.0171300.s008.pdf]

S2 Table. Convergent up genes

Gene information		mRNA fold change		H3K4me3 enrichment			H3K27me3 enrichment		
RefSeq gene	gene symbol	iPSCp/sFB-G	mESC/sFB-G	mESC	iPSCp	sFB-G	mESC	iPSCp	sFB-G
NM_010686	Laptm5	3.728	3.584	1062	512	152	901	1293	597
NM_010052	Dlk1	3.435	1.765	928	400	212	1721	1077	972
NM_008800	Pde1b	3.339	1.756	1915	2764	1131	2361	1893	2854
NM_008726	Nppb	3.064	2.449	465	881	42	65	54	29
NM_178701	Lrrc8d	3.037	2.912	6646	6349	3439	2117	2772	2126
NM_007681	Cenpa	3.015	3.567	4379	4094	1854	154	261	107
NM_009122	Satb1	2.916	1.780	5763	5778	2630	6046	6793	7428
NM_011670	Uchl1	2.845	2.740	3381	1828	718	342	682	1062
NM_008808	Pdgfa	2.768	2.480	7730	10298	5285	1440	417	940
NM_134471	Kif2c	2.574	2.817	3824	3248	1457	446	306	126
NM_001042421	Kntc1	2.562	2.268	2826	2815	1609	1186	1557	764
NM_023223	Cdc20	2.451	3.535	3477	3436	1787	67	30	37
NM_008905	Ppfibp2	2.439	1.207	3659	4503	902	3898	3992	3737
NM_178382	Flrt3	2.423	1.322	502	4106	1331	763	402	1133
NM_001037279	2700094K13Rik	2.417	2.347	4561	3189	3414	59	32	23
NM_019499	Mad2l1	2.400	2.293	3467	5955	3206	84	96	101
NM_013726	Dbf4	2.370	3.166	5694	5016	3231	426	398	328
NM_021788	Sap30	2.355	3.677	5414	5118	3716	155	154	98
NM_026560	Cdca8	2.324	3.680	5316	5084	3132	293	416	171
NM_153118	Fnbp1l	2.322	1.717	4446	4951	3488	1354	1777	1095
NM_013538	Cdca3	2.265	2.454	4875	5905	3244	69	186	82
NM_146171	Ncapd2	2.257	1.640	4936	7006	4155	571	821	384
NM_011623	Top2a	2.245	2.572	4790	4673	3575	722	700	858
NM_023061	Mcam	2.240	1.206	4445	3741	893	844	298	685
NM_001033302	Gm129	2.210	2.090	1924	2468	1563	532	108	45

NM_023210	Anp32e	2.202	1.200	4601	4682	3089	312	238	183
NM_024245	Kif23	2.195	1.999	3901	2993	3907	380	457	811
NM_013746	Plekhb1	2.185	1.398	297	214	110	818	253	208
NM_009834	Ccrn4l	2.152	1.978	5292	6368	3262	340	440	177
NM_001033244	Fancd2	2.131	2.883	3362	5296	3268	1167	1983	1512
NM_011121	Plk1	2.120	2.550	4246	4172	3050	287	547	183
NM_007598	Cap1	2.089	1.469	3497	5721	3099	563	543	384
NM_010931	Uhrf1	2.055	2.099	5164	5861	4560	506	600	290
NM_145588	Kif22	2.050	2.447	4878	3468	2283	326	483	188
NM_012011	Eif2s3y	2.004	2.240	476	1965	111	128	233	176
NM_008142	Gnb1	2.001	1.584	151	221	159	420	648	346
NM_027290	Mcm10	2.000	3.741	2065	1228	1384	524	426	513
NM_001039556	E130016E03Rik	1.995	2.967	1136	1920	1256	893	858	727
NM_010937	Nras	1.975	2.219	1353	2981	1354	126	227	77
NM_011497	Aurka	1.941	2.685	6381	5560	5773	271	468	293
NM_172301	Ccnb1	1.938	3.138	2807	3166	2233	184	195	307
NM_010415	Hbegf	1.904	1.770	3090	5358	3684	558	336	384
NM_172746	Hirip3	1.895	2.630	2528	3220	2188	81	29	46
NM_013928	Schip1	1.888	1.654	1703	2231	743	1231	1167	673
NM_007792	Csrp2	1.881	2.162	2995	6150	3359	558	583	580
NM_029850	Bcl7a	1.878	1.436	4825	5424	3348	1105	804	332
NM_028355	Tmem48	1.877	1.632	4080	3460	2203	593	894	457
NM_025520	Lsm5	1.870	2.260	4164	4349	2864	55	123	143
NM_134122	Nrm	1.829	1.491	2393	2223	1973	234	181	78
NM_016750	H2afz	1.816	2.826	5420	3703	2266	94	54	43
NM_008126	Gjb3	1.813	4.787	2455	316	128	134	192	120
NM_009689	Birc5	1.812	2.647	4960	4010	3872	181	311	155
NM_145409	Chtf18	1.810	2.158	4048	3028	4594	354	321	184
NM_001039090	Skil	1.805	1.582	5004	5274	3001	593	486	253

NM_009015	Rad54l	1.803	2.361	2663	2734	1377	382	387	255
NM_009412	Tpd52	1.786	2.956	8053	4165	3341	1380	1980	713
NM_001042527	Blm	1.764	3.083	3829	3705	2483	1122	1406	993
NM_138744	Ssx2ip	1.755	2.080	2820	2925	2011	777	889	570
NM_010220	Fkbp5	1.740	2.254	5356	4841	3482	1723	3039	2366
NM_021273	Ckb	1.737	1.926	6489	1542	1073	659	178	680
NR_002885	LOC654467	1.735	2.598	6633	5814	3780	205	155	105
NM_008564	Mcm2	1.734	2.057	3935	2796	2222	423	457	256
NM_011522	Syngr3	1.715	1.925	2660	1216	499	723	1044	1548
NM_028039	Esco2	1.712	3.283	4851	2096	2524	178	493	367
NM_011638	Tfrc	1.707	1.601	3192	3394	3818	395	495	637
NM_028958	Taf7l	1.688	2.670	353	127	125	1033	200	318
NM_172714	Lin54	1.683	1.133	3371	4295	3029	910	959	591
NM_025411	1110049F12Rik	1.673	1.277	4412	3880	2822	258	250	62
NM_026163	Pkp2	1.661	2.357	3109	3203	2531	1624	1426	1641
NM_133939	Lsm8	1.651	1.568	1206	3554	1973	146	177	106
NM_011132	Pole	1.641	1.326	3940	2879	2271	865	1285	674
NM_010615	Kif11	1.638	1.894	4813	5826	4799	822	729	1103
NM_181595	Ppp1r9a	1.619	1.900	4778	5128	2632	6594	12897	25151
NM_021516	Mark3	1.618	1.395	5572	6380	4784	1836	1696	1066
NM_028030	Rbpms2	1.616	4.066	6572	3889	3318	529	584	706
NM_028128	Rfc5	1.614	2.743	3230	1570	1182	305	342	97
NM_009769	Klf5	1.600	6.519	6125	2170	1427	427	215	1253
NM_197959	3000004C01Rik	1.599	2.136	4838	4187	3441	646	397	379
NM_145508	Dyrk3	1.594	4.227	3549	2299	2711	266	208	424
NM_001014976	Espl1	1.590	2.112	2407	2658	2438	587	965	588
NM_028109	Tpx2	1.579	1.583	2497	2788	2342	717	691	1051
NM_179203	Atad3a	1.578	1.926	4008	4468	3379	559	414	182
NM_010721	Lmnb1	1.565	3.869	10613	6722	8357	864	1131	927

NM_025840	Bzw2	1.564	2.462	5090	6826	4755	929	968	884
NM_021891	Fignl1	1.561	3.027	2599	2319	2532	219	153	298
NM_028002	Dus4l	1.558	1.516	4779	3527	2656	274	338	267
NM_172578	C79407	1.550	1.958	3170	2505	1662	558	845	493
NM_178185	Hist1h2ao	1.548	1.846	54	50	76	10	10	10
NM_011304	Ruvbl2	1.545	3.132	5294	4487	3415	376	322	95
NM_008815	Etv4	1.535	2.684	3522	2597	2201	1348	320	543
NM_001025192	Cxadr	1.524	1.474	4068	2304	2907	1423	1093	1754
NM_172722	C330023M02Rik	1.518	2.048	4442	4218	2780	754	789	410
NM_028151	Skiv2l2	1.518	2.519	4426	4997	4521	1046	1260	1540
NM_172468	Snx30	1.516	1.205	6608	6671	4364	2220	2125	1285
NM_019939	Mpp6	1.513	1.382	2661	4433	3502	1735	2742	2279
NM_011401	Slc2a3	1.510	5.616	10130	2120	782	417	963	753
NM_178183	Hist1h2ak	1.508	4.799	328	759	501	40	42	12
NM_009791	Aspm	1.497	1.090	4479	3634	3811	725	608	1056
NM_011565	Tead2	1.497	1.858	1726	2392	1683	503	536	133
NM_001008421	Nol10	1.491	2.693	3476	4771	2585	1689	2219	1378
NM_001077596	Shroom3	1.489	1.049	5526	3626	1492	2261	2080	1241
NM_025796	Mrpl33	1.486	1.024	1853	2786	1045	164	253	94
NM_027539	Dclk2	1.471	2.583	6707	6210	2750	3654	2462	2590
NM_016957	Hmgn2	1.465	1.185	3769	2628	2107	53	101	22
NM_011710	Wars	1.454	1.467	2163	3829	1777	709	955	375
NM_173400	6230416J20Rik	1.449	2.115	4242	3873	2439	449	503	331
NM_022979	Nup98	1.443	1.971	4210	4437	3334	1032	939	893
NM_012006	Acot1	1.441	3.003	949	840	377	686	104	426
NM_026014	Cdt1	1.432	2.697	5330	3160	3856	235	114	70
NM_010066	Dnmt1	1.430	1.053	4443	3351	4133	961	1011	1039
NM_198605	F630043A04Rik	1.429	2.115	5497	3432	3871	466	789	505
NM_016777	Nasp	1.429	3.515	5329	3776	2465	206	549	373

NM_139117	Csda	1.416	1.980	4200	6153	4463	698	853	682
NM_053074	Nup62	1.411	2.377	6781	8132	3380	294	199	135
NM_009125	Atxn2	1.402	1.146	6288	6951	4746	1726	2144	1125
NM_028120	Ccdc123	1.401	1.158	2448	2569	1927	998	578	374
NM_146154	Ppp1r8	1.397	1.919	4324	4094	2956	387	326	157
NM_008252	Hmgb2	1.392	4.492	3458	3220	2372	101	104	39
NM_001081212	Irs2	1.387	1.654	8988	18482	5099	1374	775	1174
NM_144818	Ncaph	1.385	1.630	2956	2489	2750	536	323	389
NM_027460	Slc25a33	1.383	1.942	2896	2920	2332	778	543	329
NM_009104	Rrm2	1.382	2.002	4938	4286	2839	188	191	95
NM_016692	Incenp	1.378	1.850	4352	4578	4360	570	645	568
NM_001045807	Rbm15	1.377	1.764	8787	7008	4861	210	203	161
NM_011075	Abcb1b	1.367	2.168	1942	1591	1272	1919	1071	770
NM_029094	Pik3cb	1.364	2.762	4640	3372	3294	1450	2011	2312
NM_178113	Ncapd3	1.352	1.296	6768	6077	6277	889	1437	1344
NM_013733	Chaf1a	1.346	1.616	2604	3508	2560	455	747	668
NM_178184	Hist1h2an	1.343	2.072	231	428	325	25	16	18
NM_177663	Isg20l2	1.341	1.722	4574	4178	3108	176	348	131
NM_016690	Hnrpdl	1.341	2.377	8542	9087	5297	119	92	44
NM_019693	Bat1a	1.340	1.412	6710	8594	8395	337	332	268
NM_172584	Itpk1	1.329	2.647	6588	4131	3116	3759	4053	2264
NM_028131	Cenpn	1.329	2.492	4756	2999	3672	503	584	581
NM_025866	Cdca7	1.325	4.100	4971	2872	3626	295	246	192
NM_145946	Fanci	1.325	2.355	3810	2380	2096	939	1138	659
NM_019660	Mycbp	1.322	1.850	2201	2669	1996	274	160	116
NM_017397	Ddx20	1.322	2.105	4397	3286	2616	281	72	168
NM_001033201	AU014645	1.321	1.460	5196	7469	4021	663	742	440
NM_175661	Hist1h2af	1.319	2.068	326	739	623	30	10	17
NM_145822	Cd3eap	1.318	1.651	2950	2890	2078	113	86	30

NM_025814	Serbp1	1.314	1.690	8560	12182	7941	473	665	495
NM_021714	Wbp11	1.312	1.401	4443	5071	3880	215	459	289
NM_011119	Pa2g4	1.310	1.924	7972	6536	5858	229	383	225
NM_021512	Nup160	1.309	1.255	5144	4187	4077	823	930	1147
NM_175265	6720463M24Rik	1.307	2.143	4696	4002	4519	568	506	590
NM_148917	Pabpc4	1.305	2.558	1558	2180	1920	243	354	191
NM_207238	Fbxo27	1.302	2.966	3196	775	241	132	719	719
NM_010219	Fkbp4	1.300	1.849	6927	7084	6029	268	402	156
NM_026993	Ddah1	1.287	1.204	2927	3615	2596	4742	3466	1610
NM_023536	Mrto4	1.280	2.574	5771	5187	3352	146	131	84
NM_010414	Htt	1.279	1.107	5058	6149	3767	2264	3135	1626
NM_009773	Bub1b	1.278	2.798	2410	3306	3110	824	712	881
NM_016926	Sart3	1.276	1.324	4708	4128	2905	471	820	283
NM_027494	Zcchc8	1.270	1.165	3981	4537	3208	494	471	275
NM_013807	Plk3	1.269	3.416	5701	4191	2887	228	168	81
NM_010790	Melk	1.269	1.901	2823	2407	1616	992	1018	616
NM_170779	Wwc1	1.268	2.473	6472	3050	2709	4885	6362	10372
NM_010353	Gsg2	1.265	2.002	4207	4517	3967	183	68	95
NM_001039129	Hnrpa1	1.265	3.097	6643	5854	3789	206	163	120
NM_029797	Mnd1	1.264	2.943	1096	1178	1251	1237	1331	901
NM_016682	Uba2	1.257	1.576	4788	3398	2768	513	622	234
NM_026352	Ppid	1.256	1.735	3926	4815	2683	223	177	342
NM_011131	Pold1	1.255	2.763	2451	1676	1712	660	452	202
NM_176933	Dusp4	1.255	1.280	9722	14104	8879	1179	186	113
NM_153396	Mical3	1.255	2.212	1077	1295	770	2191	2921	1471
NM_011258	Rfc1	1.254	1.817	3021	3642	2463	1241	1229	652
NM_029157	Sf3a3	1.245	1.514	3416	2622	2357	273	482	152
NM_023525	Cad	1.228	1.909	4381	3879	2549	552	664	303
NM_008949	Psmc3ip	1.227	2.836	3102	1617	2123	75	81	100

NM_027148	Exosc8	1.226	1.152	6726	5948	5072	155	149	77
NM_026849	Mtmr14	1.219	1.366	2463	4498	3338	917	1474	941
NM_025642	2610039C10Rik	1.217	1.242	3009	2601	3889	222	406	242
NM_027427	Taf15	1.215	2.923	4900	6001	4241	671	594	979
NM_175175	Plekhf2	1.205	4.863	7314	4200	2970	262	555	285
NM_009261	Strbp	1.204	2.765	172	274	388	1274	1510	3214
NM_023203	2410015N17Rik	1.196	2.984	2647	1145	1036	89	70	45
NM_025626	3110001A13Rik	1.196	1.357	3693	4470	3717	2986	1079	1741
NM_018868	Nol5	1.194	1.639	5328	3989	3115	488	479	766
NM_172697	Prpf38a	1.193	1.330	5419	3745	2589	231	210	182
NM_021895	Actn4	1.190	1.868	7375	6366	4351	1534	1558	705
NM_009097	Rps6ka1	1.186	3.406	4641	2907	2236	970	1234	493
NM_199196	Suz12	1.185	2.879	5626	4622	4833	747	762	1084
NM_001013026	Ttf2	1.184	1.920	2530	1831	1634	472	735	284
NM_013827	Mtf2	1.177	3.192	4631	2741	2027	528	940	601
NM_016699	Exosc10	1.176	1.377	2632	2929	1695	589	472	312
NM_019830	Hrmt1l2	1.174	2.332	5181	3991	3088	214	301	89
NM_020587	Sfrs4	1.173	1.355	5847	4654	3347	625	455	285
NM_019748	Sae1	1.173	1.286	3949	2823	2482	888	1171	613
NM_008566	Mcm5	1.170	2.901	6501	2524	3995	336	354	365
NM_009056	Rfx2	1.165	4.068	2734	1987	1590	1478	3584	3292
NM_145964	BC002199	1.162	1.014	2673	3216	2151	439	574	278
NM_026231	Gstcd	1.162	1.440	4849	5825	3794	1397	1519	1145
NM_001080979	Tead4	1.162	1.129	5445	7823	6166	2766	2113	1273
NM_011239	Ranbp1	1.157	2.032	7416	6061	8373	201	333	211
NM_133878	Rcc1	1.148	1.564	3958	2960	2593	230	235	161
NM_145923	Rell1	1.146	1.586	4116	4011	2890	1254	1376	776
NM_144805	Tmem40	1.142	2.594	4162	525	369	908	1506	1288
NM_030597	Lsm2	1.137	2.512	4596	4692	3758	78	104	122

NM_023215	2500003M10Rik	1.137	1.577	3260	3894	2135	138	229	122
NM_026714	0610037D15Rik	1.135	1.095	1162	1800	995	88	30	42
NM_145393	Ythdf2	1.135	1.470	4146	5048	3426	313	558	265
NM_178683	Depdc1b	1.126	1.894	5078	2801	4074	1511	1642	1800
NM_013604	Mtx1	1.123	2.600	4354	3611	2621	189	144	80
NM_021284	Kras	1.119	1.204	5463	6589	5143	640	982	789
NM_025639	Cenpm	1.115	2.694	3431	2800	2722	272	227	233
NM_172681	D930015E06Rik	1.109	1.298	6184	4860	4093	3524	2500	1854
NM_053202	Foxp1	1.106	1.032	6119	15009	11044	8537	7275	5560
NM_025654	Rdm1	1.104	3.932	1160	915	374	242	240	239
NM_001025365	D4Wsu114e	1.104	1.685	3784	4559	2647	214	304	140
NM_026079	Ikbkap	1.104	1.154	6700	5474	3856	935	818	680
NM_027901	Gtf3c2	1.102	1.088	5630	4725	3552	448	684	215
NM_153566	Yrdc	1.092	1.818	6219	5263	4547	121	121	57
NM_009765	Brca2	1.092	1.466	2684	2501	2191	629	831	1203
NM_144553	Dlgap5	1.090	1.947	1673	2521	1903	681	427	741
NR_002898	Snora65	1.089	2.943	3776	2334	2616	23	26	43
NM_177150	Cenpt	1.087	2.106	3590	2091	2758	246	196	110
NM_009477	Upp1	1.085	5.483	5345	1817	1445	679	509	1444
NM_013896	Timm9	1.085	1.043	3031	3610	2403	202	282	306
NM_172665	Pdk1	1.077	2.778	3685	2817	2749	581	404	1767
NM_030116	Mrpl9	1.072	1.482	3951	4096	3032	80	132	78
NM_133756	Gpn1	1.071	2.919	2748	2350	1500	401	319	168
NM_019800	Acp6	1.070	2.126	4356	3362	2706	358	380	289
NM_148948	Dicer1	1.069	2.371	3541	3938	3358	1616	1454	981
NM_145958	Kbtbd2	1.064	1.215	3733	9380	5919	360	627	522
NM_011241	Rangap1	1.064	1.789	6405	5428	5032	530	598	753
NM_026374	Ilf2	1.064	1.350	2791	2496	1754	135	108	137
NM_199447	Rrp12	1.063	2.232	3676	3204	3743	940	608	558

NM_001042620	Dhx15	1.061	1.733	3625	6411	3490	526	643	610
NM_026872	Ubap2	1.060	1.477	5457	3878	3104	1226	1604	1047
NM_133740	Prmt3	1.059	1.158	1867	1954	1311	1235	1596	1288
NM_017374	Ppp2cb	1.056	1.582	3287	3057	2165	369	238	195
NM_001081392	Mdn1	1.052	2.463	3364	4030	2395	1793	2267	1701
NM_033526	Ubqln4	1.050	2.191	7895	3811	3162	371	409	117
NM_175224	Metap1	1.047	1.019	2798	4231	2468	449	740	324
NM_019671	Net1	1.047	1.122	3844	5501	4709	906	770	911
NM_011959	Orc5l	1.044	1.390	4214	4086	2157	709	917	1007
NM_025729	Map3k7ip3	1.043	1.382	1859	2176	2531	420	709	736
NM_001080127	Rnps1	1.042	1.255	6860	8115	6190	212	446	307
NM_199467	F730047E07Rik	1.039	1.369	361	493	469	1244	1841	1436
NM_028244	Rrp1b	1.038	3.222	5712	4774	5128	835	985	555
NM_001004364	Ddef2	1.036	1.421	3703	2695	2285	4438	3763	2146
NM_029508	Pcgf5	1.033	2.210	2077	2566	2415	2474	2437	2933
NM_011358	Sfrs2	1.032	1.578	11591	8889	9211	214	175	61
NM_026309	Lsm3	1.031	1.350	4940	4487	4179	246	370	192
NM_207205	Igsf3	1.026	2.385	3353	4411	2164	2546	2086	1027
NM_133872	Aof2	1.025	1.643	3311	4548	2890	743	1130	884
NM_199009	Fam160a2	1.023	1.739	3599	1920	2620	339	415	208
NR_001592	H19	1.023	2.525	104	35	60	492	226	729
NM_139061	Vps54	1.022	1.332	4882	4450	4678	1753	1966	2100
NM_057172	Fubp1	1.021	1.845	8173	9872	5149	381	323	273
NM_010715	Lig1	1.012	2.484	2226	1642	1652	527	705	417
NM_009951	Igf2bp1	1.010	5.464	13046	2828	1122	1279	5483	6832
NM_173189	Mcph1	1.008	1.434	5474	4843	6764	4475	4175	2920
NM_145552	Gnl2	1.008	1.517	3872	4079	2234	522	631	346
