# Supplementary material for: Identification of the early and late responder genes during the generation of induced pluripotent stem cells from mouse fibroblasts
Source: PLoS One. 2017 Feb 2;12(2):e0171300. doi: 10.1371/journal.pone.0171300 (PMC5289558; doi:10.1371/journal.pone.0171300)
Supplement: S3 Table — (PDF) [file pone.0171300.s009.pdf]

S3 Table. Resistant up genes

Gene information		mRNA fold change		H3K4me3 enrichment			H3K27me3 enrichment		
RefSeq gene	gene symbol	iPSCp/sFB-G	mESC/sFB-G	mESC	iPSCp	sFB-G	mESC	iPSCp	sFB-G
NM_025274	Dppa5	-0.288	8.542	422	10	11	10	55	27
NM_011934	Esrrb	-0.330	7.226	18927	1666	1943	11018	13562	6321
NM_212457	Bex4	0.625	6.867	325	14	36	10	85	163
NM_026480	2410146L05Rik	-0.114	6.789	1161	21	164	48	230	639
NM_013633	Pou5f1	0.093	6.689	9556	49	95	199	784	330
NM_001081202	L1td1	0.217	6.418	7853	95	64	163	719	379
NM_026323	Wfdc2	-0.040	6.388	776	179	517	303	937	1450
NM_028602	Tex19.1	-0.006	6.344	1392	37	47	229	141	75
NM_015798	Fbxo15	-0.345	6.100	6258	3897	3605	734	1826	2501
NM_009434	Phlda2	0.090	6.026	1938	166	398	429	186	540
NM_139218	Dppa3	-0.022	5.918	1805	233	105	217	345	145
NM_009903	Cldn4	-0.193	5.901	9656	65	77	123	200	159
NM_016754	Mylpf	0.412	5.876	1053	75	201	45	109	29
NM_001005423	Mreg	0.840	5.843	5963	1972	1934	1215	1970	4531
NM_011107	Pla2g1b	0.125	5.837	381	10	103	237	338	107
NM_010174	Fabp3	-0.540	5.820	6445	544	978	153	876	554
NM_175503	Aard	0.095	5.774	2348	54	120	445	403	481
NM_009335	Tcfap2c	-0.093	5.706	8729	31	307	1320	607	3290
NM_144953	1700019D03Rik	0.034	5.552	4120	538	667	470	1082	2238
NM_175303	Sall4	0.103	5.524	9282	108	444	308	1503	3910
NM_009426	Trh	-0.165	5.417	964	32	65	574	1140	1077
NM_022409	Zfp296	-0.123	5.300	4641	406	669	97	75	106
NM_023894	Rhox9	0.010	5.096	62	10	20	10	10	10
NM_018815	Nup210	0.954	5.004	4143	2732	1799	2894	19779	8416
NM_016968	Olig1	0.317	4.979	6065	179	170	253	1104	1566

NM_008342	Igfbp2	-0.209	4.976	4122	479	689	1102	3871	3379
NM_009482	Utf1	-0.107	4.914	6972	31	239	182	565	1069
NM_008698	Nipsnap1	0.246	4.834	2836	600	911	710	1731	1855
NM_145438	Llgl2	-0.920	4.797	4916	985	3220	1589	2655	995
NM_175332	E130012A19Rik	0.121	4.694	5889	517	1491	301	729	1709
NM_010346	Grb7	-0.114	4.579	1034	109	142	575	1936	1672
NM_018777	Cldn6	-0.093	4.540	1303	148	78	634	851	1314
NM_009575	Zic3	0.055	4.513	7267	471	230	127	630	1704
NM_027941	Lrrc34	0.168	4.398	856	92	109	424	606	557
NM_013721	Rpl7a	0.110	4.395	8648	5784	6540	71	91	49
NM_013873	Sult4a1	-0.374	4.363	3431	337	622	1656	1976	2812
NM_011987	Pla2g10	-0.057	4.340	448	60	126	319	1115	937
NM_212483	Krt42	0.007	4.318	286	147	118	677	448	392
NM_008652	Mybl2	0.356	4.209	15311	1946	2416	654	451	423
NM_019448	Dnmt3l	-1.428	4.189	3018	171	693	322	1145	503
NM_175329	Chchd10	-2.545	4.188	4062	57	511	146	148	80
NM_198412	Dnajc6	0.192	4.126	1022	767	485	2290	3702	3435
NM_021480	Tdh	-0.010	4.106	6449	184	184	461	1986	2662
NM_133982	Rpp25	-0.598	4.082	3673	361	692	126	517	723
NM_013914	Snai3	-0.106	4.081	1494	41	197	374	944	1247
NM_029821	1190003J15Rik	0.075	4.045	985	10	28	168	164	51
NM_001033425	Zscan10	-0.188	3.994	3748	263	218	313	1675	587
NM_025285	Stmn2	0.919	3.961	2621	488	614	1141	1941	1473
NM_011443	Sox2	0.803	3.956	10885	5892	122	170	386	1172
NM_011861	Pacsin1	0.002	3.939	3367	1003	1324	3865	10630	5409
NM_153577	AI428936	0.126	3.928	1389	51	33	91	121	46
NM_011618	Tnnt1	0.124	3.927	271	80	57	441	698	161
NM_181548	Eras	0.043	3.920	1787	45	11	189	325	518
NM_016697	Gpc3	-0.240	3.910	2809	1564	1679	5439	7599	11767

NM_080575	Acss1	0.732	3.841	2971	803	1538	2124	2023	2545
NM_178922	Hic2	0.154	3.708	7473	4188	4928	582	945	668
NM_009328	Tcf15	-0.002	3.706	3481	39	413	891	707	2239
NM_021391	Ppp1r1a	0.145	3.683	2996	294	596	600	1905	1819
NM_026631	Nola2	0.202	3.681	4593	3099	3069	91	100	115
NM_144841	Otx2	-0.104	3.667	1940	100	62	1703	1308	1942
NM_009556	Zfp42	0.212	3.632	2478	83	30	116	282	175
NM_010202	Fgf4	0.045	3.612	10109	49	53	115	2050	1512
NM_015782	Snrpa	0.365	3.592	3867	3563	2280	183	288	89
NM_028060	Slc35f2	0.085	3.585	4551	167	773	875	2149	1939
NM_011677	Ung	0.297	3.574	5314	2111	1740	286	165	89
NM_178254	Tcfl5	-0.236	3.552	3848	110	239	696	1367	2549
NM_177776	Smtnl2	-0.003	3.537	4602	521	1166	1533	2857	3666
NM_172670	Gyltl1b	0.006	3.537	2449	14	225	256	470	315
NM_009337	Tcl1	0.055	3.530	1105	77	22	307	249	136
NM_028913	Zfp819	-0.158	3.512	2263	17	188	222	800	361
NM_030677	Gpx2	0.126	3.490	180	32	23	242	310	122
NM_010357	Gsta4	-0.319	3.487	1346	308	507	233	644	637
NM_027366	Ly6g6e	-0.053	3.472	710	148	223	149	488	354
NM_013659	Sema4b	0.777	3.437	9975	5398	4280	878	997	614
NM_153547	Gnl3	-0.158	3.422	4939	5150	4394	89	155	173
NM_008635	Mtap7	-0.003	3.397	5975	1947	2597	4037	10082	10593
NM_145222	B3gnt7	0.186	3.344	6319	79	527	175	1090	1384
NM_176834	Rnf208	0.009	3.340	1217	19	262	212	290	847
NM_013474	Apoa2	-0.052	3.320	10	11	20	122	73	48
NM_144819	Ccdc92	0.934	3.317	5359	3445	3050	633	1911	1607
NM_008955	Rhox6	-0.115	3.316	121	31	17	195	85	20
NM_009864	Cdh1	-0.093	3.308	8166	723	1471	1441	4222	7319
NM_009944	Cox7a1	0.802	3.299	144	233	112	106	94	55

NM_009749	Bex2	0.585	3.266	401	237	149	47	75	179
NM_028715	Fcho1	-0.062	3.250	1691	98	453	1634	1364	1765
NM_013611	Nodal	0.048	3.247	7291	88	256	149	1951	1932
NM_011300	Rps7	0.369	3.246	6115	4775	2544	121	173	91
NM_016889	Insm1	0.134	3.245	5253	352	628	1447	1518	2434
NM_022004	Fxyd6	-2.319	3.245	1343	196	835	1514	1478	1575
NM_001033865	Rps27a	0.506	3.241	5729	4924	4250	110	58	31
NM_016689	Aqp3	0.008	3.223	2086	124	185	999	721	469
NM_011600	Tle4	0.900	3.218	6266	4845	3686	2786	4588	5091
NM_008506	Mycl1	0.795	3.206	4278	1627	1372	727	801	746
NM_019547	Rbm38	0.669	3.179	4310	2527	2527	512	410	353
NM_010071	Dok2	0.201	3.168	797	32	54	548	163	201
NM_007905	Phc1	0.397	3.148	2775	346	165	386	437	456
NM_008452	Klf2	-0.750	3.143	12638	2797	4893	133	240	33
NM_010311	Gnaz	0.237	3.134	3214	1131	1751	3795	4028	3221
NM_010663	Krt17	-0.035	3.115	307	72	83	478	232	283
NM_009171	Shmt1	0.696	3.109	2345	1492	904	648	610	614
NM_009528	Wnt7b	0.057	3.103	6906	991	1481	8738	9093	6355
NM_023137	Ubd	0.109	3.101	157	86	12	84	90	259
NM_008860	Prkcz	-0.335	3.050	5765	1577	1998	3749	11787	4213
NM_031386	Tex14	-0.039	3.041	3875	2646	2125	3857	8055	5981
NM_019689	Arid3b	0.027	3.040	733	242	463	1150	1662	1950
NM_146177	Suv420h2	0.209	3.032	1924	2430	1897	221	340	83
NM_026115	Hat1	0.993	3.007	4228	2011	3235	924	852	1119
NM_033608	Igsf9	0.587	3.001	1268	606	848	1216	1081	1650
NM_007431	Akp2	-0.081	2.995	3536	588	452	1533	6727	1946
NM_010900	Nfatc2ip	0.585	2.963	4571	2502	2344	240	275	135
NM_031170	Krt8	0.014	2.962	405	135	155	890	1325	783
NM_011328	Sct	-0.126	2.958	888	32	146	198	476	157

NM_010264	Nr6a1	0.135	2.957	5368	7966	8578	4974	3737	4889
NM_025554	Polr2e	0.199	2.956	3536	3539	3176	167	239	104
NM_153404	Liph	0.253	2.939	836	599	755	971	1485	1056
NM_019535	Sh3gl2	0.051	2.921	7453	1197	1342	4808	7260	6801
NM_001080969	Thg1l	0.329	2.918	1636	829	1282	252	126	330
NM_177742	Triml1	0.198	2.913	55	66	39	93	160	184
NM_028083	Chaf1b	0.986	2.909	3525	1811	3302	427	715	260
NM_009446	Tuba3a	0.052	2.902	119	66	90	769	926	215
NM_011289	Rpl27	-0.036	2.899	6319	4126	3378	153	38	67
NM_146257	Slc29a4	-0.235	2.893	3266	187	651	1624	2275	1970
NM_145464	Sox21	0.025	2.887	6830	288	658	1167	358	1641
NM_172963	1110012J17Rik	0.669	2.874	11636	4846	5849	3561	6206	4500
NR_003634	Rps4y2	-2.835	2.872	4059	235	1048	65	36	62
NM_016964	Stag3	-0.294	2.862	984	612	1070	1199	1873	1537
NM_029186	Tmem180	0.020	2.861	1505	330	1002	552	777	427
NM_020002	Rec8	-0.137	2.849	214	64	85	350	423	353
NM_177261	Kndc1	0.183	2.842	2264	518	460	3734	4252	3988
NM_144811	Cbx7	-0.069	2.820	4322	2121	2042	411	583	776
NM_021501	Pias4	0.258	2.818	2840	5983	3889	388	532	224
NM_011461	Spic	-0.087	2.792	101	68	48	319	558	518
NM_025290	Rsph1	-1.013	2.791	1083	552	1098	553	2981	1258
NM_016672	Ddc	-0.766	2.790	1295	644	764	3434	3264	3572
NM_175342	Cphx	0.052	2.763	10	10	10	10	10	10
NM_010139	Epha2	0.775	2.755	9057	4561	5042	775	711	415
NM_175433	Zfp710	0.741	2.743	6592	6118	4038	1351	1344	646
NM_173370	Cds1	0.097	2.742	3421	1493	1217	2548	6216	2405
NM_021356	Gab1	0.526	2.739	10158	10081	9817	3058	2245	2211
NM_019812	Sirt1	0.479	2.723	5062	3810	3373	499	426	577
NM_009366	Tsc22d1	0.422	2.712	7220	6235	5216	211	133	107

NM_001080945	Nanogpd	-0.032	2.699	6773	78	38	127	376	214
NM_194055	Rbm35a	0.021	2.692	3931	439	484	2176	5354	4152
NM_026967	Rhebl1	0.668	2.690	2665	2293	2477	128	305	87
NM_010830	Msh6	0.584	2.687	6433	4210	4424	382	485	418
NM_009224	Snrp70	0.670	2.663	3907	4232	2726	317	256	255
NM_012054	Aoah	0.107	2.659	1158	1393	1549	7103	6692	9018
NM_011898	Spry4	0.123	2.643	10659	5835	5826	971	416	750
NM_148942	Serpinb6c	-0.160	2.639	286	140	156	392	1019	637
NM_009173	Siah1b	0.860	2.634	2178	1278	1663	56	10	37
NM_009897	Ckmt1	-0.643	2.624	658	58	185	386	1529	1614
NM_011597	Tjp2	0.588	2.623	9162	5624	4975	2764	4137	3796
NM_144898	Msto1	0.546	2.622	4096	3200	2582	131	132	63
NM_178394	Jakmip1	-0.002	2.621	2564	606	554	3636	4852	3398
NM_008568	Mcm7	0.641	2.619	7046	4933	4800	191	140	103
NM_001033954	Calca	0.352	2.617	528	324	103	512	319	571
NM_175934	Ppp1r10	0.852	2.611	4483	10255	4119	435	595	380
NM_001077705	Ptpn6	0.804	2.610	632	499	515	619	1134	531
NM_007633	Ccne1	0.240	2.604	3875	3213	2752	276	174	94
NM_144800	Mtss1	-0.463	2.596	8655	2592	6857	4006	12789	3899
NM_145833	Lin28	-0.045	2.591	4688	206	97	353	3700	1784
NM_145973	Ell3	-0.149	2.589	1622	151	460	134	277	140
NM_138586	Exosc5	0.381	2.589	3298	2732	1696	291	255	88
NM_013872	Pmm1	-0.534	2.588	7625	3408	3947	366	174	233
NM_030246	Wdr21	-0.310	2.584	289	111	75	286	279	258
NM_007658	Cdc25a	0.351	2.584	5332	4386	4793	265	242	367
NM_170669	Rps15a	0.462	2.582	7230	6619	3408	111	192	122
NM_010664	Krt18	-0.089	2.577	618	64	144	1004	1137	667
NM_178734	Zfp473	0.028	2.573	2894	2492	1763	245	273	186
NM_001004154	Rragb	0.316	2.551	1595	614	522	534	796	840

NM_173383	Dnd1	-0.106	2.543	1806	30	26	225	30	65
NM_027470	Pak4	0.527	2.541	2775	2385	2249	881	818	480
NM_023794	Etv5	0.643	2.539	6646	3447	4100	1159	1534	1180
NM_011284	Rpa2	0.693	2.520	3730	3198	1975	163	138	101
NM_144912	Rad9b	0.121	2.497	3804	3688	2340	761	540	434
NM_008563	Mcm3	0.620	2.483	1495	2101	1878	365	380	477
NM_008430	Kcnk1	-0.156	2.476	3066	302	388	2001	1990	1719
NM_138745	Mthfd1	0.616	2.474	5650	2968	3235	1139	1421	804
NM_013882	Gtse1	0.724	2.468	5678	3964	4380	362	377	386
NM_175126	Zcchc3	-0.009	2.461	4486	2423	3721	134	162	204
NM_001002272	Tro	0.461	2.454	957	540	240	171	515	353
NM_175655	Hist1h4f	0.013	2.450	343	243	320	10	71	21
NM_175482	Usp28	0.258	2.449	4003	3418	3611	837	992	1218
NM_007634	Ccnf	0.907	2.448	4946	5903	3635	592	902	549
NM_021453	Pga5	0.029	2.448	251	82	42	615	1013	448
NM_013636	Ppp1cc	0.104	2.441	3738	5265	3067	269	522	181
NM_026410	Cdca5	0.691	2.440	3748	3692	3681	169	164	142
NM_007974	F2rl1	0.693	2.438	5431	1495	1336	382	815	552
NM_009721	Atp1b1	-1.373	2.438	6289	633	1723	648	830	2107
NM_178381	Tmem16j	-0.016	2.436	770	148	89	482	1633	764
NM_021517	Pdzk1	-0.005	2.425	229	233	193	1020	1836	670
NM_027491	Rragd	0.375	2.408	3666	2144	1661	1336	2113	2854
NM_008014	Ppm1g	0.852	2.398	8213	6265	4178	409	431	214
NM_029017	Mrpl47	0.790	2.380	6231	4474	4042	178	321	100
NM_134011	Tbrg4	0.971	2.375	8129	5475	5248	320	264	352
NM_028334	Nup37	0.568	2.374	3419	7011	4625	667	951	823
NM_025856	1700029G01Rik	0.084	2.374	1949	226	379	342	245	141
NM_026496	Grhl2	0.015	2.370	4477	1468	1296	6945	10673	11329
NM_011950	Mapk13	-0.534	2.363	2087	345	816	821	1820	1231

NM_010914	Nfyb	0.524	2.361	2512	4911	3159	248	544	377
NM_009574	Zic2	0.064	2.359	9176	218	2578	2282	2108	2516
NM_144855	Cbs	-0.218	2.352	3841	655	1565	1543	4186	1491
NM_018761	Ctnnal1	-1.988	2.351	6132	2029	3198	1040	4576	886
NM_009579	Slc30a1	0.159	2.348	7199	4113	6312	459	217	244
NM_009226	Snrpd1	0.249	2.347	2961	2720	2981	228	213	370
NM_177857	A930010I20Rik	0.080	2.341	962	272	412	775	838	392
NM_026506	Snrpg	0.484	2.339	3783	3406	3027	158	143	132
NM_007907	Eef2	-0.254	2.335	11747	8868	8447	1717	1193	620
NM_011535	Tbx3	0.042	2.333	14656	4311	5628	2572	192	471
NM_001081458	Ppp2r5c	0.690	2.324	3886	3541	3017	1749	2104	1038
NM_028455	Arhgap8	-0.069	2.324	1707	651	929	2278	3738	2485
NM_153287	Axud1	-0.501	2.323	6470	5096	9107	300	346	138
NM_178017	Hmgb2l1	0.141	2.312	2963	1847	2315	621	695	712
NM_029948	Pramef12	-0.086	2.293	500	115	149	266	326	159
NM_007959	Etsrp71	-0.089	2.292	244	36	37	383	1718	1268
NM_021329	Rangrf	0.039	2.292	494	329	556	70	107	76
NM_013753	X99384	-0.219	2.288	5948	2933	4541	2024	1589	1547
NM_009657	Aldoc	-1.013	2.286	229	93	105	171	10	101
NM_007607	Car4	-0.025	2.284	1398	106	194	1510	1814	1603
NM_019563	Cited4	0.153	2.274	2733	280	604	273	733	376
NM_028028	Zswim1	-0.196	2.274	4508	1764	1589	167	121	106
NM_021897	Trp53inp1	-0.443	2.271	4511	4798	3108	364	386	283
NM_023663	Ripk4	0.113	2.270	2499	246	364	1846	1731	2106
NM_133686	Qprt	-0.008	2.265	614	194	283	633	1735	302
NM_008817	Peg3	0.895	2.259	5097	1478	854	646	683	400
NM_173737	8430410A17Rik	0.484	2.253	4355	4791	4119	337	1014	515
NM_013768	Prmt5	0.440	2.252	2623	1939	1902	285	219	229
NM_010693	Lck	0.515	2.252	1580	1491	641	225	456	241

NM_133754	Fblim1	0.195	2.245	4854	2484	3145	828	566	175
NM_001033264	Gls2	0.294	2.244	4773	2975	2679	679	711	760
NM_009413	Tpd52l1	0.068	2.243	3017	1199	2450	2747	3429	4197
NM_009483	Utx	0.498	2.229	4752	4572	5092	875	1417	1661
NM_013716	G3bp1	0.208	2.220	7775	5373	6206	903	681	890
NM_009469	Ulk1	0.234	2.204	8999	4110	3908	828	769	257
NM_007958	Smarcad1	0.608	2.203	4920	5433	4093	811	1594	1324
NM_028385	Setd5	0.549	2.201	6509	12704	7201	974	2249	1739
NM_145478	Pim3	0.198	2.194	10280	7202	8753	287	308	123
NM_022655	Ireb2	0.581	2.193	3633	4438	4545	645	771	1242
NM_054052	B3gnt5	0.845	2.190	1731	316	476	473	660	1820
NM_022316	Smoc1	-0.855	2.187	3705	1413	1440	5698	10858	4928
NM_019489	Ppie	0.635	2.185	2945	2373	1406	319	316	196
NM_027263	Apitd1	0.844	2.183	2747	1229	1292	252	108	99
NM_010413	Hdac6	0.410	2.174	1439	846	1553	248	267	210
NM_138597	Atp5o	0.307	2.174	3604	3074	3488	135	259	148
NM_001018087	Ldoc1	0.083	2.169	390	12	26	147	50	66
NM_019984	Tgm1	-0.033	2.165	1044	130	364	838	608	666
NM_015731	Atp9a	0.005	2.162	5306	828	1466	3382	5145	3515
NM_144907	Sesn2	0.728	2.153	7741	7675	4547	256	193	185
NM_201364	BC055324	0.735	2.152	3828	3215	3844	601	827	1156
NM_009235	Sox15	-0.053	2.151	3527	43	136	54	48	59
NM_028064	Slc39a4	-1.365	2.150	1023	109	515	313	790	154
NM_172303	Phf17	0.353	2.145	5904	5832	4208	1080	1205	478
NM_029341	Capsl	-0.021	2.139	266	298	128	521	1485	1766
NM_198645	Ccdc58	0.447	2.138	5565	4615	4933	573	523	560
NM_020271	Pdxp	0.384	2.137	2911	946	747	294	430	334
NM_001003918	Usp7	0.258	2.132	5647	4313	5797	872	984	1216
NM_011436	Sorl1	-0.144	2.130	4148	1200	2437	5111	10390	15989

NM_177640	D030056L22Rik	0.710	2.125	4237	3019	3422	108	178	89
NM_025918	Ccdc43	0.978	2.124	4869	2845	3391	373	283	433
NM_013932	Ddx25	-0.748	2.123	1415	1187	1818	1805	2075	1863
NM_021463	Prps1	0.871	2.108	2846	1911	2452	183	356	224
NM_146201	Zfp553	0.177	2.103	9944	4163	3307	136	104	41
NM_146100	Ina	-0.038	2.102	5699	227	743	1267	1813	2900
NM_001081413	Unc13b	0.462	2.098	3739	2150	2008	6411	10631	7489
NM_031165	Hspa8	-0.739	2.098	5933	3590	4478	169	121	20
NM_182995	6330503K22Rik	0.818	2.097	2717	1791	1897	517	277	272
NM_029045	4930432K21Rik	0.397	2.097	1680	2933	3775	877	595	1260
NM_010193	Fem1b	0.777	2.097	9515	7378	7950	255	548	471
NM_173382	2810046L04Rik	0.976	2.094	3829	5098	3412	543	456	133
NM_134097	Topors	0.846	2.092	9426	7285	5229	200	227	209
NM_007415	Parp1	0.451	2.088	3753	2561	4018	808	733	671
NM_001033408	Gm817	-0.055	2.087	492	95	181	768	1614	2265
NM_023331	Mrpl46	0.515	2.087	4836	3694	2676	160	193	96
NM_011588	Trim28	0.924	2.086	8149	5593	4285	306	182	110
NM_026091	1700037H04Rik	-0.012	2.084	1892	3601	4146	484	172	299
NM_008009	Fgfbp1	-0.097	2.084	61	27	14	180	194	120
NM_027521	Hmha1	0.502	2.076	742	326	342	671	714	327
NM_175287	A430005L14Rik	0.460	2.073	3058	2747	2071	119	291	25
NM_015818	Hs6st1	0.200	2.068	14807	4243	5261	1140	875	773
NM_024428	Dpy30	0.637	2.066	4959	4850	4247	509	817	587
NM_053261	Impa2	-0.341	2.065	7258	3271	4288	809	813	716
NM_138595	Gldc	-4.041	2.063	3884	1043	2583	1525	8019	2891
NM_009678	Ap1m2	-0.049	2.061	1615	128	112	575	902	790
NM_025641	Uqcrh	0.208	2.057	3759	5395	3448	150	168	112
NM_027106	Avpi1	0.326	2.057	4355	2898	3277	272	127	136
NM_133692	Pold3	0.873	2.057	2863	3049	2165	603	865	416

NM_026391	Ppp2r2d	0.720	2.054	6839	4636	4207	558	594	492
NM_008504	Gzmm	0.452	2.048	106	86	81	267	87	125
NM_146083	Sfrs7	0.692	2.046	7044	6732	5818	161	352	212
NM_009471	Umps	0.277	2.046	3776	3155	3578	348	181	445
NM_029747	2410137M14Rik	-0.022	2.042	680	101	93	124	1234	634
NM_010043	Des	0.115	2.029	1321	362	233	859	527	1377
NM_025394	Tomm7	0.322	2.024	1056	3154	1520	71	169	69
NM_025281	Lyar	0.991	2.024	5255	3539	3180	399	374	182
NM_011978	Slc27a2	0.424	2.022	4596	194	496	1429	4106	5099
NM_172826	Dact2	-0.050	2.021	4815	774	2340	667	2243	1037
NM_011420	Smn1	0.850	2.017	4520	3189	3259	230	462	267
NM_028115	Trub1	0.239	2.012	2507	1861	2473	838	902	906
NM_024216	Gpn3	0.743	2.008	4394	2837	2389	225	221	133
NM_011561	Tdg	0.668	2.001	2969	4465	3004	420	457	377
NM_007557	Bmp7	-0.012	2.001	5997	798	1254	5810	2243	4452
NM_011176	St14	0.028	1.999	4433	369	983	1442	3432	3890
NM_021390	Sall1	0.048	1.997	7736	150	1320	1063	622	3469
NM_001013756	Grhl3	0.027	1.997	1946	332	638	1818	4979	1941
NM_177099	Lefty2	0.065	1.995	1005	37	409	778	786	1380
NM_177775	D9Ertd280e	-0.065	1.991	2487	326	787	2948	4216	3944
NM_001033156	Fbxo33	0.283	1.989	5294	3871	3717	306	377	305
NM_025616	Timm50	0.988	1.988	3507	2399	2587	206	287	79
NM_030676	Nr5a2	-0.025	1.982	6991	874	2626	3993	8317	9840
NM_021876	Eed	0.485	1.979	6271	4627	3111	457	306	432
NM_001082532	Pigyl	-0.354	1.976	3820	1262	1893	80	66	18
NM_010436	H2afx	0.673	1.973	6237	3659	4116	40	25	11
NM_019674	Ppp4c	0.946	1.965	3591	3157	2436	226	198	88
NM_021308	Piwil2	-0.053	1.965	529	325	431	2280	1918	1987
NM_011155	Ppp5c	0.738	1.963	5233	3475	2914	512	485	273

NM_016875	Ybx2	0.190	1.962	1318	375	527	642	1908	1820
NM_153783	Paox	0.406	1.961	1972	1635	1290	177	151	116
NM_028314	2700097O09Rik	0.634	1.961	2060	1572	1419	720	976	539
NM_007921	Elf3	0.053	1.960	1199	46	107	211	574	327
NM_026507	Zwilch	0.964	1.959	5816	4992	4939	351	697	868
NM_053271	Rims2	-0.003	1.957	7731	4535	4572	11322	17920	23622
NM_133191	Eps8l2	0.013	1.956	1691	447	380	1622	1066	683
NM_001081150	Lonrf1	0.496	1.950	6036	4607	2767	1117	641	614
NM_030093	3300001G02Rik	0.794	1.947	2839	2049	2600	236	152	106
NM_178684	C130032J12Rik	0.438	1.946	2918	4324	3941	382	619	662
NM_008993	Pxmp2	0.051	1.938	3913	2697	2200	337	200	174
NM_010926	Cox4nb	-0.027	1.936	5877	4929	6012	474	443	414
NM_021287	Spnb3	0.070	1.936	4207	778	1314	3779	4055	4415
NM_016966	Phgdh	0.798	1.934	1996	4044	1894	687	750	189
NM_027891	1200011O22Rik	0.536	1.932	2778	3043	3451	386	474	290
NM_146176	Cnot3	0.461	1.929	5369	4211	3169	392	234	167
NM_026943	Snrpd2	0.688	1.928	2000	2343	1843	147	71	28
NM_001033966	Ak2	0.634	1.920	2130	3330	3063	571	350	170
NM_013769	Tjp3	0.014	1.919	3124	579	603	1078	3294	2313
NM_029441	Cdyl2	0.656	1.918	5855	3047	4347	3231	2669	3357
NM_001042670	ENSMUSG0000005	0.535	1.917	122	838	397	61	182	57
NM_133777	Ube2s	0.565	1.916	5247	5147	3474	59	60	63
NM_145706	Nup43	0.658	1.914	1957	1249	1701	219	387	392
NM_001012335	Mdk	0.006	1.914	1611	10	233	222	70	428
NM_030702	Senp3	-0.039	1.913	5783	3118	4090	296	207	226
NM_009904	Clgn	0.117	1.911	1224	218	572	1867	2852	4275
NM_023597	Wdr31	0.518	1.910	1367	1267	702	467	590	231
NM_009836	Cct3	0.947	1.909	6630	6276	3088	366	725	212
NM_026398	Pop5	0.114	1.906	4299	3573	2442	382	114	95

NM_011622	Tom1	-0.261	1.900	3520	2041	3165	1017	839	691
NM_001081176	Polr3g	0.398	1.895	5202	3677	5163	603	833	919
NM_029231	Pelp1	0.274	1.893	4566	2802	3555	371	307	398
NM_001037746	AU021838	0.558	1.890	2064	2919	1998	219	130	107
NM_025495	Cenpp	0.888	1.890	5233	6683	7166	3754	4428	5341
NM_177821	Ep300	0.607	1.890	6755	8493	7763	1140	1658	1760
NM_008894	Pold2	0.523	1.887	2086	1772	1912	253	246	225
NM_133834	Hnrnpf	0.779	1.886	13466	16522	11827	292	526	325
NM_029868	Gpbp1l1	0.413	1.881	4308	5666	2959	482	763	394
NM_008893	Pola2	0.565	1.873	3072	2537	3510	489	802	468
NM_026584	Gtf2e2	0.722	1.872	3299	3477	2494	608	644	536
NM_023635	Rab27a	-1.088	1.871	2397	583	1455	1375	2920	1973
NM_172723	Adap1	0.076	1.871	4070	587	971	1762	6273	3391
NM_019402	Pabpn1	0.779	1.867	3783	3300	3261	105	78	114
NM_009853	Cd68	0.106	1.865	182	146	142	99	56	65
NM_183275	1110002N22Rik	0.184	1.862	4793	2843	3111	204	139	203
NM_025928	Pmf1	0.870	1.860	3497	2082	1834	401	306	134
NM_133928	Chchd4	0.608	1.860	5956	7209	6381	229	306	254
NM_024173	Atp6v1g1	0.503	1.860	1791	4461	2644	135	222	67
NM_172135	Mterf	0.399	1.859	227	725	362	45	48	40
NM_001081203	Sbno1	0.981	1.854	5714	4867	3736	815	1246	754
NM_008316	Hus1	0.727	1.852	3245	2674	3337	432	461	458
NM_001083902	Slc12a8	-0.219	1.850	2769	1310	1748	5688	5927	5029
NM_025660	Ribc1	0.564	1.850	1944	1909	2101	66	218	124
NM_009876	Cdkn1c	-2.499	1.849	1582	271	811	2111	491	977
NM_001002929	Nup85	0.329	1.847	4217	3055	3164	437	468	349
NM_008567	Mcm6	0.966	1.847	3390	2289	2588	555	944	756
NM_019511	Ramp3	-0.018	1.847	1038	349	672	1729	1536	790
NM_011487	Stat4	-0.059	1.843	1429	706	828	2900	4710	7388

NM_013566	Itgb7	0.345	1.835	1034	387	415	854	746	875
NM_172562	Tada2l	0.348	1.834	3625	3402	3870	1257	1391	1354
NM_009077	Rpl18	0.396	1.833	7800	7165	4295	103	87	50
NM_001033759	Tmem2	0.127	1.827	4979	5739	6718	1724	2316	1710
NM_008838	Pigf	0.684	1.827	5891	5736	5653	599	884	801
NM_175930	Rapgef5	-0.239	1.827	5619	1593	2459	7212	8279	8130
NM_134013	Psme4	0.642	1.824	7251	5184	5625	2217	2058	2846
NM_013929	Siva1	0.600	1.824	4898	3844	3189	351	234	38
NM_026041	Rrp15	0.318	1.819	4967	4263	4257	413	631	598
NM_145429	Arrb2	0.715	1.818	2677	2157	2516	569	367	193
NM_008480	Lama1	-0.967	1.811	4934	2531	4970	3582	7517	4528
NM_007525	Bard1	0.643	1.810	3891	3569	3844	1219	1208	2161
NM_008558	Max	0.032	1.808	4266	4723	3674	576	796	522
NM_177475	Suhw2	0.096	1.806	3534	3111	3484	266	443	165
NM_011870	Cib1	0.150	1.806	2930	2598	2768	177	58	103
NR_001572	Nudc-ps1	0.663	1.802	24	30	29	34	106	26
NM_146252	Tbc1d13	-0.576	1.800	5030	3012	4385	386	348	334
NM_152915	Dner	0.349	1.798	4559	2232	3043	13737	11011	16505
NM_027250	2010305A19Rik	0.583	1.798	3317	1700	1214	230	204	147
NM_011552	Tcof1	0.644	1.798	3558	2230	2990	898	850	744
NM_026576	Etaa1	0.539	1.797	3182	3293	3392	305	242	363
NM_177613	Cdc34	-0.161	1.795	6282	4768	6291	180	232	195
NM_008184	Gstm6	-0.374	1.795	338	56	116	134	286	101
NM_145632	Polr2h	0.207	1.790	2613	2304	3002	102	40	118
NM_177897	B4galnt4	-0.073	1.787	4024	383	599	613	2355	1583
NM_027445	Rnf167	-0.267	1.787	4791	4264	4597	242	199	115
NM_174868	C030011O14Rik	0.504	1.785	3214	765	1166	869	1378	842
NM_153541	Zbtb8b	-0.051	1.783	1945	152	209	456	2512	1001
NM_145402	Tmem51	-2.419	1.771	4679	654	1901	1497	1768	1200

NM_139149	Fus	0.834	1.770	5506	4745	3582	216	217	267
NM_027123	2310010B21Rik	0.214	1.770	3161	3258	4110	210	203	281
NM_023626	Ing3	0.458	1.768	2596	3935	2385	421	908	740
NM_009423	Traf4	-0.145	1.762	6799	5865	7326	433	286	160
NM_010948	Nudc	0.948	1.759	4003	3831	2453	157	439	172
NM_175491	Smcr8	0.312	1.758	6769	4930	7631	151	80	75
NM_175263	Notum	-0.262	1.755	5680	375	1119	2055	1628	2204
NM_133815	Lbr	0.766	1.753	4880	4175	4678	809	499	591
NM_013608	Naca	0.138	1.750	8718	10657	7342	332	348	151
NM_010364	Gtf2h4	0.379	1.748	1990	2618	2552	143	264	222
NM_172567	Mettl2	0.416	1.747	4504	2684	3634	283	585	471
NM_009096	Rps6	0.461	1.744	6450	5751	3983	92	60	12
NM_007422	Adss	0.587	1.742	4029	5002	4954	723	801	846
NM_026065	D10Ertd322e	0.422	1.742	3764	3861	2937	392	501	527
NM_053122	Immp2l	0.416	1.741	5657	7522	5727	22600	19424	20419
NM_009211	Smarcc1	0.480	1.740	4375	3988	4029	1684	1923	2046
NM_173765	Aasdh	0.484	1.739	2913	3182	1825	723	588	296
NM_025304	Lcmt1	0.435	1.738	3685	2091	2102	1075	861	622
NM_010274	Gpd2	0.959	1.737	4314	5151	5624	2706	2431	3117
NM_001005223	Znhit3	0.357	1.732	3825	2472	2498	76	232	110
NM_001004365	Actr3b	0.012	1.731	5609	1436	1579	2151	4602	2177
NM_133985	Oxsr1	0.635	1.730	395	455	467	783	1258	1466
NM_019706	Rnf138	-0.055	1.729	6417	4622	5995	428	803	713
NM_030724	Uck2	0.611	1.724	8211	5189	7116	1741	1587	1446
NM_172735	Zc3hc1	0.986	1.723	1340	2368	1708	342	519	450
NM_007573	C1qbp	0.118	1.722	5406	3433	3502	255	225	124
NM_009532	Xrcc1	0.939	1.722	2255	1568	1696	622	700	304
NM_028603	2410081M15Rik	-0.820	1.721	4476	2157	2595	372	1579	209
NM_011274	C80913	0.764	1.721	4292	2955	2477	926	1204	752

NM_026871	Hint2	0.405	1.721	2543	2054	1813	149	152	69
NM_181410	Gtf2h3	0.618	1.718	3991	4267	2494	244	379	150
NM_027654	Pcgf6	0.357	1.715	2830	2753	3162	285	462	514
NM_172304	Tex10	0.892	1.708	3668	4641	2808	406	970	554
NM_007886	Dtnb	0.262	1.702	5385	3679	3365	5074	4497	3155
NM_017479	Myst4	-0.126	1.702	6460	5159	7230	2958	4160	4360
NM_008565	Mcm4	0.724	1.702	6807	3709	4762	228	378	434
NM_010021	Dazl	-0.079	1.701	350	236	441	681	582	1945
NM_010638	Klf9	-0.115	1.700	17318	9993	11331	456	781	534
NM_007475	Rplp0	0.680	1.699	2997	3328	2404	101	102	67
NM_178788	Dctd	0.595	1.696	2017	2093	2008	938	718	423
NM_153405	Rbm45	0.270	1.691	4585	3282	4656	283	145	391
NM_146036	Ahsa1	0.305	1.691	6514	5442	4236	170	258	110
NM_018776	Crlf3	0.454	1.690	4902	3526	3168	774	1104	838
NM_022653	Thop1	0.629	1.689	2191	2138	2258	409	765	369
NM_146116	Tubb2c	0.341	1.688	6579	3868	4694	125	10	25
NM_008239	Foxq1	0.081	1.688	6085	238	1978	1502	831	2185
NM_026453	Mak16	0.990	1.687	5354	3823	2776	159	90	133
NM_011390	Slc12a7	0.489	1.683	5760	3044	4025	1251	1479	1268
NM_021889	Syt9	0.413	1.682	5491	855	937	3965	4710	3203
NM_019505	Dgke	0.232	1.682	7735	3591	4702	476	607	707
NM_199042	Thap1	0.343	1.680	4768	3809	2871	236	142	100
NM_007858	Diap1	0.193	1.679	4578	3601	5156	1815	2271	2330
NM_178845	Zfp277	0.597	1.677	5088	4339	4983	2517	2442	1760
NM_021535	Smu1	0.739	1.675	3935	4021	2614	292	365	185
NM_177680	A730098D12Rik	0.775	1.674	3647	5097	2820	318	642	472
NM_199322	Dot1l	0.977	1.673	5456	6436	5247	1266	1380	849
NM_026964	Ccdc124	0.316	1.672	3490	2036	2262	116	82	75
NM_016706	Coil	-0.114	1.669	3320	1896	2239	493	362	363

NM_008630	Mt2	0.182	1.669	3387	1879	2318	119	32	62
NM_019685	Ruvbl1	0.616	1.668	2792	3965	2781	728	985	611
NM_145587	Sbk	-0.239	1.668	6354	1985	2669	1845	996	288
NM_207202	Ccdc120	0.078	1.667	570	102	473	182	208	126
NM_010256	Gart	-0.024	1.666	4577	3780	3765	555	527	634
NM_145974	C330016O10Rik	0.140	1.664	9441	2802	5157	652	374	273
NM_011288	Mrpl23	0.938	1.664	2337	2118	1136	351	234	62
NM_178891	Prmt6	0.522	1.664	4801	6817	4114	102	87	72
NM_011937	Gnpda1	0.544	1.663	4407	2982	3377	183	367	204
NM_011503	Stxbp2	0.905	1.660	2069	2777	2011	294	616	306
NM_172991	C030048B08Rik	0.721	1.659	6353	4515	3678	290	190	145
NM_172253	Twistnb	0.700	1.658	3787	2982	2487	229	298	171
NM_023059	Sigirr	-0.184	1.654	2834	139	760	309	1092	632
NM_183178	Fsd1	-0.587	1.652	1260	341	850	493	2819	313
NM_001081188	Exosc7	0.372	1.652	6231	4067	4938	488	460	367
NM_016907	Spint1	-0.013	1.651	6404	322	1002	592	2631	1910
NM_009266	Sephs2	0.070	1.651	4994	2716	2335	131	75	25
NM_028298	Zfp655	0.360	1.648	4847	6192	5244	229	375	355
NM_178618	2310040C09Rik	-0.161	1.647	3257	226	649	1793	1277	1570
NM_175045	Bcor	0.488	1.646	4956	3543	4700	3353	697	437
NM_011317	Khdrbs1	0.931	1.643	7266	6962	4817	374	626	356
NM_026030	Eif2s2	-0.791	1.641	8127	7037	5122	223	654	512
NM_175654	Hist1h4d	0.037	1.639	382	539	469	10	50	17
NM_178208	Hist1h4c	-0.248	1.638	145	194	248	21	37	44
NM_133351	Prss8	-0.047	1.637	1161	11	33	232	146	67
NM_178193	Hist1h4b	0.104	1.636	98	154	205	10	20	41
NM_145625	Eif4b	0.234	1.634	5983	4906	4983	521	414	569
NM_011914	Whsc2	0.886	1.634	7326	5195	3697	982	567	318
NM_012015	H2afy	0.487	1.633	5369	4541	6003	2059	1694	1292

NM_207212	Wtip	0.023	1.632	2961	2894	1841	450	448	170
NM_134084	Ppif	-0.264	1.632	2029	2633	3895	406	185	196
NM_016745	Atp2a3	0.224	1.631	2228	447	688	3404	3590	4961
NM_001039657	Mtl5	-0.157	1.623	1286	786	956	1140	1190	953
NM_019730	Nme3	0.050	1.622	4089	3301	3348	43	75	33
NM_146247	BC024814	0.547	1.613	2731	2040	2607	668	691	540
NM_001009951	BC088983	0.880	1.612	3395	3634	2591	419	280	251
NM_146218	Rfwd3	0.408	1.611	3061	2920	3390	465	282	399
NM_027000	Gtpbp4	0.399	1.607	5453	4880	5102	458	356	597
NM_008619	Mov10	-0.031	1.606	2989	4023	3101	405	562	160
NM_026551	Dcakd	0.066	1.604	2078	2186	1754	591	623	465
NM_007918	Eif4ebp1	0.810	1.603	8435	8012	4292	409	321	194
NM_025589	Rpl36al	0.384	1.602	5643	4753	3931	174	54	34
NM_175137	Vars2	0.594	1.597	2732	3610	3232	348	463	259
NM_019731	Nme4	-0.379	1.595	1998	1296	1930	282	209	175
NM_001024699	Zbtb45	0.534	1.594	4172	2104	1914	145	90	30
NM_029674	Got1l1	-2.526	1.594	58	17	10	189	489	76
NM_026549	Pdcd2l	0.576	1.594	4195	2660	2295	291	170	69
NM_028020	Cpsf3l	0.904	1.587	3946	4665	4120	324	294	249
NM_153173	Hist1h4h	-0.163	1.585	730	1329	1332	45	10	10
NM_025504	Atp5sl	0.016	1.583	1275	853	895	129	179	81
NM_145540	Ints3	0.528	1.582	3401	3590	2730	977	920	366
NM_030678	Gys1	0.321	1.578	5000	4505	3319	454	402	150
NM_026175	Sf3a1	0.910	1.578	3928	3527	3819	506	719	539
NM_019673	Actl6a	0.800	1.577	4640	4088	3089	265	462	267
NM_013892	Pcsk1n	0.095	1.576	1626	92	176	519	539	1103
NM_028006	Tube1	0.764	1.572	2537	3247	2891	277	269	494
NM_013754	Insl6	0.755	1.569	136	530	231	183	195	187
NM_022654	Lrdd	0.391	1.568	3125	1416	2197	274	171	58

NM_026028	Ccdc77	0.872	1.567	1550	2897	1319	345	737	579
NM_026080	Mrps24	-0.242	1.566	6064	2975	3661	160	111	49
NM_080470	Smc1b	-0.058	1.565	246	574	601	2791	2922	5527
NM_026543	3010026O09Rik	0.475	1.565	3900	2355	2213	790	741	658
NM_146074	Tfb1m	0.426	1.560	2070	2024	1765	956	999	1110
NM_009360	Tfam	0.168	1.560	3761	3202	4022	301	390	371
NM_172265	Eif2b5	0.241	1.560	3702	2676	3441	130	228	200
NM_011813	Fiz1	0.800	1.559	4466	1447	1419	250	125	95
NM_130881	Pabpc4	0.459	1.559	1564	2213	1952	247	366	191
NM_134021	Pnpo	-0.349	1.559	1839	1140	2961	314	842	155
NM_033077	D1Pas1	-0.071	1.557	663	32	58	510	133	108
NM_175031	Stk36	-0.353	1.555	2731	2517	3407	1470	1024	934
NM_133992	Usp52	0.238	1.552	2325	3183	2450	528	735	592
NM_025371	Acy1	0.493	1.549	894	802	931	302	135	189
NM_009902	Cldn3	-0.079	1.548	2791	20	287	176	1406	1766
NM_181406	Rars2	0.832	1.548	4829	5039	3251	1038	723	675
NM_026119	Med4	0.048	1.546	2970	2166	3339	167	170	311
NM_026932	Ebna1bp2	0.824	1.545	3298	3678	3167	177	205	108
NM_181444	Gprc5a	0.899	1.545	1577	1137	936	1124	1046	370
NM_019570	Rev1	-0.087	1.545	5221	3530	4553	1388	1181	2283
NM_145573	Mrps35	0.684	1.544	4516	4932	3580	648	1004	637
NM_017476	Akap8l	-0.502	1.543	8683	6844	6731	578	973	729
NM_025825	Appbp2	0.482	1.543	7309	6192	6580	899	1364	898
NM_025372	Tipin	0.855	1.543	3140	2382	2854	273	332	355
NM_029339	Ccdc101	0.752	1.540	2588	1471	1372	546	344	317
NM_134050	Rab15	-0.806	1.540	3150	676	1355	2323	3578	570
NM_027541	Prpf3	0.696	1.539	2132	2287	2012	419	449	268
NM_178700	Grsf1	0.875	1.538	4920	3769	3515	542	311	173
NM_029920	C130038G02Rik	-0.039	1.535	7963	2629	4112	14384	9169	12853

NM_028288	Cul4b	-0.292	1.535	3695	3642	3241	237	527	488
NM_138676	Shkbp1	0.422	1.534	1847	2628	1523	270	318	71
NM_172561	Spag7	-0.118	1.533	5175	4106	4406	224	152	101
NM_026591	Mrpl24	0.757	1.529	1381	2755	1082	66	89	42
NM_011830	Impdh2	0.355	1.529	2116	1618	1477	107	104	139
NM_178143	Prkaa2	0.207	1.527	3551	3554	1942	1924	2163	2712
NM_008847	Pip5k1a	0.968	1.526	4557	5423	2900	680	689	462
NM_144829	Aarsd1	-0.073	1.526	3478	2087	2602	360	252	305
NM_009272	Srm	0.434	1.525	4254	3957	4037	104	115	40
NM_010760	Magoh	0.853	1.523	2896	3529	1792	182	205	109
NM_008612	Mnat1	0.949	1.522	2370	3689	2450	3071	3140	2506
NM_011405	Slc7a7	-0.667	1.519	2126	680	2299	909	1565	1057
NM_178069	Lsg1	-0.075	1.518	2915	3427	3040	543	567	699
NM_027162	Mif4gd	-0.225	1.517	2916	1829	2128	266	161	80
NM_008449	Kif5c	0.549	1.516	3594	2038	2806	4904	5816	10420
NM_011275	Rnaseh1	0.386	1.516	3373	2976	2186	137	126	143
NM_027872	Slc46a3	-0.794	1.515	1413	1207	1802	455	596	285
NM_007999	Fen1	0.625	1.515	4601	3259	3720	101	183	95
NM_025553	Mrpl11	-0.090	1.514	2987	1830	2054	54	140	56
NM_133934	Isy1	0.877	1.512	2984	4216	3018	352	525	259
NM_134022	6330403K07Rik	-0.137	1.503	374	39	69	284	62	178
NM_027432	Wdr77	0.047	1.502	4932	4367	3392	145	374	122
NM_019537	Dscr2	-0.263	1.498	3053	2730	3144	169	434	373
NM_001080390	Mark2	0.427	1.497	608	189	302	690	533	501
NM_177619	Myst2	0.630	1.496	7490	5501	5315	874	805	696
NM_183392	Nup54	0.950	1.493	3056	3796	2339	354	488	249
NM_153544	BC030867	0.711	1.493	3478	1661	2056	766	669	432
NM_138681	Bcas3	0.370	1.491	6617	6689	6207	18026	16081	20257
NM_080848	Wdr5	0.125	1.488	22700	20106	26691	13718	12241	10606

NM_008432	Kcnu1	0.203	1.485	885	373	370	2695	3955	2158
NM_008956	Ptbp1	0.680	1.485	9828	9603	11321	418	311	294
NM_172993	Zfp512	0.681	1.485	557	1362	637	497	559	271
NM_011592	Timm44	0.701	1.483	2435	3182	1948	357	642	310
NM_020047	Tacstd2	-0.091	1.482	1610	41	121	1604	1448	1188
NM_011674	Ugt8a	0.475	1.481	2755	888	595	1989	2271	2519
NM_134010	Nup107	0.582	1.477	3963	4337	3357	811	908	945
NM_025876	Cdk5rap1	0.135	1.476	3690	2195	3095	478	651	684
NM_008628	Msh2	0.541	1.476	5341	3495	3597	826	1501	1130
NM_001005507	Smg7	0.409	1.474	6683	4001	5347	1108	1401	1908
NM_027171	2310057J16Rik	-0.131	1.471	5309	174	974	415	1676	893
NM_028041	Ddx54	0.722	1.470	5624	3597	2808	281	455	210
NM_011151	Ppm1b	0.998	1.469	5074	6078	4972	651	1265	1408
NM_010722	Lmnb2	0.682	1.468	4441	3301	3861	656	541	318
NM_001038010	Kat2a	0.220	1.463	2519	1626	2229	307	250	160
NM_029365	Med25	0.568	1.462	3772	2721	2565	332	330	187
NM_177672	AV028368	0.665	1.461	3465	2908	2745	605	255	112
NM_173396	Tgif2	0.380	1.456	6249	2860	3461	347	375	419
NM_022721	Fzd5	-1.516	1.455	12430	3666	5822	366	262	161
NM_027256	Ints4	0.922	1.452	2642	1940	1855	717	1139	737
NM_016905	Galk1	0.025	1.451	1675	2504	2642	239	352	151
NM_028597	Thoc3	0.159	1.451	4306	2683	3986	162	268	192
NM_030703	Cpn1	-0.028	1.451	746	286	414	1915	3013	2678
NM_001080387	Fusip1	0.791	1.450	4878	4932	3083	119	153	135
NM_031391	Gtf2a1	0.761	1.449	3485	4136	2406	586	904	462
NM_173755	Ube2o	-0.321	1.445	3893	3001	3870	1601	1104	887
NM_023565	Cse1l	0.743	1.444	5192	3495	4172	664	676	704
NM_054078	Baz2a	0.562	1.440	6452	12488	9237	738	1380	766
NM_144851	Senp1	0.578	1.439	4003	4121	4921	1234	1391	1488

NM_198671	Gse1	-0.160	1.439	13543	8593	12219	5430	2128	1954
NM_007892	E2f5	0.864	1.435	2871	3165	2027	561	278	208
NM_028053	Tmem38b	0.164	1.435	3144	2591	1825	484	783	427
NM_172284	Ddx19b	0.303	1.432	2997	2397	3120	245	311	386
NM_199056	Ippk	-0.036	1.432	5133	4267	6523	1044	1037	1228
NM_175414	9430079M16Rik	0.028	1.432	7409	9764	7161	6043	9203	4042
NM_030251	Abtb1	-0.066	1.430	4770	4639	4744	250	396	192
NM_145404	Prmt7	0.260	1.430	6749	4031	6674	725	739	1164
NM_133884	Gpn2	0.754	1.429	4743	4061	2958	205	209	77
NM_146000	Bud13	-0.049	1.424	2881	2647	3126	214	204	334
NM_013560	Hspb1	-2.297	1.424	2074	515	1840	54	54	45
NM_026824	Dus1l	0.102	1.423	4737	3432	4819	309	78	121
NM_023178	Dmap1	0.907	1.421	3511	2762	2227	149	108	129
NM_138756	Slc25a36	-0.407	1.420	4107	4254	3469	364	704	874
NM_019406	Fnbp1	0.002	1.419	6000	3772	6520	2162	2304	1845
NM_212450	Ctdspl2	0.348	1.419	5823	4690	4399	656	956	802
NM_026894	1500001M20Rik	0.848	1.418	3323	3588	2683	657	1235	691
NM_011178	Prtn3	-0.747	1.416	498	26	93	467	170	152
NM_008946	Psmb6	-0.233	1.414	1223	1828	1671	93	91	73
NM_153416	Aaas	0.337	1.413	3042	2680	2889	270	534	286
NM_027667	Arhgap19	0.362	1.412	3504	2939	2695	546	988	1913
NM_027423	Polr3b	0.817	1.409	3418	4564	3416	2831	2933	2774
NM_025923	Fancl	0.372	1.407	1820	1752	1465	1804	1697	1879
NM_001024954	Pbx4	0.396	1.407	2473	1091	1465	1024	808	1057
NM_016899	Rab25	-0.049	1.406	143	70	43	184	134	147
NM_016710	Nsbp1	0.758	1.406	978	1950	1331	92	177	97
NM_019805	Anapc7	0.872	1.405	4520	3119	2925	324	516	139
NM_026856	Zfp644	0.973	1.403	4795	4794	3189	1344	1896	1146
NM_001081293	Rprd2	0.633	1.403	6322	5582	3796	987	901	706

NM_001033314	C530028I08Rik	0.715	1.401	2743	2417	1727	715	214	242
NM_018877	Setdb1	0.600	1.397	1092	981	911	493	644	383
NM_029546	Pwp2	0.259	1.395	4489	2197	3984	392	337	281
NM_030096	Ddx52	0.585	1.394	2118	2799	2582	593	656	535
NM_007706	Socs2	-0.946	1.393	8352	7971	7784	416	485	161
NM_024221	Pdhb	0.047	1.393	3483	3075	3445	116	23	159
NM_175439	Mars2	0.694	1.391	4078	4754	5372	136	57	26
NM_198019	Cep78	0.935	1.391	3550	3089	3100	528	605	523
NM_020035	Pigo	0.412	1.389	1977	924	646	186	245	84
NM_021315	Noc3l	0.796	1.389	3600	3736	4116	686	858	685
NM_007949	Ercc2	0.214	1.387	2858	2237	2545	492	312	159
NM_027213	Med6	0.426	1.381	1173	2705	1148	237	375	292
NM_001013378	Uspl1	0.179	1.381	4552	5369	4803	614	589	798
NM_024177	Mrpl38	0.397	1.380	4026	2731	3061	388	179	141
NM_145975	Ddx46	0.880	1.379	4736	3076	3610	870	1683	1192
NM_009767	Chic1	0.745	1.374	2158	2747	2142	251	409	259
NM_145480	Rfc4	0.818	1.374	3788	2417	2890	272	349	416
NM_007438	Aldoa	0.986	1.373	4564	5621	4493	95	125	75
NM_028036	Tmco6	-0.416	1.372	1359	1526	1970	144	246	137
NM_001035123	Setd6	0.361	1.370	4764	2273	3447	115	78	104
NM_013415	Atp1b2	-0.358	1.369	3206	667	1487	1743	1204	1076
NM_020570	Xrcc2	0.881	1.369	2544	2383	1692	298	383	188
NM_016681	Chek2	0.614	1.369	4456	3471	2334	599	670	266
NM_008947	Psmc1	0.763	1.368	412	348	357	252	417	165
NM_001005509	Eif2a	0.887	1.365	3881	3782	2590	469	492	430
NM_133761	Dcp1a	0.267	1.360	3867	4307	4127	1045	1066	1459
NM_001033352	Klhl21	0.333	1.359	9238	8209	6607	461	253	58
NM_027485	Med26	-0.296	1.358	6405	5892	7965	1123	1063	1041
NM_133702	Nol11	0.253	1.358	4673	2895	3754	440	389	566

NM_197988	1190005I06Rik	0.109	1.358	2018	624	1783	1252	1009	654
NM_019795	Dnajc7	0.465	1.356	7799	6392	7440	921	1173	973
NM_023671	Clns1a	0.529	1.355	3498	2544	2586	185	352	162
NM_172598	Wdhd1	0.881	1.355	3951	4580	4526	603	1037	878
NM_177870	Slc5a6	0.697	1.353	2870	2594	2063	519	368	136
NM_023637	Sars2	0.147	1.352	3102	2242	1522	273	281	97
NM_008826	Pfkl	0.099	1.351	3579	3956	4397	1028	955	606
NM_010122	Eif2b4	0.387	1.351	6172	5701	4944	193	117	28
NM_197940	Wipf2	0.180	1.351	2741	2072	2846	1301	701	947
NM_007951	Erh	0.895	1.351	6531	5615	4201	196	288	142
NM_027545	Cwf19l2	0.661	1.350	1656	2599	1963	613	895	1215
NM_033571	Fkbp6	-0.051	1.350	618	502	424	2322	7640	5705
NM_023633	2410016O06Rik	0.730	1.349	5210	3412	3041	402	61	41
NM_023219	Slc5a4b	-0.028	1.348	525	593	407	2970	4002	3164
NM_172907	Olfml1	-0.174	1.347	166	117	92	594	973	396
NM_007707	Socs3	-1.803	1.346	11524	8544	14073	417	74	94
NM_027911	Raver1	0.537	1.345	3673	2836	3984	335	297	330
NM_008260	Foxa3	0.027	1.344	1885	1167	1062	891	1426	1059
NM_001081109	Lmtk2	-0.144	1.343	3940	2571	3476	1850	1813	1822
NM_025392	Bccip	0.619	1.341	4562	3692	2874	305	208	167
NM_178645	Blmh	-0.212	1.341	3273	3099	3680	1146	1475	1054
NM_021886	Cenph	0.625	1.340	3471	2267	3140	179	454	375
NM_133984	Hemk1	0.457	1.337	1792	1402	2105	281	146	412
NM_025818	1200014J11Rik	0.248	1.337	3489	3656	4816	671	545	889
NM_172644	5830468K18Rik	0.058	1.336	3330	4209	3770	542	546	781
NM_026277	Nob1	0.464	1.334	4225	2802	3133	186	411	247
NM_028814	2810403A07Rik	0.768	1.334	6092	5750	3983	467	609	242
NM_009447	Tuba4a	0.333	1.333	3379	3340	5415	459	260	240
NM_007989	Foxh1	0.020	1.333	2244	266	229	156	217	151

NM_145823	Pitpnc1	-0.404	1.331	10306	7011	10081	8331	9093	6292
NM_177298	9030221M09Rik	0.082	1.330	4389	6539	4793	785	815	499
NM_010590	Jub	-0.067	1.329	11848	9140	11223	371	337	183
NM_025573	Sfrs9	0.878	1.329	4517	4936	3573	235	155	25
NM_145620	Rrp9	0.431	1.329	2596	3106	4278	208	163	184
NM_153585	Cnot10	0.460	1.328	3754	2585	3507	1027	770	1127
NM_198609	BC003885	0.228	1.328	3892	2753	3492	104	154	130
NM_021882	Si	-0.345	1.327	665	792	634	362	834	473
NM_007690	Chd1	0.608	1.326	4793	5286	5117	1100	2075	1601
NM_172713	Sdad1	0.990	1.325	3807	4163	2970	456	649	290
NM_009095	Rps5	0.013	1.325	4703	4846	3069	208	144	85
NM_001081107	Hel308	0.623	1.322	3990	3841	2752	790	732	483
NM_009319	Tarbp2	0.445	1.322	4064	4420	4267	173	260	155
NM_145890	Grhl1	-0.126	1.320	426	458	397	1038	1263	505
NM_009085	Polr1c	0.246	1.318	6428	6680	6308	124	74	117
NM_144918	Smyd5	0.350	1.317	3674	4399	3281	288	496	429
NM_022811	Polr1e	0.502	1.316	2878	2482	2464	384	518	159
NM_010709	Lgtn	0.161	1.316	2567	1955	2127	400	446	450
NM_010882	Ndn	-2.191	1.314	1889	23	458	431	47	60
NM_172705	Phf13	0.093	1.313	5900	6258	4305	544	240	97
NM_178763	Zfp750	0.078	1.313	757	54	105	405	419	272
NM_001037741	Gpx4	0.041	1.311	6361	4918	3993	171	76	60
NM_201230	Fgfr1op	0.659	1.311	237	451	437	400	814	714
NM_177305	Arl4c	0.675	1.311	7359	9691	10499	767	138	105
NM_201637	Chd8	0.034	1.307	619	857	828	706	873	1045
NM_019870	Ard1a	0.790	1.306	2901	1621	1677	85	139	67
NM_029360	Tm4sf5	0.135	1.305	169	33	83	418	662	371
NM_025546	Rsl1d1	0.526	1.305	5414	5903	5204	324	322	365
NM_027184	Ipmk	-0.265	1.305	5654	4067	5524	844	1045	790

NM_001024922	Ddx49	-0.419	1.305	4417	4349	4943	285	132	233
NM_025686	Brf2	0.260	1.304	2753	2380	1599	112	98	41
NM_138630	Arhgap4	0.193	1.304	327	111	66	312	322	214
NM_001033548	6030429G01Rik	-0.499	1.303	827	1353	1776	467	266	165
NM_176848	Fbxo2	-1.481	1.302	2399	1464	1401	1661	2179	1335
NM_177777	D11Wsu47e	-0.278	1.302	6144	4146	5033	208	112	249
NM_009801	Car2	0.010	1.302	3216	323	1383	1087	466	252
NM_145447	Mfsd7c	-0.378	1.300	2021	1128	1061	2744	4747	1368
NM_019716	Orc6l	0.234	1.300	8861	6752	8701	82	52	373
NM_145993	L3mbtl2	0.470	1.300	4224	3227	3400	544	901	552
NM_145147	Gtpbp6	0.218	1.298	7831	3909	3569	174	170	65
NM_030722	Pum1	0.585	1.298	7653	6865	3934	1847	2526	1498
NM_024236	Qdpr	0.165	1.297	1965	2489	2111	522	231	253
NM_001013391	Cpsf6	0.671	1.297	5805	8711	6159	422	766	844
NM_138660	Casc3	0.545	1.297	3417	3636	3607	436	318	615
NM_175660	Hist1h2ab	0.123	1.296	176	303	270	24	10	29
NM_178029	Setd1a	0.678	1.296	5200	4296	3374	387	454	221
NM_022994	Dap3	0.373	1.295	6723	4493	4066	521	715	319
NM_133242	Rnpc2	0.012	1.294	5716	8452	8291	480	314	683
NM_007533	Bckdha	-0.381	1.294	3620	2977	1987	874	1033	425
NM_008692	Nfyc	0.595	1.293	4042	5050	3546	1204	1568	944
NM_001076789	Cbx5	0.793	1.293	5564	5835	4400	593	1295	1383
NM_001079824	Hnrnph3	-0.004	1.293	5784	6017	5803	216	203	302
NM_008562	Mcl1	0.157	1.292	10471	9825	5283	136	63	66
NM_001033446	Gm949	0.071	1.291	1287	232	118	271	538	366
NM_020329	Dolpp1	-0.358	1.289	2921	2555	4201	278	134	198
NM_028908	4933403G14Rik	-0.663	1.289	1987	62	450	454	211	151
NM_130879	Usp48	0.259	1.288	9295	5824	3921	1201	1426	639
NM_011624	Top3b	-0.337	1.287	3928	3419	3554	542	695	644

NM_007661	Cdc2l1	0.780	1.285	5353	4629	3105	509	429	225
NM_011542	Tcea3	-1.712	1.284	2903	609	2993	1644	3149	609
NM_027294	Cmtm8	-0.554	1.283	2638	490	1739	3173	3776	2950
NM_031248	Robld3	0.231	1.283	4484	3309	2803	179	130	41
NM_144824	BC021790	0.278	1.282	5358	4204	4056	553	637	497
NM_023731	Ccdc86	0.224	1.282	3723	3647	4912	285	238	184
NM_010655	Kpna2	0.461	1.281	7481	5381	4418	235	365	227
NM_027204	Mrpl12	0.099	1.281	6381	3199	4760	126	121	56
NM_023514	Mrps9	0.572	1.280	3770	2668	3496	1288	1013	1498
NM_134012	Mbtd1	0.153	1.279	5315	4525	6068	1062	1520	1661
NM_026508	Trap1	0.013	1.278	4849	3857	4375	650	595	798
NM_011400	Slc2a1	0.255	1.275	5719	4304	3115	744	586	280
NM_020046	Dhodh	-0.103	1.274	2728	1972	2332	295	194	335
NM_178215	Hist2h3b	0.088	1.273	416	479	230	16	30	10
NM_009743	Bcl2l1	0.356	1.272	4493	14458	8035	928	1016	960
NM_175138	Dnaic1	-0.555	1.270	2395	2265	2089	4472	6031	2317
NM_144958	Eif4a1	0.435	1.270	9867	5796	5872	214	158	98
NM_030697	Kank3	-0.841	1.268	5953	2311	3396	540	1093	532
NM_080446	Helb	-0.136	1.266	2997	3502	2819	711	907	815
NM_172745	Tufm	0.631	1.266	2517	1992	1885	100	38	73
NM_001017525	Btbd11	-0.130	1.264	1399	2121	1983	2780	4146	3193
NM_009109	Ryr1	0.007	1.264	6430	1090	1587	6139	9417	5405
NM_011589	Timeless	0.655	1.264	366	542	880	346	590	414
NM_009606	Acta1	-0.111	1.262	260	41	190	674	1129	1796
NM_025598	2700038C09Rik	0.076	1.261	3483	2090	3114	70	42	26
NM_172813	Enox1	0.949	1.260	9254	5696	8311	19962	15059	21057
NM_018749	Eif3d	0.464	1.254	6337	6860	6947	250	373	376
NM_009253	Serpina3m	0.202	1.254	28	36	10	214	339	78
NM_152822	Las1l	0.620	1.254	2061	2099	2362	213	434	226

NM_027288	Manba	-0.725	1.254	6713	3378	3016	1400	1795	805
NM_020486	Bcam	0.095	1.252	2005	202	513	388	1253	187
NM_173453	Tmem11	0.436	1.251	7130	5902	6185	449	409	301
NM_144543	Thyn1	0.576	1.251	3402	3816	4397	150	60	69
NM_027678	Zranb3	0.451	1.250	3966	3220	3240	3395	3852	3959
NM_007383	Acads	0.075	1.248	2659	2173	1833	183	222	145
NM_145123	Crtac1	-0.135	1.243	4949	1674	1763	9618	13544	12105
NM_020580	Th1l	0.501	1.243	2660	2018	2532	322	296	229
NM_001045531	Spata22	-0.107	1.241	138	120	199	1151	462	1374
NM_198005	4833418A01Rik	-0.157	1.239	3678	3063	3617	487	345	579
NM_145610	Ppan	0.048	1.238	5820	3729	4239	171	75	94
NM_134109	Ildr1	0.040	1.237	4289	272	307	1332	1253	1387
NM_007926	Scye1	0.537	1.233	4590	5314	3235	249	600	196
NM_016959	Rps3a	-0.307	1.227	6892	5228	3628	91	185	74
NM_008787	Pcnt	0.509	1.226	5140	3195	3597	2052	2370	1886
NM_011750	Sf1	0.322	1.225	6643	5279	5591	469	548	385
NM_009634	Adsl	0.658	1.223	3808	3704	3617	377	416	367
NM_178379	Cox10	0.782	1.223	5354	4901	5127	3440	3185	3378
NM_008033	Fnta	0.713	1.223	2283	2339	1894	283	254	169
NM_134087	AA409316	0.022	1.220	5787	3657	4959	1490	1104	740
NM_001034059	6330534C20Rik	0.039	1.220	292	93	97	114	196	356
NM_199466	Eml4	0.283	1.219	4401	5286	4358	2231	3447	3526
NM_009359	Tex9	0.782	1.217	2655	2202	2260	482	507	770
NM_018810	Mkrn1	0.248	1.216	9031	6075	5343	347	735	371
NM_016723	Uchl3	0.309	1.216	3093	2080	3516	795	876	1561
NM_011568	Thoc4	0.657	1.216	5374	4107	4284	189	245	58
NM_031877	Wasf1	0.675	1.212	5354	5142	6056	1199	1277	1899
NM_007382	Acadm	0.196	1.212	2761	3005	2302	186	270	243
NM_026411	1700021F05Rik	0.154	1.212	2991	3132	3284	470	512	430

NM_030178	Brpf1	0.871	1.211	5098	5939	5492	324	637	379
NM_011972	Poli	0.018	1.211	1671	2658	2436	258	394	531
NM_177342	Taf5	0.539	1.208	2821	3105	3160	190	369	474
NM_175112	Rae1	0.302	1.208	3636	3258	4135	281	475	265
NM_177266	A930009M04Rik	-0.082	1.207	4277	2872	3502	644	666	950
NM_026034	Armc10	0.882	1.207	2084	2878	1767	232	323	132
NM_177474	D19Bwg1357e	0.397	1.207	4417	5435	4896	713	704	1080
NM_133664	Lad1	-0.021	1.203	1882	201	452	2454	3621	3437
NM_016737	Stip1	0.016	1.201	4095	3826	4088	343	401	316
NM_010124	Eif4ebp2	-0.148	1.201	5582	4344	4889	462	641	435
NM_011900	Mpdu1	-0.179	1.200	3133	1259	1644	254	146	121
NM_146173	Tspan33	0.072	1.199	2124	582	848	1714	6037	3072
NM_144820	Ccdc28a	0.191	1.198	3550	2009	2295	476	437	476
NM_145995	2700050L05Rik	0.436	1.196	98	194	125	327	384	208
NM_172122	Crocc	0.710	1.193	2629	446	228	1050	1065	451
NM_026071	Slc25a19	0.225	1.193	3673	2268	2786	393	468	273
NM_022988	Nif3l1	0.153	1.192	225	650	550	363	328	405
NM_025663	Gpatch4	0.649	1.191	2938	2166	1765	270	244	104
NM_016884	Hnrnpc	0.890	1.191	4394	5242	3532	535	490	681
NM_080469	Prnpip1	0.314	1.189	2902	4128	2939	2792	2702	1452
NM_175238	Rif1	0.504	1.188	5302	2779	3281	708	698	1360
NM_080289	Grhpr	0.331	1.188	2762	2224	2030	277	240	104
NM_026167	Klhl13	-0.128	1.185	2514	1444	2052	952	1806	2102
NM_198029	Fermt1	-0.115	1.184	1053	197	420	2600	2615	2922
NM_021556	Mrps30	0.327	1.184	4278	4433	4870	120	205	205
NM_198631	BC057627	0.631	1.183	5652	8209	5060	810	499	405
NM_026816	Gtf2f2	-0.235	1.183	3045	4132	4509	2513	1862	2973
NM_145506	Epb4.1l5	0.314	1.183	4288	3478	3573	2680	2445	4239
NM_016680	Sfrs16	0.214	1.183	3993	2950	2794	786	939	357

NM_010472	Agfg1	0.619	1.181	4786	4243	4699	1027	1313	1605
NM_019990	Stard10	-0.130	1.181	2723	1160	1183	2246	1166	506
NM_025572	2610528J11Rik	0.125	1.181	166	43	34	154	181	87
NM_172469	Clic6	-0.146	1.181	1525	417	800	3036	4953	4447
NM_153505	Nckap1l	0.578	1.179	296	576	358	2529	1861	2994
NM_011916	Xrn1	0.350	1.179	4357	4371	5070	1507	1724	2238
NM_008799	Pdcd2	0.419	1.179	3482	4563	4083	156	238	245
NM_019976	Psrc1	0.083	1.179	4708	3085	2548	152	312	30
NM_172662	Gtdc1	0.728	1.178	936	1469	1803	5820	4181	8348
NM_011945	Map3k1	0.072	1.178	6134	5150	6274	2369	1923	1420
NM_133835	Ubac1	-0.098	1.178	2846	2067	3483	480	466	545
NM_026393	Nmral1	0.569	1.177	1513	1591	1276	193	104	197
NM_007705	Cirbp	0.730	1.177	2774	3054	2374	124	175	100
NM_023202	Ndufa7	0.265	1.176	4023	4471	2782	160	457	334
NM_024240	Gins4	0.567	1.176	2955	2088	2039	179	210	117
NM_009478	Urod	0.526	1.176	2470	2699	2175	199	194	54
NM_023479	Elac2	0.389	1.175	3492	3247	3688	528	587	578
NM_008586	Mep1b	-0.055	1.175	258	285	217	584	1204	1633
NM_153775	2310061F22Rik	0.284	1.175	4281	3835	5798	208	205	98
NM_028273	Pgam5	0.995	1.174	4725	4043	3026	194	242	93
NM_183020	Atxn2l	0.345	1.174	6901	5853	4636	317	269	209
NM_029557	Tsen54	-0.271	1.173	4315	2698	4861	380	292	75
NM_023130	Raly	0.010	1.173	4914	5662	6325	1143	1313	1614
NM_177700	Atmin	-0.157	1.171	4744	2819	3980	303	236	336
NM_172410	Nup93	-0.025	1.171	3058	3212	4968	1594	1758	2251
NM_001077264	Ap2a1	0.326	1.171	4242	3128	2276	718	728	305
NM_198102	Tra2a	0.786	1.171	6650	8336	5552	237	351	331
NM_020579	B4galt3	-0.064	1.169	3019	2747	3523	271	103	274
NM_054048	Rcor2	0.316	1.168	3711	2141	1726	143	187	133

NM_198613	Ap2s1	0.147	1.167	1529	2386	1830	318	122	95
NM_010588	Jag2	-0.205	1.167	5358	493	830	2314	3182	1803
NM_009709	Arnt	0.690	1.166	3592	3872	2463	722	1077	734
NM_016776	Mybbp1a	0.720	1.163	5045	3158	4023	383	257	259
NM_025380	Eef1e1	0.654	1.162	4078	3175	3255	269	302	403
NM_133185	Rogdi	-0.260	1.161	2161	1255	2027	212	140	163
NM_001013765	Zscan4c	0.210	1.160	20	10	10	18	10	10
NM_009384	Tiam1	-0.824	1.160	3238	2173	3650	7674	4624	5881
NM_011081	Piga	0.576	1.160	1639	1280	1617	198	365	199
NM_025463	1810009A15Rik	-0.006	1.157	3049	2916	2795	58	68	45
NM_025407	Uqcrc1	0.127	1.157	5422	3488	4293	264	242	264
NM_001039385	Vgf	-0.202	1.157	2337	912	1196	1168	1507	636
NM_024199	Cstf1	0.476	1.153	5479	5012	5282	221	257	271
NM_020491	Sssca1	0.237	1.153	2650	2110	2517	37	116	36
NM_001004366	Scube3	0.057	1.150	3434	976	945	2854	8153	5556
NM_001014974	Ttll4	0.433	1.149	3035	2914	3522	707	823	944
NM_001042675	Rbpms	-1.561	1.149	7659	4416	4887	2425	4562	1196
NM_178576	Cpsf4	0.574	1.149	4917	5024	5254	349	326	239
NM_007880	Arid3a	-0.131	1.148	5087	7213	7578	1455	1015	660
NM_134139	Wdr74	0.220	1.147	3853	2404	3096	164	75	127
NM_027644	4931440B09Rik	0.073	1.145	889	76	38	323	851	263
NM_025666	Ubr7	0.428	1.145	4978	5379	3819	419	606	167
NM_012003	Cops7a	0.412	1.144	3956	5439	4287	215	276	161
NM_172116	Pddc1	-0.012	1.142	2410	2673	2640	149	167	71
NM_013456	Actn3	-0.166	1.142	2005	1342	1719	1011	1976	870
NM_133787	Nmd3	0.553	1.141	5247	4864	3530	272	649	244
NM_019995	D3Ertd300e	0.811	1.139	2311	3238	1648	275	678	295
NM_013782	Ptdss2	0.230	1.139	3597	2371	2243	517	578	349
NM_025909	Oma1	0.476	1.139	1634	2093	1369	999	1008	493

NM_025384	Dnajc15	0.032	1.139	2764	1818	3042	1064	966	1667
NM_178747	Gulo	0.103	1.138	404	200	223	1137	2011	1300
NM_028469	3110082I17Rik	0.317	1.136	6589	5998	8761	3298	2897	2299
NM_023197	2310008H09Rik	0.544	1.136	5782	3816	3395	277	205	136
NM_025537	Tsfm	0.180	1.135	2961	3117	2382	273	286	180
NM_025996	Tomm34	-0.121	1.133	4566	3560	5070	404	305	308
NM_144871	Suv420h1	0.878	1.132	4764	4686	5249	1235	1120	1271
NM_144826	Utp6	-0.849	1.131	4074	2705	2735	799	806	576
NM_029278	Nol14	0.714	1.130	4128	4712	3025	523	451	239
NM_026758	Mphosph6	0.360	1.130	3549	3123	3534	239	294	171
NM_001029990	Mett11d1	0.035	1.129	2745	2273	3246	181	176	179
NM_026370	Myst1	0.297	1.129	1824	1830	1538	281	328	122
NM_016766	Mcrs1	0.144	1.128	3154	2348	2416	292	210	287
NM_029456	Saps3	0.188	1.128	5361	5423	5446	2004	2464	2900
NM_025348	Ndufa3	0.427	1.127	2456	2098	2193	54	104	10
NM_029963	Mrps5	0.067	1.127	5570	3309	3689	478	325	297
NM_007444	Amd2	0.337	1.126	5515	5055	5469	240	453	193
NM_010774	Mbd4	0.340	1.125	3041	3159	3005	174	298	428
NM_008952	Pipox	0.063	1.124	559	141	207	660	1093	1713
NM_016748	Ctps	0.825	1.123	4993	5323	3940	563	535	322
NM_021500	Maea	-0.048	1.120	4327	5366	3760	747	1110	540
NM_028003	Rpap3	0.330	1.120	3428	3234	4192	469	1162	707
NM_007531	Phb2	0.860	1.118	6564	8206	5898	136	161	104
NM_173002	Zxdc	0.904	1.118	4545	9038	7809	1116	1026	774
NM_009031	Rbbp7	0.065	1.117	3022	2804	2795	214	100	163
NM_026037	Mboat2	0.784	1.117	5009	2959	2053	3779	4749	4682
NM_001005525	Rsrc2	0.888	1.116	3344	5935	2903	359	473	253
NM_026035	Mrpl55	0.087	1.116	4452	2184	3990	165	214	114
NM_178610	Krr1	0.473	1.116	2867	3990	3259	389	356	387

NM_019869	Rbm14	0.095	1.115	10076	6116	6954	303	360	190
NM_145462	D14Ertd500e	0.178	1.114	4128	2842	3088	390	240	269
NM_026176	Pdcl	-0.323	1.114	2434	2609	2705	135	141	247
NM_172992	Phtf2	0.586	1.113	5556	5176	3202	1494	2104	1706
NM_001017426	Jmjd3	0.288	1.112	10256	9484	9414	1450	577	352
NM_019426	Atf7ip	0.792	1.111	2668	7793	3250	1308	2320	1931
NM_134059	Ddx41	-0.329	1.111	4207	2836	4315	245	112	88
NM_008951	Psmd4	0.687	1.109	2396	2722	1786	157	270	109
NM_008887	Phox2a	-0.074	1.106	2625	62	418	1569	902	1064
NM_008281	Hpn	-0.092	1.105	916	147	148	989	782	292
NM_021451	Pmaip1	0.286	1.104	4364	2061	3355	1232	372	256
NM_011752	Zfp259	0.250	1.103	4818	3609	5063	275	196	238
NM_008992	Abcd4	0.671	1.102	2452	1970	1591	559	474	187
NM_001029876	Urb2	-0.323	1.101	279	374	597	390	422	824
NM_009372	Tgif1	0.337	1.101	8747	18651	10035	182	232	253
NM_026533	Rps13	0.434	1.099	4685	5177	2914	99	93	10
NM_181590	Shq1	0.475	1.098	4480	5590	4198	1969	3384	2584
NM_025845	Prpf38b	0.530	1.096	6207	6759	4984	157	258	74
NM_134141	Ciapin1	0.717	1.095	5197	4530	5923	328	395	256
NM_172718	Sgsm1	-0.956	1.095	4207	857	1799	3743	2271	1545
NM_009837	Cct4	0.560	1.092	5674	5440	4951	367	96	397
NM_025567	Cyc1	0.356	1.092	5991	4102	5671	88	115	89
NM_133971	Ankrd10	-0.063	1.091	8453	6356	6845	544	617	440
NM_178601	Imp4	0.679	1.091	6004	3360	4380	163	336	191
NM_025302	Mrpl2	0.486	1.090	4290	3926	4256	111	150	134
NM_008439	Khk	-0.026	1.090	2024	1226	518	663	540	133
NM_175485	Prtg	0.246	1.090	4006	3714	4157	4494	1759	3432
NM_018872	Tmem131	-0.018	1.084	8228	5766	6015	3149	3540	4137
NM_026665	Cep57	0.493	1.084	6806	6139	6777	338	407	387

NM_026171	Nvl	0.065	1.084	3578	2623	3136	875	1023	1182
NM_177379	Grit	0.446	1.082	329	252	320	921	1058	1341
NM_025910	Mina	-0.015	1.082	2105	1729	2723	288	512	532
NM_139198	Plac8	0.539	1.082	227	1172	232	723	600	310
NM_026068	Med31	0.428	1.080	3867	3444	3313	283	177	59
NM_138653	Bspry	0.122	1.080	1066	168	141	772	1545	362
NM_011967	Psma5	0.849	1.079	2831	3991	3162	367	409	232
NM_144925	Tnrc6a	0.794	1.078	3280	3681	2715	1256	969	1037
NM_029891	Nkrf	0.339	1.078	1674	1928	2087	155	305	295
NM_173757	Mrps27	0.282	1.075	4811	4183	4515	1304	1659	1576
NM_009940	Coq7	0.240	1.072	2723	1848	1171	178	45	79
NM_013614	Odc1	0.273	1.071	4145	4158	3832	288	183	113
NM_001081267	Rsf1	0.578	1.071	6507	5518	4641	1482	1650	1234
NM_023324	Peli1	0.193	1.071	3847	4300	4230	1425	1582	1729
NM_145569	Mat2a	0.409	1.071	6630	12590	7122	102	223	201
NM_007764	Crkl	0.420	1.070	6637	7140	7621	715	798	1124
NM_008052	Dtx1	-0.182	1.070	5036	430	1677	3990	2041	1817
NM_016755	Atp5j	-0.445	1.068	6144	5389	6293	278	217	168
NM_028487	Gpbp1	-0.034	1.068	6212	4578	5436	730	1520	1811
NM_028812	Gtf2e1	-0.145	1.068	3547	3163	3428	510	652	787
NM_172755	Sfrs14	0.478	1.068	4084	3244	4046	492	690	813
NM_011700	Vill	-0.025	1.066	827	280	765	1306	1356	499
NM_009708	Rnd2	-0.348	1.066	2699	1618	2260	522	166	105
NM_011294	Rpo2tc1	-0.370	1.066	3663	4581	4354	228	468	462
NM_029036	S100pbp	0.483	1.066	5913	6392	4238	571	735	459
NM_008133	Lgsn	-0.215	1.064	7281	6258	6602	538	780	1072
NM_001040686	Zfp692	0.284	1.063	2424	1884	2192	374	301	138
NM_010473	Hrc	0.386	1.063	486	486	375	266	1132	400
NM_029976	Cdkn2aipnl	0.917	1.062	3007	2714	2465	260	263	186

NM_011729	Ercc5	-0.641	1.062	2635	3388	3788	709	872	914
NM_026728	Echdc2	-1.016	1.061	1854	1432	1707	415	1474	354
NM_177301	Hnrpl	0.888	1.061	4262	4537	2755	300	161	70
NM_175394	Wtap	0.595	1.061	5678	5140	4867	403	412	432
NM_018790	Arc	-0.048	1.061	4267	1341	906	585	400	893
NM_026476	2610101N10Rik	0.731	1.060	6406	5253	5280	918	902	1417
NM_025886	Rassf7	0.605	1.058	3530	1368	1394	131	147	54
NM_183417	Cdk2	0.583	1.057	2638	4680	3608	290	355	290
NM_033079	D6Mm5e	-0.009	1.056	488	1534	882	4509	3952	3946
NM_025301	Mrpl17	0.437	1.055	4809	4189	2720	264	224	136
NM_152810	Cdc5l	0.448	1.055	7234	6811	6412	622	1379	1044
NM_010435	Hira	0.491	1.052	8809	5647	7876	1952	1991	2391
NM_172404	Ccbl1	0.000	1.051	2918	2769	3947	554	447	660
NM_028775	Cyp2s1	-0.123	1.047	1480	151	433	560	870	463
NM_011971	Psmb3	-0.096	1.047	5440	3782	4259	371	359	275
NM_009081	Rpl28	0.514	1.045	5098	3828	2359	12	45	13
NM_008193	Guk1	-0.575	1.045	4186	2177	3211	360	329	186
NM_001081746	EG665378	0.048	1.044	14	122	396	103	97	96
NM_028760	Cep55	0.879	1.043	4284	4109	3811	409	414	559
NM_019761	Nxt1	0.286	1.043	2509	3528	2874	40	42	63
NM_198108	Morn4	0.871	1.043	2767	1514	1384	301	429	381
NM_027292	Bzrpl1	0.013	1.043	200	93	25	292	274	174
NM_001033463	Tatdn2	0.488	1.043	4240	5910	5199	263	381	296
NM_010613	Khsrp	0.539	1.039	4054	5458	4069	420	301	244
NM_008802	Pde7a	0.492	1.039	377	255	209	613	818	483
NM_145583	Frag1	-0.165	1.038	1421	1529	1301	196	389	89
NM_009131	Clec11a	0.600	1.038	2392	1189	1092	428	276	114
NM_020569	Park7	0.482	1.036	1409	1713	1354	378	359	122
NM_130892	Rtn4ip1	0.004	1.036	2826	3324	2775	1430	769	1475

NM_054087	Slc19a2	-0.317	1.036	7138	5096	6235	546	331	485
NM_028875	Xrcc3	0.641	1.035	3473	3101	2721	450	347	137
NM_007554	Bmp4	-3.102	1.031	4346	478	1684	1252	1718	3693
NM_013462	Adrb3	-0.129	1.031	8084	406	1917	290	568	391
NM_019937	Ccnl1	0.308	1.031	5899	9144	3953	215	382	248
NM_020024	Taf10	0.615	1.030	1919	1595	1576	38	54	22
NM_009795	Capns1	0.398	1.029	4299	4143	2533	135	103	79
NM_146089	Ccdc5	0.152	1.026	2335	2109	2195	153	177	205
NM_011192	Psme3	0.265	1.025	5079	3745	4795	271	141	164
NM_016867	Gipc2	0.356	1.024	4562	577	1092	3314	1915	1392
NM_018747	Akap7	0.633	1.024	3153	3304	3103	4187	3273	4525
NM_026989	Sfrs11	0.586	1.024	21	43	61	208	283	135
NM_183311	B930076A02	0.025	1.023	2331	487	1026	1375	2880	1699
NM_001013372	Nrp	-0.080	1.023	3702	4208	2626	13	12	34
NM_009391	Ran	0.371	1.023	6974	5644	5165	95	81	36
NM_021559	Zfp191	0.164	1.021	3477	4012	3829	228	168	246
NM_138669	Eif4a3	-0.148	1.020	5511	3543	4263	211	392	350
NM_026603	Denr	0.556	1.020	3933	3050	2698	403	508	276
NM_016714	Nup50	0.165	1.019	5052	5852	6013	313	475	467
NM_199195	Bckdhb	-0.239	1.019	844	1414	3196	3237	3861	4723
NM_025582	2810405K02Rik	-0.552	1.018	1430	746	2121	267	411	89
NM_030680	Upf1	0.289	1.017	5223	4804	5675	969	484	478
NM_010852	Myef2	0.812	1.016	188	123	147	447	415	567
NM_001044744	Gcdh	-0.754	1.015	2783	2028	3825	223	138	345
NM_026120	2410127L17Rik	-0.146	1.015	4223	3007	2957	545	760	780
NM_008471	Krt19	0.782	1.014	1529	623	302	824	410	696
NM_023524	Tfpt	0.423	1.012	4064	3130	2836	197	153	163
NM_007765	Crmp1	0.657	1.011	2368	1626	1800	1951	2300	1113
NM_001007465	Rffl	-0.176	1.010	4842	2963	3988	2811	1587	1303

NM_010178	Fusip1	0.113	1.009	4878	5003	3107	168	222	153
NM_153062	Slc37a1	-0.007	1.008	3196	1741	1985	1808	4806	3533
NM_026654	Toe1	0.717	1.004	4560	3195	2674	162	131	29
NM_013742	Cars	0.303	1.002	6900	5979	4150	927	943	386
NR_003642	2900062L11Rik	-1.539	1.001	186	89	228	10	55	25
NM_011943	Map2k6	0.395	1.001	1615	3702	2170	6420	4425	3487
NM_016876	Eif3s4	0.303	1.001	3146	3232	4078	136	206	98
NM_008102	Gch1	-0.319	1.001	3045	1104	3028	1905	1989	1320
NM_025337	Akr7a5	-0.041	1.000	4980	3764	3963	324	223	55
