# Supplementary material for: Identification of the early and late responder genes during the generation of induced pluripotent stem cells from mouse fibroblasts
Source: PLoS One. 2017 Feb 2;12(2):e0171300. doi: 10.1371/journal.pone.0171300 (PMC5289558; doi:10.1371/journal.pone.0171300)
Supplement: S4 Table — (PDF) [file pone.0171300.s010.pdf]

S4 Table. Divergent down genes

Gene information		mRNA fold change		H3K4me3 enrichment			H3K27me3 enrichment		
RefSeq gene	gene symbol	iPSCp/sFB-G	mESC/sFB-G	mESC	iPSCp	sFB-G	mESC	iPSCp	sFB-G
NM_009696	Apoe	-4.944	0.495	5394	13	1173	72	70	82
NM_010112	Efs	-4.163	-0.567	3364	276	5266	456	1103	496
NM_138595	Gldc	-4.041	2.063	3884	1043	2583	1525	8019	2891
NM_007806	Cyba	-3.612	-0.875	1758	327	4065	665	609	217
NM_010493	Icam1	-3.415	-0.539	2645	90	2951	506	1864	611
NM_007554	Bmp4	-3.102	1.031	4346	478	1684	1252	1718	3693
NM_198095	Bst2	-2.969	-0.766	2016	3126	4144	101	10	39
NR_003634	Rps4y2	-2.835	2.872	4059	235	1048	65	36	62
NM_007805	Cyb561	-2.805	0.179	4165	632	2528	632	1508	396
NM_145562	9130213B05Rik	-2.574	-0.910	2829	1068	3154	3537	5159	1495
NM_023627	Isyna1	-2.554	0.777	3615	29	1445	369	116	58
NM_172285	Plcg2	-2.546	0.943	4447	1165	4405	5796	5104	3452
NM_175329	Chchd10	-2.545	4.188	4062	57	511	146	148	80
NM_029674	Got1l1	-2.526	1.594	58	17	10	189	489	76
NM_001081642	Xlr4a	-2.501	-0.422	10	12	30	111	342	101
NM_009876	Cdkn1c	-2.499	1.849	1582	271	811	2111	491	977
NM_145402	Tmem51	-2.419	1.771	4679	654	1901	1497	1768	1200
NM_010094	Lefty1	-2.413	0.173	704	19	788	575	149	167
NM_013690	Tek	-2.387	-0.256	955	991	2391	2961	3320	1608
NM_022004	Fxyd6	-2.319	3.245	1343	196	835	1514	1478	1575
NM_013560	Hspb1	-2.297	1.424	2074	515	1840	54	54	45
NM_008816	Pecam1	-2.281	0.744	826	881	2056	2217	2485	1440
NM_172784	Lrp11	-2.279	0.375	5483	1369	4458	902	1924	1086
NM_010882	Ndn	-2.191	1.314	1889	23	458	431	47	60
NM_177630	Ldoc1l	-2.137	-0.602	2167	322	1436	298	391	201

NM_010474	Hs3st1	-2.086	0.043	2615	654	2349	1753	1044	651
NM_026805	Svop	-1.993	-0.005	2082	522	923	3129	6255	1578
NM_018761	Ctnnal1	-1.988	2.351	6132	2029	3198	1040	4576	886
NM_011125	Pltp	-1.972	-0.878	3667	1827	5180	364	1153	585
NM_198724	Egfl7	-1.956	-0.271	3323	1044	4548	413	650	287
NM_133888	Smpdl3b	-1.838	0.537	4785	845	1103	600	1292	330
NM_007707	Socs3	-1.803	1.346	11524	8544	14073	417	74	94
NM_013640	Psmb10	-1.789	-0.824	3247	4187	5797	140	116	55
NM_009663	Alox5ap	-1.742	-0.879	432	360	1439	1509	599	544
NM_023844	Jam2	-1.732	-0.600	7736	2702	5440	1001	1231	2029
NM_011542	Tcea3	-1.712	1.284	2903	609	2993	1644	3149	609
NM_010442	Hmox1	-1.705	-0.665	3834	3617	4060	178	76	119
NM_009143	Sdf2	-1.645	-0.776	7195	4740	5587	234	312	262
NM_007420	Adrb2	-1.641	0.007	3511	1775	4692	506	55	60
NM_008046	Fst	-1.637	-0.702	3115	3054	4909	2024	470	135
NM_026428	Dcxr	-1.637	0.222	3120	1547	3256	251	89	47
NM_001009935	Txnip	-1.630	-0.287	7316	14157	4790	225	250	66
NM_144538	Rab3il1	-1.625	-0.848	1702	3765	8579	1412	1445	202
NM_027185	Def6	-1.617	-0.462	3866	1889	4591	557	1974	652
NM_172729	Nod1	-1.597	0.256	4182	4038	4757	1839	2025	937
NM_027857	Acy3	-1.587	-0.365	203	156	418	111	216	134
NM_026615	Urm1	-1.574	-0.733	4802	4197	4150	954	333	405
NM_001042675	Rbpms	-1.561	1.149	7659	4416	4887	2425	4562	1196
NM_012030	Slc9a3r1	-1.560	-0.690	4225	3194	8262	1241	500	330
NM_172607	Naprt1	-1.548	0.774	1742	755	2955	448	269	127
NR_003642	2900062L11Rik	-1.539	1.001	186	89	228	10	55	25
NM_010356	Gsta3	-1.529	-0.668	212	220	320	595	983	1001
NM_022721	Fzd5	-1.516	1.455	12430	3666	5822	366	262	161
NM_178699	B930041F14Rik	-1.482	-0.009	6950	2249	3129	123	1328	11

NM_176848	Fbxo2	-1.481	1.302	2399	1464	1401	1661	2179	1335
NM_019448	Dnmt3l	-1.428	4.189	3018	171	693	322	1145	503
NM_008378	Impact	-1.421	-0.513	5948	2571	4407	482	613	592
NM_029619	Msrb2	-1.416	-0.390	2168	1599	2467	750	815	617
NM_023065	Ifi30	-1.414	0.393	1784	1877	1776	354	106	117
NM_178195	Hist1h2bf	-1.410	0.040	161	310	273	10	10	10
NM_172145	Fam176b	-1.408	-0.745	3105	2254	3400	109	48	19
NM_030255	Apobec3	-1.393	0.040	1977	474	5145	270	1207	381
NM_013587	Lrpap1	-1.393	0.431	5751	2057	2086	352	944	159
NM_009721	Atp1b1	-1.373	2.438	6289	633	1723	648	830	2107
NM_178198	Hist1h2bj	-1.367	-0.680	233	471	419	11	59	14
NM_011193	Pstpip1	-1.367	-0.541	1531	311	2024	2304	1216	685
NM_028064	Slc39a4	-1.365	2.150	1023	109	515	313	790	154
NM_178200	Hist1h2bm	-1.364	-0.566	423	424	404	38	119	61
NM_007494	Ass1	-1.346	-0.099	3039	1369	3866	1505	2183	1009
NM_025386	Fbxo36	-1.322	-0.501	7828	4572	5674	1314	1392	1296
NM_133663	Itgb4	-1.322	0.344	4292	1071	3217	3950	2615	3205
NM_007925	Eln	-1.315	-0.698	744	398	936	3048	4978	1747
NM_019764	Amotl2	-1.313	-0.828	8575	13733	16503	829	409	298
NM_024229	Pcyt2	-1.297	0.346	3536	1991	3687	291	152	197
NM_025482	Tpd52l2	-1.284	-0.606	4902	2813	4050	242	305	486
NM_173739	Galntl4	-1.268	-0.914	3122	2667	4098	11497	16231	4067
NM_013559	Hsp105	-1.241	-0.642	5169	4151	6307	459	375	444
NM_180962	Cyhr1	-1.230	-0.731	1752	1187	1383	123	83	55
NM_021515	Ak1	-1.228	-0.978	2829	2153	5216	198	75	152
NM_010893	Neu1	-1.206	-0.837	2625	2756	4014	214	99	81
NM_018854	Ift20	-1.202	-0.480	4921	4322	5334	117	110	190
NM_010499	Ier2	-1.193	-0.988	8947	5757	8365	79	64	81
NM_011446	Sox7	-1.191	-0.038	1222	294	2186	1990	1842	1216

NM_028679	Irak3	-1.185	-0.454	4177	4074	3854	2150	3661	1511
NM_022880	Slc29a1	-1.184	-0.086	727	152	880	282	377	112
NM_019949	Ube2l6	-1.164	-0.118	1791	2269	2790	463	217	176
NM_001004150	A4galt	-1.138	-0.854	3579	1798	4408	749	1328	578
NM_054056	Pawr	-1.136	0.942	5650	2432	5605	1991	4297	2289
NM_008326	Irgm1	-1.133	0.060	152	5740	5524	591	214	188
NM_025835	Pccb	-1.133	-0.851	1912	1819	2287	1233	907	868
NM_175665	Hist1h2bk	-1.129	-0.140	460	435	392	12	20	16
NM_009189	Six1	-1.122	-0.481	5059	4660	6489	2252	524	395
NM_007591	Calr	-1.116	0.095	5384	5379	8037	149	175	198
NM_001001181	BC031181	-1.113	-0.454	2809	1795	2955	90	106	51
NM_172702	Serinc2	-1.108	-0.503	2231	738	1986	2069	2842	353
NM_019568	Cxcl14	-1.091	-0.028	3502	280	2266	2037	1203	1681
NM_023635	Rab27a	-1.088	1.871	2397	583	1455	1375	2920	1973
NM_001039533	Pdxdc1	-1.083	-0.959	5328	4420	5840	1364	1650	1766
NM_010395	H2-T10	-1.083	-0.560	72	18	243	178	231	54
NM_194344	Sh3tc1	-1.081	0.494	712	290	1268	1206	2513	707
NM_172498	Ptk2b	-1.078	-0.899	2116	1975	5808	4852	7258	3309
NM_013529	Gfpt2	-1.078	0.662	2948	2455	5763	1714	2956	1228
NM_199018	Stard8	-1.074	0.924	1581	477	1587	824	1348	487
NM_010907	Nfkbia	-1.071	0.184	4084	3282	3595	90	159	58
NM_028544	Rasip1	-1.070	0.230	2436	944	1540	1394	1301	333
NM_133708	Gmppa	-1.066	-0.504	1232	2474	3152	495	274	382
NM_007398	Ada	-1.052	-0.602	1568	1864	4899	1183	818	473
NM_027400	Lman1	-1.045	-0.877	3323	3656	4481	399	714	938
NM_008390	Irf1	-1.029	0.078	5404	5674	6946	527	499	201
NM_178201	Hist1h2bn	-1.027	0.368	387	737	539	46	43	15
NM_013750	Phlda3	-1.025	0.312	7844	9317	10048	544	51	93
NM_008604	Mme	-1.020	0.927	3167	1624	1674	1267	1986	2140

NM_023322	Zkscan14	-1.018	-0.493	1795	2632	3540	104	10	102
NM_026728	Echdc2	-1.016	1.061	1854	1432	1707	415	1474	354
NM_178149	Pik3ip1	-1.015	-0.867	1052	3388	2652	748	354	250
NM_025290	Rsph1	-1.013	2.791	1083	552	1098	553	2981	1258
NM_009657	Aldoc	-1.013	2.286	229	93	105	171	10	101
NM_183170	BC051227	-1.004	0.240	2523	2185	3581	146	64	37
