# Supplementary material for: Identification of the early and late responder genes during the generation of induced pluripotent stem cells from mouse fibroblasts
Source: PLoS One. 2017 Feb 2;12(2):e0171300. doi: 10.1371/journal.pone.0171300 (PMC5289558; doi:10.1371/journal.pone.0171300)
Supplement: S5 Table — (PDF) [file pone.0171300.s011.pdf]

S5 Table. Convergent down genes

Gene information		mRNA fold change		H3K4me3 enrichment			H3K27me3 enrichment		
RefSeq gene	gene symbol	iPSCp/sFB-G	mESC/sFB-G	mESC	iPSCp	sFB-G	mESC	iPSCp	sFB-G
NM_010514	Igf2	-6.278	-1.248	1218	80	1380	2251	1166	814
NM_019521	Gas6	-6.237	-6.290	2429	1422	10339	1686	3399	1046
NM_173749	E430002G05Rik	-5.859	-5.648	367	523	5070	3817	4280	3920
NM_019696	Cpxm1	-5.756	-3.946	1640	215	2778	629	358	359
NM_054077	Prelp	-5.542	-7.650	167	110	4011	965	699	269
NM_009776	Serping1	-5.538	-4.761	314	317	3178	435	1187	365
NM_008161	Gpx3	-5.491	-4.895	1318	228	7400	378	797	167
NM_010216	Figf	-5.405	-6.554	408	895	2928	302	468	337
NM_009778	C3	-5.388	-5.229	383	363	510	2212	3496	700
NM_176922	Itga11	-5.212	-6.183	1851	1734	8712	8148	5451	2820
NM_022814	Svep1	-5.115	-5.231	2750	3263	6806	7526	5336	3560
NM_019759	Dpt	-5.019	-6.467	167	610	5322	1178	880	952
NM_007876	Dpep1	-4.947	-5.139	228	190	1996	783	1167	702
NM_001083895	Srpx2	-4.851	-6.796	60	583	1992	600	793	377
NM_015814	Dkk3	-4.837	-3.868	1214	615	3038	2426	2954	771
NM_008489	Lbp	-4.563	-4.783	251	168	2709	1271	2399	769
NM_016873	Wisp2	-4.519	-7.600	168	996	8713	629	275	362
NM_011150	Lgals3bp	-4.514	-7.939	203	1406	4056	719	192	269
NM_008607	Mmp13	-4.471	-5.012	18	31	143	386	207	443
NM_008760	Ogn	-4.432	-5.762	26	768	1445	378	277	549
NM_010501	Ifit3	-4.392	-6.149	16	577	350	264	215	140
NM_026864	Rasl11a	-4.327	-3.809	2482	1000	5432	556	173	109
NM_009675	Aoc3	-4.309	-4.254	286	376	817	345	451	229
NM_007801	Ctsh	-4.238	-5.774	1915	1829	3573	1497	699	488
NM_029568	Mfap4	-4.150	-3.773	229	126	242	393	213	185

NM_175643	Adamts2	-4.067	-7.318	7405	3495	12272	16226	7612	8050
NM_008509	Lpl	-4.059	-5.863	1213	3277	7039	1816	734	643
NM_021719	Cldn15	-3.985	-4.369	146	260	2504	349	409	190
NM_016911	Srpx	-3.934	-4.874	251	996	2043	1463	1224	1210
NM_008185	Gstt1	-3.929	-3.238	503	806	2813	916	1506	503
NM_010330	Emb	-3.908	-2.177	3361	1594	4599	1032	1398	1532
NM_015744	Enpp2	-3.872	-3.659	723	1048	1177	3519	4226	4439
NM_019498	Olfm1	-3.871	-3.757	2224	584	3079	2248	2161	515
NM_019946	Mgst1	-3.838	-5.498	345	1204	4794	685	655	441
NM_021342	Kcne4	-3.792	-4.427	26	1203	5776	334	74	81
NM_133903	Spon2	-3.775	-2.886	362	103	204	366	501	41
NM_008610	Mmp2	-3.746	-5.580	1571	1985	6620	1682	649	736
NM_013834	Sfrp1	-3.708	-2.694	3402	1025	6898	2427	2464	606
NM_009780	C4b	-3.644	-3.469	179	285	259	1018	1366	537
NM_008597	Mgp	-3.597	-5.511	68	1117	1480	225	264	87
NM_008059	G0s2	-3.533	-3.931	959	447	3346	207	315	83
NM_010517	Igfbp4	-3.494	-3.800	1677	1271	4217	2149	2175	473
NM_194334	Tbc1d2b	-3.390	-4.926	3662	4565	7652	1954	1531	1489
NM_017370	Hp	-3.366	-3.426	19	36	239	280	265	102
NM_016847	Avpr1a	-3.359	-5.177	1038	4932	9692	1184	795	289
NM_008546	Mfap2	-3.339	-3.144	299	358	1522	271	1036	143
NM_009627	Adm	-3.335	-3.514	880	5074	5883	1279	337	37
NM_029803	Ifi27	-3.307	-3.885	10	184	203	99	205	47
NM_026235	Larp6	-3.287	-4.072	1734	991	4621	1345	1268	494
NM_134102	Pla1a	-3.281	-3.519	609	552	2378	1462	1091	891
NM_019444	Ramp2	-3.279	-2.966	1220	538	2406	1011	1277	495
NM_153107	Cpz	-3.275	-2.345	1585	295	1737	2419	3851	1105
NM_008008	Fgf7	-3.266	-5.171	185	7156	9511	1732	911	1325
NM_008987	Ptx3	-3.256	-5.185	1826	3767	3962	1044	428	252

NM_013467	Aldh1a1	-3.254	-3.145	100	367	523	1664	1412	1727
NM_181988	Rerg	-3.238	-3.002	1380	2144	3402	5371	5938	3803
NM_026439	Ccdc80	-3.220	-6.496	238	5661	13444	1424	876	1090
NM_021472	Ang	-3.216	-6.748	1964	3077	4007	521	500	343
NM_027852	Rarres2	-3.207	-3.828	14	218	382	130	209	82
NM_010809	Mmp3	-3.194	-5.940	40	86	459	333	225	299
NM_011819	Gdf15	-3.096	-3.469	1337	535	4223	222	137	41
NM_146162	Tmem119	-3.080	-4.334	230	1079	3067	767	480	68
NM_145467	Itgbl1	-3.053	-4.566	597	944	1379	5587	3347	10093
NM_053195	Slc24a3	-3.034	-5.190	1210	1846	3224	12117	11122	14579
NM_030150	Dhx58	-2.959	-2.104	249	317	1132	575	116	195
NM_010582	Itih2	-2.951	-3.249	210	265	699	1833	1220	1004
NM_133859	Olfml3	-2.943	-6.336	113	2759	2197	240	107	41
NM_009994	Cyp1b1	-2.927	-4.530	1833	1811	6498	2742	2925	895
NM_009841	Cd14	-2.922	-3.897	852	1549	3876	681	108	65
NM_172119	Dio3	-2.905	-2.207	1466	91	1384	1807	226	472
NM_030206	Cygb	-2.894	-4.431	1719	1578	5630	2469	1471	348
NM_133792	Lypla3	-2.892	-2.029	3100	3091	6115	243	360	550
NM_001001183	---	-2.881	-3.214	239	356	3664	814	895	661
NM_008529	Ly6e	-2.844	-2.875	1905	2948	4473	362	293	138
NM_008592	Foxc1	-2.841	-4.255	3033	6448	11575	2885	189	115
NM_008813	Enpp1	-2.829	-3.916	3047	2128	3730	3400	2369	2412
NM_008788	Pcolce	-2.818	-4.170	759	4511	7548	157	222	150
NM_010145	Ephx1	-2.804	-3.575	1696	1909	3134	1353	850	644
NM_176073	Pgcp	-2.765	-4.615	1433	3894	4807	10018	10857	12776
NM_144945	Lgi2	-2.762	-2.695	2064	1121	1088	2407	2670	845
NM_008176	Cxcl1	-2.748	-5.795	114	826	921	136	35	28
NM_207298	Cercam	-2.747	-1.047	3313	986	3659	935	427	344
NM_181326	Zfp521	-2.725	-3.982	1609	1220	1607	6732	4962	4776

NM_033314	Slco2a1	-2.722	-2.391	2519	563	3709	3895	3177	1679
NM_207655	Egfr	-2.683	-3.975	5946	5397	13626	9499	4542	8734
NM_020581	Angptl4	-2.672	-1.618	2047	1618	3698	269	702	137
NM_207229	Plac9	-2.656	-2.057	10	12	30	30	146	10
NM_010738	Ly6a	-2.630	-6.702	35	1631	1557	94	216	80
NM_013743	Pdk4	-2.620	-1.408	2257	1224	2675	398	513	473
NM_144799	Lmcd1	-2.601	-3.268	490	922	4212	2958	3520	1355
NM_013654	Ccl7	-2.588	-6.396	38	3028	2050	202	73	69
NM_011985	Mmp23	-2.579	-4.112	1103	483	2275	362	42	65
NM_011854	Oasl2	-2.559	-3.448	155	1315	1166	455	665	292
NM_054042	Cd248	-2.555	-4.931	1265	2903	6385	1185	389	231
NM_008458	Serpina3c	-2.546	-2.658	86	99	220	326	522	212
NM_020510	Fzd2	-2.531	-3.871	4000	6515	9035	1729	196	421
NM_145515	Mark1	-2.523	-2.895	2726	2311	4457	3543	3881	2617
NM_010189	Fcgrt	-2.520	-3.801	211	1427	1765	138	263	78
NM_013839	Nr1h3	-2.495	-2.745	443	223	1908	508	177	159
NM_009373	Tgm2	-2.494	-2.119	806	514	3209	2137	1143	503
NM_025797	Cyb5	-2.490	-2.434	3170	3863	5730	582	1304	701
NM_008968	Ptgis	-2.484	-2.435	2537	838	4365	2189	4105	1084
NM_029631	Abhd14b	-2.483	-3.206	891	1722	2924	257	37	105
NM_011254	Rbp1	-2.478	-1.396	890	626	2575	2126	1558	530
NM_009142	Cx3cl1	-2.476	-2.709	773	580	2312	1382	1349	278
NM_018738	Igtp	-2.436	-2.752	397	1792	1691	941	260	264
NM_008144	Bscl2	-2.428	-1.393	1054	649	1287	226	78	257
NM_010791	Meox1	-2.420	-2.100	589	388	747	2529	2353	764
NM_153145	Abca8a	-2.368	-2.527	178	370	649	2310	2621	2752
NM_146007	Col6a2	-2.358	-6.231	590	3226	6697	2200	761	1001
NM_010495	Id1	-2.356	-1.474	1474	3227	5150	593	59	57
NM_153790	Scarf2	-2.355	-4.379	4466	3619	6994	6606	1739	768

NM_031397	Bicc1	-2.350	-4.908	4773	6084	10593	9618	7308	5798
NM_009378	Thbd	-2.343	-2.486	708	761	5785	1163	282	63
NM_021474	Efemp2	-2.342	-5.462	994	3289	6460	698	340	253
NM_026316	Aldh3b1	-2.338	-3.147	993	1293	2446	717	595	289
NM_010180	Fbln1	-2.338	-2.822	2343	2433	4509	3321	3956	2525
NM_007825	Cyp7b1	-2.334	-2.526	1420	1729	1777	4788	3845	3412
NM_145379	Mrgprf	-2.329	-5.206	335	1741	3326	631	199	117
NM_010728	Lox	-2.322	-7.557	2063	5711	10692	1867	569	1141
NM_027878	1200002N14Rik	-2.316	-4.623	1377	3692	3857	2561	1546	1103
NM_009252	Serpina3n	-2.314	-4.580	101	86	547	605	159	51
NM_010764	Man2b1	-2.308	-1.925	2653	1802	3266	378	388	416
NM_176830	1110036O03Rik	-2.305	-3.500	2760	4657	8460	1446	293	196
NM_010222	Fkbp7	-2.290	-3.819	181	2683	2187	341	291	276
NM_023699	Nfatc4	-2.265	-1.866	1669	2198	6153	869	1099	400
NM_008416	Junb	-2.250	-3.171	7372	5653	11372	202	88	48
NM_019861	Ctsf	-2.247	-1.617	883	1075	2594	572	716	229
NM_011435	Sod3	-2.244	-2.695	974	249	1261	1232	498	81
NM_010708	Lgals9	-2.238	-2.842	1180	2124	4510	896	1622	575
NM_008369	Il3ra	-2.209	-2.789	2268	2177	2374	422	372	273
NM_010906	Nfix	-2.207	-5.676	6547	7244	19711	20904	2933	2462
NM_009099	Trim30	-2.193	-2.341	116	594	612	925	540	402
NM_009930	Col3a1	-2.189	-4.348	120	8263	5764	1051	557	1025
NM_008640	Laptm4a	-2.188	-1.039	4019	6044	3090	328	364	272
NM_011909	Usp18	-2.187	-4.447	714	5053	2362	2012	921	726
NM_010741	Ly6c1	-2.183	-3.732	20	144	124	110	186	122
NM_019449	Unc93b1	-2.172	-2.527	1837	2962	4373	1071	562	277
NM_175168	Ptk7	-2.160	-1.302	6489	2365	7316	2142	8202	1500
NM_007802	Ctsk	-2.158	-5.848	56	1641	1468	308	310	197
NM_011333	Ccl2	-2.147	-4.148	23	1162	501	205	170	57

NM_009255	Serpine2	-2.147	-3.015	2623	3241	4414	2380	1979	1831
NM_029758	D12Ertd553e	-2.146	-1.363	909	668	842	2980	2975	1641
NM_009325	Tbxa2r	-2.141	-1.978	1785	176	1578	924	429	109
NM_008764	Tnfrsf11b	-2.140	-4.946	215	1049	1370	1037	1333	1231
NM_145158	Emilin2	-2.137	-4.713	2841	3085	4111	4343	4917	1847
NM_007987	Fas	-2.136	-2.503	353	1921	2902	1802	1079	1380
NM_019922	Crtap	-2.099	-2.341	3796	2715	4053	270	292	209
NM_146041	Gmds	-2.095	-2.303	3944	6429	8533	13787	13592	16811
NM_145978	Pdlim2	-2.093	-2.723	1545	2496	4901	1863	261	559
NM_020561	Smpdl3a	-2.091	-4.299	862	1564	1205	1204	422	529
NM_030209	Crispld2	-2.086	-2.287	1256	369	3311	3933	1773	3163
NM_009641	Angpt4	-2.066	-3.479	704	887	2679	2261	736	977
NM_030127	Htra3	-2.063	-2.896	2516	531	1912	2832	1308	807
NM_134090	Kdelr3	-2.059	-2.237	2345	4877	7746	811	1052	320
NM_130451	Slc2a10	-2.046	-2.995	533	1405	3500	1536	986	219
NM_173743	BC020489	-2.033	-3.059	14	35	30	156	212	40
NM_008885	Pmp22	-2.033	-3.863	2131	5293	10179	3825	850	803
NM_172294	Sulf1	-2.032	-3.823	1362	1953	3804	5287	4386	3955
NM_009636	Aebp1	-2.017	-6.142	1494	4769	8027	1683	203	263
NM_030749	Sil1	-2.012	-1.989	3940	3423	3840	8345	5922	7205
NM_019656	Tspan6	-2.004	-3.352	579	799	1608	225	90	86
NM_010511	Ifngr1	-1.998	-3.625	1602	3207	4027	834	590	557
NM_008183	Gstm2	-1.985	-1.581	84	499	660	211	645	45
NM_011838	Lynx1	-1.983	-2.821	862	1048	2655	752	419	244
NM_011406	Slc8a1	-1.967	-2.494	3195	5327	6784	12324	12196	14290
NM_018734	Gbp3	-1.965	-3.561	145	824	772	467	210	294
NM_026960	Gsdmd	-1.963	-1.887	249	650	2199	442	582	145
NM_019959	C1qtnf1	-1.957	-2.365	381	188	292	1343	965	341
NM_024249	---	-1.956	-2.407	3808	4877	6936	1363	1104	1301

NM_021528	Chst12	-1.954	-2.258	2422	3558	6735	548	567	309
NM_009144	Sfrp2	-1.952	-1.974	1624	191	2515	1707	706	602
NM_173047	Cbr3	-1.952	-1.519	1628	2676	4063	1251	945	626
NM_145394	Slc44a3	-1.949	-1.115	1607	1463	2764	3166	3963	984
NM_008675	Nbl1	-1.943	-3.603	1604	1928	3964	2112	904	223
NM_011427	Snai1	-1.933	-2.319	1021	4440	11302	1448	113	50
NM_007743	Col1a2	-1.930	-4.666	450	10866	8000	2374	803	1380
NM_028181	Ccpg1	-1.926	-2.197	3886	2703	3830	647	687	795
NM_153573	Fkbp14	-1.922	-3.340	437	3398	3140	539	608	356
NM_053108	Glrx	-1.910	-3.015	395	2335	3373	344	179	225
NM_026772	Cdc42ep2	-1.903	-2.752	45	20	45	65	84	48
NM_175149	2310022B05Rik	-1.876	-3.196	2227	4923	7583	834	544	603
NM_139307	Vasn	-1.871	-3.329	1480	3748	11079	519	338	253
NM_028778	Nuak2	-1.865	-2.121	1761	2545	4062	1616	1748	505
NM_011756	Zfp36	-1.864	-2.495	3876	6613	5294	146	42	43
NM_008483	Lamb2	-1.847	-2.971	2360	3483	5383	451	280	195
NM_201641	Ugt1a9	-1.842	-5.080	1232	2475	2689	7762	4842	7170
NM_173371	H6pd	-1.841	-3.265	2588	3106	5071	1517	822	468
NM_011173	Pros1	-1.839	-3.685	1877	3850	4403	1867	1482	1686
NM_013534	Leprel2	-1.836	-3.569	2277	2978	3537	1064	2476	318
NM_025360	Tmed3	-1.828	-3.727	1108	2514	3322	330	214	165
NM_009318	Tapbp	-1.822	-1.660	3372	6701	5800	350	444	133
NM_028351	Rspo3	-1.818	-2.172	1390	1943	4634	3341	2382	4824
NM_033602	Peli2	-1.817	-2.397	4447	6031	8542	5342	3552	3313
NM_197999	2210023G05Rik	-1.816	-2.203	20	47	82	312	257	200
NM_022415	Ptges	-1.804	-1.971	540	511	4966	688	852	246
NM_015786	Hist1h1c	-1.800	-4.720	1788	1890	2856	45	32	40
NM_011723	Xdh	-1.780	-4.549	433	3561	4513	2991	2402	2018
NM_011594	Timp2	-1.779	-5.168	3041	6807	8490	4346	1434	1060

NM_012043	Islr	-1.774	-1.758	167	124	4082	613	268	177
NM_011530	Tap2	-1.770	-1.852	1439	2391	3976	1250	970	254
NM_023738	Ube1l	-1.767	-3.997	283	1346	2551	869	228	312
NM_130449	Colec12	-1.766	-2.460	3408	3621	6109	4883	2742	4228
NM_027455	Qpct	-1.759	-3.407	3453	2539	2992	2078	1588	1664
NM_029537	Tmem98	-1.755	-2.587	1312	1249	2287	923	923	297
NM_008321	Id3	-1.750	-1.172	6082	3894	4156	942	17	29
NM_009976	Cst3	-1.729	-2.351	3749	5452	5078	142	92	71
NM_028472	Bmper	-1.728	-3.217	3986	3211	6079	9328	7388	8727
NM_178697	Clca5	-1.727	-1.919	141	149	247	861	1115	411
NM_023056	Tmem176b	-1.725	-5.395	171	2319	4129	823	393	128
NM_013792	Naglu	-1.722	-2.886	3012	2583	3838	1567	136	201
NM_009062	Rgs4	-1.719	-1.694	82	202	956	485	355	476
NM_019978	Dclk1	-1.719	-2.724	2194	4491	5059	12514	7611	4404
NM_019631	Tmem45a	-1.716	-2.271	993	2645	4168	2227	1446	2274
NM_026555	Rcn3	-1.704	-1.663	830	2657	1947	266	317	145
NM_008520	Ltbp3	-1.698	-4.011	3394	3564	5696	3989	499	365
NM_011416	Smarca2	-1.696	-3.584	7359	9866	13087	9517	5064	4875
NM_178600	Vkorc1	-1.689	-1.520	1976	1908	1993	80	130	30
NM_199307	Ece1	-1.689	-1.622	4548	3901	7011	4333	6451	1032
NM_134154	Slc25a45	-1.682	-2.718	178	305	1826	345	180	126
NM_011341	Sdf4	-1.677	-2.347	6963	4488	3212	451	420	177
NM_001001488	Atp8b1	-1.662	-4.183	3262	5180	8409	6609	3838	3462
NM_033354	Sec16b	-1.658	-2.649	559	1148	1781	2943	2353	1495
NM_021384	Rsad2	-1.656	-2.337	130	377	420	573	501	297
NM_019456	Apbb1ip	-1.653	-3.692	661	4037	5043	4584	1967	2415
NM_024198	Gpx7	-1.639	-2.322	2208	2587	2177	409	667	213
NM_018750	Rassf5	-1.625	-3.135	4439	3409	7140	5360	3846	1573
NM_022325	Ctsz	-1.622	-2.865	1917	2611	3460	261	194	186

NM_010551	Il16	-1.617	-1.746	649	463	882	3493	3164	1267
NM_019877	Copz2	-1.614	-2.369	1389	2873	4960	1031	477	279
NM_007833	Dcn	-1.613	-5.174	79	1048	611	901	766	1171
NM_012032	Serinc3	-1.611	-1.155	2109	3889	3275	262	366	438
NM_009120	Sar1a	-1.609	-2.422	2563	5364	4715	315	471	400
NM_008809	Pdgfrb	-1.609	-3.891	793	7089	11612	4213	1340	1947
NM_133718	Tmem30a	-1.602	-1.896	3555	3855	4206	480	564	612
NM_028841	Tspan17	-1.594	-1.820	1119	1504	2824	626	267	145
NM_026405	Rab32	-1.579	-4.560	2057	3403	3799	1020	394	443
NM_027870	Armcx3	-1.576	-3.286	471	2128	2353	202	57	34
NM_010221	Fkbp10	-1.570	-3.171	1387	3073	6349	1402	589	203
NM_175274	Ttyh3	-1.562	-4.177	1870	4188	7875	1481	781	620
NM_001042489	Hvcn1	-1.559	-1.055	792	726	1377	663	1978	457
NM_146136	Slc16a4	-1.553	-1.805	42	178	129	287	544	252
NM_025429	Serpinb1a	-1.552	-2.266	88	318	493	307	321	315
NM_010421	Hexa	-1.551	-1.923	3061	5003	5475	696	418	382
NM_024263	Mxra8	-1.542	-3.894	2810	5085	3826	869	272	63
NM_029600	Abcc3	-1.535	-2.202	1024	993	1973	5475	4654	2017
NM_054041	Antxr1	-1.533	-3.914	2759	7919	9030	9226	7740	4406
NM_153782	---	-1.531	-4.251	1789	5589	8064	4408	1454	1294
NM_178939	Pdrg1	-1.528	-2.167	3887	2656	4656	220	226	273
NM_008047	Fstl1	-1.527	-3.144	2275	5441	6638	1574	2706	1372
NM_153513	BC028528	-1.527	-1.813	207	655	634	189	461	121
NM_009283	Stat1	-1.524	-1.904	2647	4324	4459	2111	908	994
NM_013819	H2-M3	-1.521	-2.010	975	1434	2887	584	131	139
NM_008967	Ptgir	-1.518	-2.153	1194	81	1085	850	140	38
NM_010924	Nnmt	-1.505	-5.378	146	2098	3208	703	317	270
NM_011030	P4ha1	-1.500	-2.488	1823	5504	4913	1381	1376	1334
NM_013549	Hist2h2aa1	-1.499	-3.149	106	268	162	17	10	10

NM_144786	Ggtl3	-1.499	-3.016	565	1164	2994	1618	1189	723
NM_010580	Itgb5	-1.486	-2.833	5869	6326	8152	3281	2949	3602
NM_001037865	Col28a1	-1.484	-3.456	548	2637	1365	5706	5786	6008
NM_133733	9030425E11Rik	-1.484	-1.259	4796	3419	7923	4995	4938	3020
NM_008330	Ifi47	-1.479	-2.005	300	234	339	841	420	226
NM_021606	Nek6	-1.479	-2.181	4165	4711	5919	2318	1610	1329
NM_013683	Tap1	-1.471	-1.141	2577	2170	5966	1897	1044	225
NM_201367	Gpr176	-1.467	-5.358	2458	3442	5907	5815	3514	2528
NM_007855	Twist2	-1.466	-4.364	2797	6582	13177	4611	2941	2057
NM_010934	Npy1r	-1.461	-2.074	148	709	1140	954	684	364
NM_181277	Col14a1	-1.458	-1.539	2120	3767	4409	11015	8904	8037
NM_013642	Dusp1	-1.457	-1.017	8876	8710	9633	339	238	103
NM_146120	Gsn	-1.456	-1.477	839	284	809	2036	533	539
NM_175425	C1qtnf7	-1.455	-1.519	40	145	49	537	516	376
NM_007874	Reep5	-1.455	-4.364	4013	5362	5823	1162	520	658
NM_010212	Fhl2	-1.454	-3.921	1789	4175	5706	2999	1309	1178
NM_010479	Hspa1a	-1.450	-2.298	164	342	119	275	84	41
NM_010581	Cd47	-1.448	-4.303	3537	7841	7237	2026	1362	1282
NM_026160	Map1lc3b	-1.447	-1.887	3186	3660	5422	200	206	214
NM_173008	A430110N23Rik	-1.446	-1.797	348	541	2362	894	557	285
NM_194069	---	-1.432	-5.186	58	1933	1020	406	273	57
NM_013646	Rora	-1.427	-2.286	12187	13177	21347	28251	18532	17250
NM_175401	Fbxw17	-1.421	-1.722	2524	2943	3125	239	308	398
NM_008175	Grn	-1.419	-1.510	3589	3559	5719	299	136	122
NM_145418	BC013529	-1.414	-3.023	2265	2860	3357	477	407	383
NM_007547	Sirpa	-1.403	-3.216	3707	3595	7827	2669	893	854
NM_010095	Ebf2	-1.402	-1.143	2843	2398	6323	13568	14410	12175
NM_023386	Rtp4	-1.400	-1.932	72	2612	2149	336	119	166
NM_016744	Pde1a	-1.400	-3.275	693	2615	5414	7326	5420	9034

NM_010729	Loxl1	-1.392	-5.350	3194	9328	10920	2036	864	1068
NM_007656	Cd82	-1.391	-2.233	1595	1232	2991	3881	1247	1051
NM_031257	Plekha2	-1.391	-1.151	2302	4473	5146	2172	2383	950
NM_025760	Ptplad2	-1.390	-1.999	151	862	974	684	545	415
NM_029935	4631426J05Rik	-1.384	-1.551	5723	2486	5413	5822	3845	981
NM_021460	Lipa	-1.381	-4.264	1274	3669	3755	844	805	810
NM_010424	Hfe	-1.379	-2.033	127	1260	1340	777	296	265
NM_016850	Irf7	-1.376	-1.843	128	1400	1020	175	84	22
NM_021434	Gpr180	-1.373	-1.773	2167	2277	4057	818	444	514
NM_011512	Surf4	-1.369	-2.257	4258	5248	6027	275	476	423
NM_027519	6330406I15Rik	-1.364	-4.332	831	6551	8063	1920	1125	433
NM_010376	H13	-1.361	-1.623	6853	6893	10058	979	754	705
NM_007736	Col4a5	-1.358	-3.828	300	1921	1942	2625	2776	2406
NM_144875	Rab7l1	-1.348	-2.122	1974	2004	3647	258	301	152
NM_133918	Emilin1	-1.343	-2.775	1122	3541	6016	413	252	66
NM_013846	Ror2	-1.340	-1.180	6849	3703	8327	8074	7114	6387
NM_019802	Ggcx	-1.333	-3.772	2086	4738	3213	443	686	263
NM_080456	Mrps6	-1.329	-2.831	4233	4961	6884	1558	1231	1479
NM_026353	4930570C03Rik	-1.325	-2.957	2427	3091	3953	549	340	182
NM_010234	Fos	-1.320	-1.608	4823	6095	4430	864	129	103
NM_134065	Epdr1	-1.319	-5.084	2374	3647	4214	1059	487	767
NM_009933	Col6a1	-1.317	-6.821	325	5015	6205	1012	624	558
NM_139300	Mylk	-1.315	-1.388	2002	1712	2334	11455	10352	8874
NM_020559	Alas1	-1.310	-2.131	2662	2742	4237	611	132	267
NM_009712	Arsb	-1.308	-4.607	2233	3953	4809	7748	4058	4320
NM_053139	Pcdhb14	-1.304	-2.057	81	224	370	676	100	181
NM_009713	Arsa	-1.295	-2.144	2192	3400	4089	451	130	162
NM_145466	BC006662	-1.294	-1.489	2580	2027	3048	886	551	729
NM_172416	Ostm1	-1.290	-1.974	4326	4235	4893	539	563	741

NM_153560	C230093N12Rik	-1.287	-1.289	4309	4755	6684	1127	486	683
NM_028862	Rnf145	-1.279	-1.857	4511	5980	7202	1235	1085	1405
NM_007730	Col12a1	-1.277	-5.317	1055	3512	6704	4473	2264	3107
NM_013673	Sp100	-1.274	-1.015	282	476	484	1722	1287	1288
NM_010336	Edg2	-1.273	-2.627	3793	5475	4820	3934	2642	2113
NM_010332	Ednra	-1.273	-5.652	952	2104	4113	2315	1573	1716
NM_025378	Ifitm3	-1.268	-1.726	990	1974	1672	32	72	13
NM_198626	AI480653	-1.267	-1.179	5565	3194	4743	3941	3956	3199
NM_021507	Sqrdl	-1.267	-2.468	71	112	215	944	430	660
NM_026908	Cab39l	-1.266	-1.216	3328	3875	6035	2309	2484	2820
NM_011817	Gadd45g	-1.261	-2.073	6805	7520	10187	569	60	81
NM_013606	Mx2	-1.248	-2.283	200	749	802	993	831	575
NM_026174	Entpd4	-1.248	-2.115	3132	2902	3341	3059	869	1250
NM_010851	Myd88	-1.243	-2.231	1243	3592	4645	399	166	95
NM_007898	Ebp	-1.241	-1.325	1522	1913	2585	78	49	55
NM_025295	Btd	-1.238	-1.950	3307	3888	3815	1139	634	656
NM_025827	Lonp2	-1.236	-1.497	3069	4187	6982	1444	1870	2740
NM_007471	App	-1.234	-4.807	8267	8254	11354	9070	4835	5504
NM_177614	Os9	-1.234	-3.432	3201	4212	3995	1129	895	638
NM_174995	Mgst2	-1.232	-2.096	238	225	567	1232	674	288
NM_009074	Mst1r	-1.231	-1.298	1060	308	3153	1777	1526	627
NM_172893	Parp12	-1.230	-1.315	4211	6547	6472	2007	1606	856
NM_018869	Grk5	-1.229	-2.139	4751	4672	6892	6212	4741	4577
NM_178406	Gpr153	-1.227	-2.760	3108	2340	2358	2661	451	514
NM_010560	Il6st	-1.221	-1.329	4587	8406	6836	791	1148	1042
NM_027320	Ifi35	-1.216	-1.576	179	1697	1957	298	174	250
NM_008850	Pitpna	-1.216	-1.163	5235	3246	5562	1113	796	866
NM_028375	Cxx1c	-1.215	-2.066	72	976	1681	84	10	24
NM_178141	3830406C13Rik	-1.208	-2.320	2056	2458	3201	606	550	610

NM_016846	Rgl1	-1.203	-3.367	2246	5116	6922	6445	3044	2733
NM_175400	Sephs1	-1.197	-1.574	4043	3353	4502	449	533	517
NM_027127	2310016C16Rik	-1.195	-3.778	136	4021	2493	464	146	226
NM_177260	Tmem154	-1.192	-1.570	361	404	913	2346	1424	668
NM_009890	Ch25h	-1.191	-4.470	718	3630	4105	762	342	197
NM_009382	Thy1	-1.190	-5.917	657	4438	7658	1132	415	143
NM_147220	Abca9	-1.185	-1.246	308	690	1142	2595	1558	2625
NM_023422	Hist1h2bc	-1.185	-3.375	574	3995	2901	189	221	285
NM_013796	Nagpa	-1.185	-1.855	3817	2235	3892	420	186	176
NM_173006	Pon3	-1.179	-1.950	296	1591	1851	1159	834	918
NM_176996	Smo	-1.178	-2.010	3501	4007	6769	2724	3027	1071
NM_025785	Fbxo25	-1.177	-1.549	3197	3155	5531	869	1141	1212
NM_025516	Ergic3	-1.177	-1.707	3380	2516	3304	295	319	286
NM_011962	Plod3	-1.173	-2.977	3301	4325	5885	363	208	181
NM_029364	Gns	-1.173	-4.064	3698	7220	5005	1083	1002	745
NM_025661	Ormdl3	-1.173	-1.701	2223	2951	3583	250	305	294
NM_153778	Atoh8	-1.171	-1.175	3396	1033	9352	5476	12997	770
NM_008150	Gpc4	-1.170	-1.934	4045	3642	5175	1491	1343	1538
NM_016678	Reck	-1.169	-1.763	3923	2880	2948	2887	1797	983
NM_133967	Zdhhc7	-1.167	-1.827	3701	4284	8397	751	487	501
NM_010422	Hexb	-1.159	-1.293	3646	2413	2857	800	611	633
NM_007993	Fbn1	-1.155	-5.092	3284	7252	13263	7540	6263	4398
NM_026436	Tmem86a	-1.146	-2.782	1629	2921	1936	604	129	25
NM_172604	Scara3	-1.145	-2.976	1278	661	1305	2819	2719	3685
NM_013476	Ar	-1.143	-2.111	759	3336	5762	3445	2128	2187
NM_027868	Slc41a3	-1.140	-1.487	1416	2622	2311	2661	5742	1184
NM_027122	Mfsd3	-1.136	-1.327	2232	1731	3092	117	65	77
NM_022813	Scamp2	-1.133	-1.031	2141	2519	3545	694	511	508
NM_027817	Grap	-1.125	-1.039	651	289	1116	2636	1563	616

NM_178762	A930025D01Rik	-1.120	-1.440	4485	4075	5125	555	109	198
NM_009883	Cebpb	-1.118	-2.984	5640	4370	5017	235	121	37
NM_025858	Scotin	-1.114	-2.781	2634	4439	6896	479	326	193
NM_016923	Ly96	-1.113	-3.411	329	2773	2065	841	713	622
NM_026554	Ncbp2	-1.112	-1.044	6035	4932	5488	163	266	341
NM_026721	Slc39a13	-1.110	-3.108	2856	4088	5878	305	194	207
NM_010427	Hgf	-1.109	-1.960	118	2064	2267	1894	1541	917
NM_010684	Lamp1	-1.102	-2.729	4652	6005	7123	515	653	661
NM_008719	Npas2	-1.101	-1.190	4373	2565	6230	10630	6700	4787
NM_011693	Vcam1	-1.095	-4.147	105	4792	1824	836	348	363
NM_011957	Creb3l1	-1.094	-1.566	3106	4115	7886	3340	2581	956
NM_177068	Olfml2b	-1.094	-4.403	1533	3735	3399	2703	1035	1342
NM_027995	Paqr7	-1.092	-1.854	911	1475	1400	919	423	52
NM_001024955	Pik3r1	-1.092	-1.891	216	212	237	270	386	298
NM_020557	Tyki	-1.091	-1.520	2308	1525	1510	764	535	435
NM_009761	Bnip3l	-1.090	-1.221	3028	2999	3079	332	564	552
NM_177909	Slc9a9	-1.087	-1.667	2702	3939	6061	18532	14712	15505
NM_009744	Bcl6	-1.087	-1.828	2653	7691	7692	1933	351	535
NM_022883	Lpin3	-1.086	-1.224	1548	869	1107	1069	1028	545
NM_145367	Txndc5	-1.084	-2.554	2928	4774	5528	1007	765	677
NM_018830	Asah2	-1.083	-2.842	675	945	746	3248	3036	2785
NM_032003	Enpp5	-1.083	-2.414	453	1633	1402	439	405	387
NM_178413	Thnsl2	-1.082	-1.652	933	2652	2334	1033	1050	422
NM_030262	Pofut2	-1.081	-1.367	3884	4321	5781	349	225	310
NM_029153	Scamp1	-1.080	-1.105	4460	4142	5507	2213	1897	1953
NM_080555	Ppap2b	-1.079	-2.288	7139	10921	8567	2145	1695	937
NM_139229	Cog8	-1.078	-1.188	13575	9097	13257	294	250	383
NM_001004363	Nuak1	-1.078	-1.264	5220	7512	10694	2736	2050	1413
NM_173751	Ilvbl	-1.073	-2.962	2938	2708	3404	443	224	271

NM_025828	Lman2	-1.072	-1.428	5231	4338	5269	562	567	397
NM_008410	Itm2b	-1.067	-3.417	3336	5457	6434	643	380	570
NM_026178	Mmd	-1.066	-2.208	1952	3200	3617	2189	823	733
NM_130796	Snag1	-1.066	-1.412	5901	10242	16261	1453	492	477
NM_028777	Sec14l1	-1.066	-1.645	4727	4521	6559	1578	1239	763
NM_010150	Nr2f6	-1.062	-1.185	5514	5986	10454	1321	246	201
NM_008606	Mmp11	-1.059	-2.562	1687	6418	5258	712	359	274
NM_009058	Ralgds	-1.058	-1.097	5736	5929	9328	1402	1126	931
NM_146211	Glt25d1	-1.057	-1.676	4296	3792	5462	525	418	274
NM_144916	Tmem150	-1.056	-1.506	1707	3261	3536	291	215	135
NM_010549	Il11ra2	-1.056	-2.707	90	726	724	145	92	29
NM_172769	Sc5d	-1.051	-1.480	2031	3747	4024	375	248	318
NM_177322	Agtr1a	-1.045	-1.634	374	987	863	2005	1929	3234
NM_145925	Pttg1ip	-1.044	-1.331	3543	2593	3844	482	455	400
NM_146067	---	-1.044	-1.507	2257	3051	3921	2510	2206	2446
NM_133804	Tmem132a	-1.044	-2.100	2659	2005	3734	1067	567	254
NM_126165	Vps4a	-1.040	-1.460	3247	2965	5122	462	411	342
NM_133847	Tm9sf4	-1.033	-1.200	5437	4258	4566	1193	853	1002
NM_025445	Arfgap3	-1.030	-2.228	2216	4888	6274	1678	1580	1101
NM_010096	Ebf3	-1.030	-1.141	5130	2312	6471	11280	3948	1600
NM_007986	Fap	-1.026	-1.705	170	1452	1572	2509	1478	3295
NM_024223	Crip2	-1.025	-1.240	3448	2426	5223	915	373	96
NM_175145	Tmem127	-1.025	-2.241	5388	5218	7912	326	398	281
NM_016796	Vamp4	-1.025	-2.134	3116	4434	3695	774	667	845
NM_010118	Egr2	-1.024	-1.838	2548	2740	4708	1998	386	362
NM_007752	Cp	-1.023	-1.328	102	825	818	1342	781	674
NM_008550	Man2b2	-1.012	-2.876	1542	2256	2406	1175	831	278
NM_019823	Cyp2d22	-1.001	-2.227	98	185	107	504	229	129
NM_025486	Hspc171	-1.001	-1.173	4764	4312	5277	50	139	49
