# Supplementary material for: Identification of the early and late responder genes during the generation of induced pluripotent stem cells from mouse fibroblasts
Source: PLoS One. 2017 Feb 2;12(2):e0171300. doi: 10.1371/journal.pone.0171300 (PMC5289558; doi:10.1371/journal.pone.0171300)
Supplement: S6 Table — (PDF) [file pone.0171300.s012.pdf]

S6 Table. Resistant down genes

Gene information		mRNA fold change		H3K4me3 enrichment			H3K27me3 enrichment		
RefSeq gene	gene symbol	iPSCp/sFB-G	mESC/sFB-G	mESC	iPSCp	sFB-G	mESC	iPSCp	sFB-G
NM_007742	Col1a1	-0.691	-7.483	825	8770	9809	2791	411	462
NM_011581	Thbs2	0.099	-7.126	495	11257	6291	3452	1194	731
NM_010656	Sspn	-0.362	-5.491	770	2724	2129	1437	876	967
NM_153543	Aldh1l2	0.173	-5.483	937	7691	3878	2636	1900	1397
NM_053110	Gpnmb	-0.585	-5.166	753	5061	5450	1357	1343	443
NM_011340	Serpinf1	-0.878	-5.136	234	3374	5430	839	334	380
NM_008608	Mmp14	-0.900	-4.904	1239	9694	9861	1154	234	251
NM_001081437	Fbln2	-0.057	-4.765	1178	8640	11480	4107	2214	1141
NM_007542	Bgn	-0.596	-4.705	115	3783	4039	801	448	117
NM_009829	Ccnd2	0.087	-4.698	1557	11400	9323	4872	2578	772
NM_015734	Col5a1	0.166	-4.655	3954	12259	14152	6686	2678	3330
NM_028266	Col16a1	0.374	-4.603	1648	3315	2456	7552	1430	1068
NM_029999	Lbh	0.965	-4.557	1725	18195	7558	2424	1096	608
NM_010284	Ghr	-0.510	-4.488	2490	8793	8403	7529	6521	6755
NM_011170	Prnp	-0.956	-4.374	1408	4700	3719	654	397	417
NM_010518	Igfbp5	0.564	-4.335	2987	1855	680	2709	1251	3608
NM_008304	Sdc2	-0.949	-4.285	993	2946	3285	2231	2193	3253
NM_007616	Cav1	0.762	-4.223	692	11524	6085	2534	915	962
NM_009932	Col4a2	0.002	-4.194	4959	8951	8288	8375	3550	2923
NM_010917	Nid1	-0.251	-4.189	347	756	1017	1152	1799	1189
NM_172753	Csgalnact1	-0.863	-4.140	4829	7789	9574	11626	7492	5408
NM_011923	Angptl2	-0.295	-4.131	1545	9684	11130	796	555	609
NM_028444	Prkcdbp	0.205	-4.117	849	4030	3092	556	39	20
NM_010128	Emp1	0.982	-3.858	237	12129	4322	1348	711	431
NM_053109	Clec2d	-0.602	-3.817	55	10285	3667	142	185	92

NM_018865	Wisp1	0.779	-3.812	371	9473	7575	1973	940	921
NM_177364	Sh3pxd2b	-0.486	-3.795	3629	6487	6747	4356	2191	2228
NM_033217	Ngfr	0.790	-3.793	3135	5605	1611	3547	2114	5178
NM_009465	Axl	1.122	-3.781	922	6201	3463	1701	731	495
NM_027976	Acsl5	-0.164	-3.729	764	3303	4402	1968	1271	1224
NM_023275	Rhoj	-0.155	-3.718	1294	5664	5289	4338	1524	1005
NM_027078	1700023M03Rik	-0.901	-3.689	3218	6331	5161	555	703	442
NM_021299	Ak3	-0.747	-3.663	2779	3937	4774	953	822	508
NM_008391	Irf2	-0.272	-3.661	4075	8201	6772	5064	2032	1333
NM_001048207	Gypc	-0.424	-3.628	1186	2716	3545	1971	996	749
NM_183308	Pon2	0.368	-3.624	944	3658	2497	1839	623	837
NM_011607	Tnc	1.502	-3.569	451	763	379	2186	1454	1041
NM_017464	Nedd9	-0.864	-3.569	1357	5159	5375	2949	1674	1440
NM_153138	Wipf1	-0.251	-3.563	3969	8252	10303	4537	1257	2024
NM_172589	Lhfpl2	-0.338	-3.552	4199	13058	11619	8587	3948	3513
NM_009755	Bmp1	-0.830	-3.552	2636	4663	8634	4854	1303	1083
NM_008332	Ifit2	-0.447	-3.539	358	3628	1824	1253	657	721
NM_172298	Tshz3	-0.713	-3.519	4067	3978	4407	4416	1863	770
NM_026582	Gpr177	0.013	-3.503	1460	7304	4436	3170	1863	1280
NM_029005	Mlkl	-0.232	-3.503	463	2643	3415	1713	624	492
NM_028713	Rftn2	-0.830	-3.497	762	4947	3969	2887	1408	1397
NM_146191	Lrrk1	0.399	-3.494	2582	5684	4392	7163	2057	1454
NM_018884	Pdzrn3	0.257	-3.484	3537	12973	10258	10483	7220	7149
NM_146090	Zadh2	-0.748	-3.426	3275	7000	7843	1682	284	122
NM_007778	Csf1	-0.925	-3.416	1503	8739	6579	2684	401	186
NM_009806	Cask	-0.469	-3.394	2281	5521	5255	4063	3996	4224
NM_009794	Capn2	0.338	-3.378	3150	7352	5768	3354	950	1117
NM_178660	Rbms3	-0.266	-3.376	3327	12774	13274	20828	12919	15914
NM_178711	Plscr4	-0.936	-3.354	620	3109	2847	1640	765	1092

NM_054040	Tulp4	-0.594	-3.316	657	1320	1143	2953	3330	2363
NM_007392	Acta2	0.799	-3.312	174	2270	852	697	785	260
NM_133685	Rab31	-0.623	-3.291	2793	7102	6560	4025	4039	2902
NM_008973	Ptn	0.837	-3.274	566	4859	972	3419	3267	5511
NM_172921	D930028F11Rik	-0.602	-3.254	1956	2813	3643	8589	5219	5154
NM_009812	Casp8	-0.359	-3.246	457	3495	3265	1561	1144	1227
NM_020606	Parva	-0.204	-3.240	3167	4499	3602	7497	3022	1795
NM_030612	Nfkbiz	0.444	-3.227	4506	16134	11366	1359	715	747
NM_009964	Cryab	-0.157	-3.217	333	3827	6506	580	40	110
NM_146258	Stard13	1.040	-3.183	1328	15412	7301	5516	3060	3611
NM_175088	Mdfic	-0.577	-3.158	2539	7630	7824	2502	2861	2338
NM_133662	Ier3	-0.453	-3.144	2095	4470	3953	146	147	41
NM_053201	Magee1	-0.862	-3.120	1524	2132	3744	382	15	39
NM_015760	Nox4	-0.083	-3.104	917	2842	1798	2983	1932	1828
NM_145509	5430435G22Rik	-0.481	-3.082	232	2046	3078	1099	767	594
NM_021890	Fads3	0.115	-3.075	1188	7140	5130	2200	613	431
NM_133990	Il13ra1	-0.785	-3.074	1059	2596	3587	1274	682	913
NM_175483	Snx33	-0.662	-3.051	1870	8675	11099	1993	273	358
NM_175386	Lhfp	0.361	-3.020	3121	7235	5672	7399	3929	2440
NM_025823	Pcyox1	-0.911	-3.009	4926	5590	4151	375	542	227
NM_009825	Serpinh1	-0.665	-2.996	1366	10032	7244	900	154	178
NM_008377	Lrig1	0.711	-2.991	5909	15264	9512	4067	3124	2033
NM_010685	Lamp2	-0.275	-2.985	1675	3194	2711	304	642	514
NM_009673	Anxa5	1.515	-2.982	2575	9197	3952	1595	741	204
NM_201518	Flrt2	0.693	-2.958	3249	11025	6114	4694	2054	1553
NM_008538	Marcks	0.015	-2.954	4853	11514	9545	619	159	154
NM_007585	Anxa2	0.392	-2.913	1432	8048	5925	2027	775	764
NM_025975	Dynlt3	-0.432	-2.900	504	1797	1869	120	207	109
NM_011182	Pscd3	-0.699	-2.895	2945	7382	8433	2439	1761	2058

NM_009369	Tgfbi	0.989	-2.881	1042	3929	2236	2589	981	972
NM_008737	Nrp1	0.424	-2.865	2406	6946	4397	5279	2759	2611
NM_019572	Hdac7	-0.807	-2.842	2960	5204	7940	4410	1804	966
NM_029100	Sepn1	0.021	-2.840	1990	2225	1441	690	636	169
NM_011520	Sdc3	0.195	-2.823	2276	4073	3078	3874	1499	325
NM_029682	Stambpl1	0.022	-2.815	1832	7794	6133	1888	1255	1380
NM_018763	Chst2	-0.593	-2.815	6216	3608	7025	2238	209	184
NM_153507	Cpne2	-0.932	-2.804	1875	3055	4855	3678	1157	1002
NM_011311	S100a4	-0.773	-2.791	20	1600	2020	290	36	16
NM_001040088	Sytl2	1.585	-2.790	142	136	373	685	549	503
NM_008135	Slc6a9	0.819	-2.786	6073	5588	3910	2115	933	378
NM_029777	Rhbdd1	0.001	-2.773	2949	4537	3981	3246	2942	3758
NM_146168	AW146242	-0.351	-2.770	3181	5054	5027	2394	2563	1773
NM_009242	Sparc	-0.759	-2.759	1708	14053	9320	923	903	492
NM_177388	Slc41a2	-0.049	-2.756	3885	4991	4486	5124	3908	3032
NM_026280	Mxra7	-0.896	-2.722	2603	6413	6634	1779	576	479
NM_133969	Cyp4v3	-0.633	-2.718	954	2428	1864	1290	647	351
NM_019989	Sh3bgrl	-0.519	-2.704	607	3207	2554	800	1113	931
NM_138309	Cd99l2	-0.807	-2.702	1082	1878	2211	1234	1118	920
NM_009154	Sema5a	0.093	-2.697	5643	10108	11268	11586	15837	20233
NM_001013025	Tgfbrap1	-0.646	-2.693	7399	7331	9292	1799	1233	1459
NM_010736	Ltbr	-0.431	-2.685	679	6976	7525	983	269	168
NM_146057	Dap	-0.656	-2.683	2646	5250	4753	2953	1629	1458
NM_007798	Ctsb	-0.392	-2.674	3412	6475	7259	448	658	656
NM_001081009	Parp8	-0.381	-2.673	3428	7520	8043	6382	3740	5164
NM_011787	Amfr	-0.305	-2.669	3774	4794	5578	1353	781	641
NM_026821	D4Bwg0951e	0.747	-2.653	3868	8743	4134	1340	642	939
NM_025424	Nenf	-0.965	-2.648	2329	3262	3112	625	387	239
NM_173182	Fndc3b	-0.101	-2.634	5493	8918	7144	6683	5752	3291

NM_008845	Pip4k2a	-0.759	-2.627	4985	5133	8831	5074	2535	3425
NM_008495	Lgals1	0.632	-2.622	804	8502	6099	213	177	160
NM_011701	Vim	0.965	-2.603	3451	19326	12302	619	218	215
NM_025664	Snx9	-0.635	-2.591	3758	8801	7168	2503	1795	1768
NM_001039530	Parp14	-0.603	-2.591	893	2662	3979	1177	763	932
NM_025558	Cyb5b	-0.359	-2.581	2488	3232	3425	549	594	741
NM_009396	Tnfaip2	0.708	-2.577	1175	6691	4387	4010	336	146
NM_011179	Psap	-0.691	-2.567	3904	4671	5697	634	722	518
NM_145406	Slc10a3	-0.823	-2.561	653	1308	1663	57	157	62
NM_019542	Nagk	-0.701	-2.560	3710	6803	5035	366	256	164
NM_153117	9530068E07Rik	-0.755	-2.553	3638	2739	3086	346	302	387
NM_009127	Scd1	-0.184	-2.548	1568	2908	2225	509	325	404
NM_010687	Large	-0.484	-2.541	5554	7840	10593	9597	8309	15388
NM_022563	Ddr2	-0.489	-2.523	825	781	1654	4361	2118	3908
NM_009207	Slc4a2	0.903	-2.523	2668	6050	4356	785	471	179
NM_008681	Ndrg1	0.200	-2.512	3019	7660	5972	2860	1905	1907
NM_008908	Ppic	-0.688	-2.511	2330	3116	4987	836	266	295
NM_001001491	Tpm4	0.373	-2.508	3974	5903	6790	535	236	234
NM_011824	Grem1	1.649	-2.505	2075	6171	4699	1974	320	1042
NM_010730	Anxa1	0.198	-2.504	131	9017	2944	624	409	842
NM_175150	Txndc15	-0.971	-2.504	2554	2926	2923	401	427	387
NM_020590	Gabarapl1	-0.630	-2.501	2902	6864	4652	306	400	206
NM_025277	Gng10	0.101	-2.500	1043	2539	2629	489	305	73
NM_177684	Zfp637	-0.345	-2.499	2864	4426	3645	144	179	65
NM_020332	Ank	0.172	-2.492	4153	7473	5008	4408	3614	3495
NM_012037	Vat1	-0.492	-2.490	5153	7203	7948	336	292	136
NM_207223	Acap3	-0.361	-2.489	5746	7930	7257	880	325	153
NM_173379	Leprel1	0.296	-2.484	2951	7667	4959	6542	3606	5363
NM_024437	Nudt7	0.373	-2.460	343	1506	2348	550	441	486

NM_011766	Zfpm2	-0.034	-2.442	3547	7278	9847	15201	11338	16140
NM_175535	Arhgap20	-0.920	-2.437	4155	2602	4984	3104	3126	2341
NM_010476	Hsd17b7	-0.772	-2.428	910	2931	2727	633	367	559
NM_019654	Socs5	-0.164	-2.419	4196	8061	7086	1295	1025	716
NM_011595	Timp3	-0.662	-2.415	2529	5132	4669	2237	5497	1798
NM_019791	Maged1	-0.671	-2.414	1753	2888	4433	198	152	71
NM_011578	Tgfbr3	-0.943	-2.412	5080	9214	7538	8403	4414	2108
NM_033144	Sept8	-0.858	-2.395	2609	5601	6414	1889	660	672
NM_013685	Tcf4	-0.492	-2.393	4745	17359	20060	8227	7957	9119
NM_011593	Timp1	-0.758	-2.384	849	2722	3097	54	55	35
NM_025473	1810037C20Rik	-0.803	-2.370	1391	2133	2387	164	267	125
NM_146119	Fam129b	0.054	-2.363	4942	9122	12676	1256	811	634
NM_007609	Casp4	-0.159	-2.348	40	2294	1740	533	442	654
NM_028493	Rhobtb3	0.344	-2.347	4263	5828	5590	2479	1414	1449
NM_009543	Rnf103	0.360	-2.339	4029	6288	5800	705	535	489
NM_173788	Npr2	-0.989	-2.336	3159	2083	2763	2085	1780	311
NM_175193	Golim4	0.367	-2.333	2966	6763	4933	2660	1812	1102
NM_148926	Zfand3	-0.092	-2.331	5123	7678	6164	3949	6527	4894
NM_010237	Frk	-0.975	-2.323	421	3061	5276	2944	2826	3910
NM_012056	Fkbp9	-0.779	-2.315	3152	5181	3288	773	1452	1007
NM_178111	Trp53inp2	-0.533	-2.310	2452	2497	3814	443	232	253
NM_144839	Ube2e2	-0.236	-2.304	3106	4479	5041	6854	6110	7339
NM_198160	Smarcc2	-0.670	-2.297	4189	6915	5000	1200	987	618
NM_009068	Ripk1	-0.853	-2.296	3195	3432	4178	981	953	1080
NM_001039387	Nelf	-0.914	-2.289	1345	2584	4628	977	382	175
NM_028643	Efha1	-0.195	-2.282	2304	3135	3142	2740	2007	2123
NM_134099	Fbxo4	-0.591	-2.265	1694	2052	2072	409	466	412
NM_007671	Cdkn2c	0.095	-2.265	3017	8818	4196	1569	64	53
NM_027992	2310036D22Rik	-0.773	-2.259	1943	4501	3306	273	373	455

NM_016753	Lxn	0.559	-2.259	1038	2543	2677	142	117	65
NM_024239	Stambp	0.042	-2.255	1053	3740	3533	940	688	533
NM_025772	Dtnbp1	-0.907	-2.249	3488	3939	5184	2469	1961	2139
NM_009368	Tgfb3	-0.263	-2.248	868	4184	3509	1607	359	331
NM_177740	Rgma	-0.662	-2.235	5805	9903	6358	8804	1281	1263
NM_028013	Endod1	1.020	-2.232	2444	3570	3310	1684	591	917
NM_175074	Hmgn3	0.492	-2.231	1207	2915	3888	1709	639	885
NM_181397	Rftn1	-0.830	-2.228	4057	4967	7027	8890	7037	5006
NM_010169	F2r	0.712	-2.220	3356	5425	4334	1269	661	593
NM_008862	Pkia	1.154	-2.218	1232	2432	1424	2104	1366	1190
NM_008882	Plxna2	-0.558	-2.214	4568	5236	9060	8386	4210	5757
NM_019813	Dbn1	-0.454	-2.207	1692	5343	6256	2330	673	372
NM_011777	Zyx	0.728	-2.202	3117	16179	9179	926	397	216
NM_009621	Adamts1	0.174	-2.191	2351	13917	10702	1550	396	253
NM_139272	Galnt2	-0.660	-2.190	5403	4993	10876	3193	2666	3452
NM_146142	Tdrd7	-0.135	-2.189	366	522	200	1899	1368	580
NM_010203	Fgf5	3.940	-2.189	1220	14664	231	2803	681	1029
NM_172814	Lrp12	0.151	-2.180	4781	6364	6232	1503	1694	1982
NM_018855	Gas8	-0.887	-2.180	1102	2438	5869	735	402	1102
NM_013470	Anxa3	-0.457	-2.166	597	3246	2319	2714	1219	775
NM_025745	4933407N01Rik	-0.976	-2.160	3935	3946	4532	651	508	760
NM_010875	Ncam1	0.682	-2.157	6059	7499	8744	9246	6887	8030
NM_018794	Atp6ap1	-0.308	-2.156	2752	2561	3122	89	50	141
NM_027399	Steap1	-0.017	-2.152	2191	3642	2531	675	410	140
NM_007899	Ecm1	-0.337	-2.146	493	405	591	430	218	35
NM_172845	Adamts4	0.845	-2.143	1155	3824	5117	526	249	98
NM_133626	Rrbp1	-0.182	-2.140	6349	4515	7588	504	402	423
NM_133748	Insig2	-0.518	-2.131	2766	4045	4412	598	561	664
NM_011627	Tpbg	-0.468	-2.122	4455	6723	8002	888	175	82

NM_009643	Ahnak	0.130	-2.115	5637	10174	11510	832	660	643
NM_009365	Tgfb1i1	0.541	-2.114	737	2121	2074	1062	156	97
NM_011078	Phf2	-0.432	-2.113	4792	4662	6691	2681	2169	1458
NM_025808	Lztr1	-0.593	-2.112	3496	4356	4493	369	656	344
NM_134142	Tmem109	-0.606	-2.112	1315	2324	3211	635	317	389
NM_172608	Tmem184b	-0.679	-2.108	4684	5221	8898	1526	1584	983
NM_139143	Slc39a6	0.490	-2.107	5092	5540	6083	728	769	604
NM_011673	Ugcg	0.148	-2.095	3614	12656	6480	713	739	322
NM_175195	3110056O03Rik	0.293	-2.091	4631	5995	5746	531	493	276
NM_011354	Serf2	-0.638	-2.087	4347	5443	5301	66	60	48
NM_021389	Sh3kbp1	1.569	-2.082	705	2211	1419	5281	3599	3245
NM_139297	Ugp2	-0.268	-2.082	2477	6449	4753	2045	1299	1398
NM_019880	Mtch1	-0.207	-2.080	3148	3329	4275	636	512	318
NM_007437	Aldh3a2	-0.248	-2.080	2347	3672	3528	1236	585	550
NM_007837	Ddit3	1.275	-2.073	4333	14807	5858	160	193	114
NM_009163	Sgpl1	-0.306	-2.071	7158	6815	8335	1163	1426	1611
NM_008783	Pbx1	-0.964	-2.056	2828	8476	10300	12601	7369	7325
NM_009128	Scd2	-0.303	-2.041	4801	13439	8710	259	572	264
NM_144832	BC017643	-0.949	-2.039	2173	2606	3650	226	167	213
NM_013826	Mocs2	0.039	-2.037	1981	3407	3168	377	316	256
NM_026995	Carkd	-0.516	-2.037	3681	3076	3230	307	379	375
NM_025932	Syap1	-0.200	-2.033	1361	1604	2044	270	507	310
NM_025831	1300014I06Rik	0.095	-2.033	3377	7771	6792	3597	798	646
NM_011546	Zeb1	0.227	-2.028	6315	7194	8330	3838	3571	4646
NM_019963	Stat2	-0.941	-2.028	2665	4567	3297	619	903	649
NM_008961	Pter	0.227	-2.026	912	2439	2971	1857	1188	1750
NM_029626	Glt8d1	-0.906	-2.026	4890	4070	5216	475	279	335
NM_023142	Arpc1b	-0.096	-2.018	3300	5476	5145	365	292	205
NM_008098	Mtpn	0.904	-2.014	4488	7358	5299	434	1025	798

NM_184052	Igf1	-0.942	-2.011	383	1922	2343	2387	1929	1780
NM_021607	Ncstn	-0.833	-2.010	4733	5734	5528	320	276	418
NM_053252	Ehbp1l1	0.578	-2.009	1452	5716	6634	1440	510	511
NM_007404	Adam9	-0.109	-2.005	4562	7072	4476	1298	1414	778
NM_153459	Dusp7	0.019	-2.002	3280	8415	8979	528	216	180
NM_028922	Ppapdc2	-0.684	-1.997	2711	2419	3453	415	188	134
NM_022993	Lrp10	-0.710	-1.995	3606	3248	3947	205	197	135
NM_007552	Bmi1	-0.481	-1.988	2054	2725	3074	3953	991	299
NM_011863	Papss1	1.326	-1.986	1730	3186	2033	1719	1821	746
NM_053147	Pcdhb22	-0.350	-1.984	147	123	113	920	114	96
NM_133987	Slc6a8	0.082	-1.979	2183	4983	4915	290	85	136
NM_027950	Osgin1	-0.330	-1.974	444	1601	3799	1205	457	453
NM_144822	Cbara1	-0.644	-1.973	3664	5746	5218	4448	3996	4097
NM_053197	Sfxn3	-0.300	-1.969	995	3621	4170	464	175	183
NM_016783	Pgrmc1	-0.955	-1.968	2857	2549	3044	82	105	128
NM_175514	Fam171b	0.755	-1.968	2581	2367	2486	2243	1158	1685
NM_199449	Zhx2	0.534	-1.959	3523	6256	6327	9364	5339	4154
NM_172413	Rap2c	-0.072	-1.958	1118	3010	2378	220	288	203
NM_018886	Lgals8	-0.480	-1.954	4110	4249	4287	865	478	414
NM_016686	Vezf1	-0.147	-1.951	4381	5189	4960	1767	404	375
NM_152808	Slc44a2	-0.531	-1.949	881	113	171	474	358	202
NM_146174	Fam115c	-0.804	-1.949	359	1377	1389	896	979	766
NM_011580	Thbs1	-0.318	-1.943	943	7596	8361	1393	317	337
NM_007783	Csk	-0.313	-1.930	2077	4610	5037	622	520	322
NM_134093	Letmd1	0.146	-1.929	1893	3296	4484	754	264	267
NM_153119	Plekho2	-0.140	-1.924	1630	6877	6860	789	566	434
NM_010789	Meis1	-0.520	-1.921	3333	10962	10201	12170	3082	3961
NM_030887	Jdp2	0.715	-1.919	3861	6326	3943	2849	1030	660
NM_019566	Rhog	0.143	-1.918	2379	4561	3839	564	292	103

NM_008512	Lrp1	-0.571	-1.911	5202	12925	10638	4806	3937	4564
NM_031998	Tsga14	-0.281	-1.910	867	2373	2179	1603	1391	988
NM_023168	Grina	-0.482	-1.906	2823	4979	5381	339	119	120
NM_021454	Cdc42ep5	-0.554	-1.905	635	2529	2434	411	240	145
NM_029646	2010004A03Rik	-0.616	-1.901	1121	2355	817	2533	1451	1240
NM_007387	Acp2	-0.427	-1.898	2079	3189	3013	195	116	304
NM_019924	Rps6ka4	0.112	-1.896	2201	3485	4884	3046	286	295
NM_172570	Trim47	-0.427	-1.895	2534	6343	6785	2639	222	87
NM_001012396	Ptpla	-0.468	-1.895	4645	3662	4368	1224	505	666
NM_133979	Ano10	-0.387	-1.894	2186	3409	3782	6912	3049	2756
NM_009254	Serpinb6a	-0.095	-1.890	2575	4882	4860	365	450	541
NM_024427	Tpm1	0.742	-1.887	8275	11573	11338	1562	699	899
NM_029793	Golga1	-0.625	-1.886	4407	4369	5209	840	728	1138
NM_026640	4632417K18Rik	0.747	-1.884	79	3278	1796	496	322	397
NM_145503	Lzts2	-0.219	-1.877	4453	9605	9853	1260	527	274
NM_010368	Gusb	-0.689	-1.876	1460	2626	3347	696	227	228
NM_025385	Prr13	-0.245	-1.875	766	2632	2914	85	134	77
NM_008012	Akr1b8	0.858	-1.873	158	2865	1224	495	295	333
NM_028162	Tbc1d5	0.027	-1.869	4190	8255	6914	9774	10295	11437
NM_029624	Lmf1	-0.339	-1.865	3337	4432	4208	3308	3598	2336
NM_023166	Mtvr2	-0.646	-1.861	2220	3797	5115	137	77	42
NM_021550	C1galt1c1	-0.928	-1.860	970	1453	1589	35	85	92
NM_030013	Cyp20a1	-0.313	-1.853	2804	2861	3084	984	898	1223
NM_008380	Inhba	1.107	-1.851	186	5931	6076	1671	280	580
NM_019760	Serinc1	-0.328	-1.849	3435	4833	4152	204	366	625
NM_147778	Commd3	-0.320	-1.847	2728	3526	4065	1399	102	69
NM_194342	Unc84b	0.345	-1.845	3708	5086	4162	1021	397	308
NM_026832	Cgrrf1	-0.395	-1.845	3240	3754	4527	1330	338	697
NM_009344	Phlda1	0.574	-1.842	6648	10972	8203	255	83	85

NM_010162	Ext1	-0.808	-1.842	7195	16292	16959	6997	9222	8029
NM_022028	Sav1	-0.163	-1.839	4077	4959	3854	631	571	359
NM_177263	Zhx3	-0.882	-1.837	2754	2779	4449	3865	2423	2342
NM_008794	Pcsk7	-0.654	-1.836	5626	5084	6197	421	375	517
NM_028662	Slc35b2	-0.365	-1.833	4120	4035	4807	146	200	72
NM_009821	Runx1	-0.129	-1.830	6533	15085	10504	8032	2670	3976
NM_207217	Itfg3	-0.733	-1.830	3394	3620	3212	1087	793	682
NM_011123	Plp1	1.724	-1.824	20	2995	116	229	465	203
NM_133895	Slc15a4	-0.843	-1.823	6037	5975	6289	513	495	415
NM_008058	Fzd8	-0.919	-1.823	7360	5099	8893	1091	150	41
NM_028258	2610524A10Rik	0.327	-1.822	1930	4787	4858	1241	587	1176
NM_178055	Dnajb2	-0.246	-1.822	2532	3295	3594	346	371	175
NM_011032	P4hb	-0.801	-1.819	7195	6174	7270	353	251	280
NM_008027	Flot1	-0.445	-1.815	2021	4257	4873	473	315	193
NM_013881	Ulk2	-0.224	-1.815	5150	4976	6140	3522	2062	2034
NM_146045	B4galt7	-0.183	-1.809	2489	2986	2912	280	332	225
NM_146047	Clptm1l	-0.727	-1.806	3841	3694	4677	557	218	566
NM_001012401	Hspb6	2.137	-1.803	1280	2364	1465	86	14	61
NM_027135	Sec24d	-0.848	-1.802	3218	5120	3994	2058	1987	1067
NM_021345	Ptplad1	-0.756	-1.802	4640	4048	5128	1064	597	740
NM_001025431	Btbd3	-0.709	-1.800	2591	5532	10872	530	558	590
NM_029394	Snx24	0.818	-1.797	3274	6062	4489	4269	3681	3564
NM_016807	Sdcbp	1.112	-1.794	1620	5150	3015	452	458	286
NM_178804	Slit2	0.788	-1.790	4975	12075	6797	8787	6332	6079
NM_020007	Mbnl1	1.098	-1.789	3367	11060	5674	2560	2246	1868
NM_027154	Tmbim1	-0.492	-1.788	2711	5338	5803	438	343	349
NM_052976	Ophn1	-0.821	-1.787	2735	3629	4381	4184	3798	3710
NM_028450	Gulp1	-0.283	-1.786	3839	6414	6841	6285	3919	6877
NM_024188	Oxct1	-0.071	-1.785	4775	5595	6435	2588	3289	3527

NM_010874	Nat2	-0.346	-1.783	43	1284	737	267	207	115
NM_133648	Slc12a6	-0.293	-1.777	866	1140	2006	1478	1114	1562
NM_019987	Ick	-0.611	-1.777	3476	2773	4103	1412	1223	1344
NM_178661	Creb3l2	0.432	-1.776	3948	11990	9080	3770	2881	2009
NM_001001326	St5	-0.305	-1.773	1311	1013	1157	2758	1808	1186
NM_008732	Slc11a2	-0.808	-1.773	3183	4111	5127	1196	1414	1295
NM_008684	Neo1	-0.777	-1.772	5203	4703	7134	3307	3278	4377
NM_019738	Nupr1	0.237	-1.766	1577	5143	3294	56	55	33
NM_146099	D19Wsu162e	-0.892	-1.759	3410	4367	7640	1711	1254	1076
NM_001005248	Hps5	-0.129	-1.758	3852	3818	3233	485	587	317
NM_009069	Rit1	0.229	-1.754	1355	4426	2379	249	178	132
NM_007670	Cdkn2b	0.656	-1.753	2288	3665	1799	842	140	85
NM_008083	Gap43	0.472	-1.748	839	2516	1002	3832	2595	5255
NM_016896	Map3k14	-0.907	-1.748	2672	3384	5537	3292	1084	1133
NM_146236	Tceal1	-0.686	-1.745	687	903	936	116	19	73
NM_153175	Gimap6	-0.980	-1.743	84	516	789	320	764	242
NM_011529	Tank	-0.123	-1.742	2733	4134	3592	1741	1481	2390
NM_007483	Rhob	0.688	-1.742	7353	8275	6591	406	96	38
NM_172865	Manea	1.323	-1.741	2662	4186	2469	404	413	288
NM_029745	Tbc1d9b	-0.597	-1.740	3602	3733	4085	1773	1003	802
NM_001042607	Ryk	-0.803	-1.740	4711	4738	6373	1300	1405	1772
NM_175686	Prrx1	0.138	-1.737	989	12279	11891	5586	1274	2855
NM_001048177	Jak2	-0.052	-1.736	2978	5835	5475	2096	1466	1489
NM_020042	Mocs1	-0.855	-1.732	1434	3444	3624	621	892	555
NM_177152	Lrig3	-0.749	-1.727	3355	10133	8658	1573	1213	1276
NM_009145	Nptn	-0.084	-1.726	3651	4119	4403	1102	1024	1490
NM_019422	Elovl1	0.351	-1.723	1294	4911	3422	107	39	63
NM_018769	Dfna5h	-0.774	-1.722	1235	2228	2509	2446	3529	1670
NM_011264	Rev3l	-0.682	-1.722	6319	5401	7114	2330	3153	4120

NM_144516	Zmynd11	-0.069	-1.716	5283	6768	7514	2288	1451	2171
NM_175383	B3gnt1	-0.496	-1.709	5286	5426	5618	471	151	57
NM_178908	BB146404	-0.893	-1.709	171	1010	1137	405	210	156
NM_146094	Fads1	0.114	-1.707	5370	6469	6510	444	433	470
NM_010163	Ext2	-0.850	-1.705	3386	3415	4292	3201	2538	2735
NM_007875	Dpagt1	-0.543	-1.703	2308	3230	4985	191	166	135
NM_010723	Lmo4	1.269	-1.697	3613	10137	5222	2678	336	205
NM_008688	Nfic	-0.062	-1.696	3297	6717	6893	2964	1192	598
NM_019749	Gabarap	-0.636	-1.691	2100	4550	4269	169	110	82
NM_011267	Rgs16	2.458	-1.689	1025	13659	2908	812	223	154
NM_008539	Smad1	-0.256	-1.688	4584	4199	6341	1675	1091	1276
NM_019564	Htra1	-0.249	-1.687	6675	5172	3239	2045	2713	1608
NM_134189	Galnt10	-0.543	-1.686	6097	5359	6616	8098	4100	3378
NM_025287	Spop	-0.138	-1.684	2002	4304	4097	2284	2073	2219
NM_025514	D10Ertd641e	-0.269	-1.683	3864	3280	3871	377	539	401
NM_011792	Bace1	-0.386	-1.683	2136	4526	6210	623	508	450
NM_173007	Tspan12	0.141	-1.681	2858	3038	4028	2974	3866	1540
NM_001039581	Abca3	0.020	-1.680	3917	4139	4770	2625	2297	1971
NM_010347	Aes	-0.462	-1.675	2614	6908	7774	252	294	161
NM_026524	Mid1ip1	0.548	-1.674	2920	5328	4787	71	68	22
NM_025861	Pqlc1	-0.710	-1.673	4561	4121	6380	2640	883	829
NM_001083315	St7	-0.094	-1.673	2014	3163	1908	5921	4777	5477
NM_024441	Hspb2	-0.298	-1.671	405	2122	5281	323	20	34
NM_025521	2310011J03Rik	-0.653	-1.670	2534	3390	3476	114	114	74
NM_010562	Ilk	0.738	-1.666	2750	7552	4066	173	239	48
NM_173451	Arsj	0.169	-1.666	401	4261	2434	2446	1678	1085
NM_028027	D10Ertd610e	-0.509	-1.663	2126	6904	5017	356	258	230
NM_001013770	AI747699	-0.671	-1.662	212	243	455	225	360	325
NM_172257	Sidt2	-0.765	-1.658	4316	4965	6591	754	386	310

NM_144551	Trib2	-0.325	-1.657	4136	7248	6380	1508	650	252
NM_173752	1110067D22Rik	-0.488	-1.652	4042	4053	4345	611	405	347
NM_146115	A830007P12Rik	-0.713	-1.651	2153	968	1717	251	135	90
NM_019581	Gtpbp2	1.267	-1.649	2031	6670	3265	242	430	274
NM_030084	Gpr108	-0.909	-1.647	3757	4273	3662	394	744	276
NM_130895	Adarb1	-0.472	-1.645	6088	4281	7010	5020	3897	3757
NM_029655	Snx7	0.995	-1.640	1114	5120	2298	2277	1383	1063
NM_172615	1700021K19Rik	0.757	-1.640	2453	4286	3590	1323	1241	1223
NM_025333	0610038F07Rik	-0.599	-1.635	3587	3902	4341	473	269	403
NM_021274	Cxcl10	-0.115	-1.632	49	447	60	343	98	58
NM_016906	Sec61a1	-0.529	-1.627	5579	8732	7573	257	610	322
NM_016978	Oat	-0.432	-1.626	1201	2583	1999	894	462	239
NM_183162	BC006779	-0.674	-1.626	658	1871	2747	1624	335	318
NM_024203	Fam120b	-0.142	-1.623	2952	4516	4481	1676	1662	983
NM_030561	BC004004	-0.616	-1.620	1855	3243	2857	886	842	842
NM_011873	Dazap2	-0.443	-1.620	4950	5337	6401	235	210	123
NM_011175	Lgmn	-0.953	-1.614	1849	3466	2594	940	697	508
NM_007522	Bad	-0.626	-1.613	6011	8072	9341	293	339	274
NM_001081260	Tnks1bp1	-0.403	-1.612	785	3943	6763	657	560	434
NM_133349	Zfand2a	0.260	-1.611	3855	4541	5281	337	532	296
NM_011890	Sgcb	0.818	-1.610	1176	2202	1779	1466	423	480
NM_026331	Slc25a37	0.001	-1.606	8257	8961	10307	2826	1536	1364
NM_175285	Tmem62	-0.471	-1.606	2821	2045	2994	956	598	613
NM_007581	Cacnb3	-0.830	-1.606	2129	2680	4339	872	523	247
NM_009279	Ssr4	-0.800	-1.604	5160	4296	4879	79	157	48
NM_016769	Smad3	-0.532	-1.601	4900	7827	11698	4301	2312	2009
NM_007400	Adam12	0.040	-1.598	3775	4204	5032	13095	7248	4289
NM_011711	Fmnl3	0.507	-1.597	1994	4712	3956	1922	1659	1577
NM_009295	Stxbp1	-0.235	-1.592	3335	3360	4370	2265	1139	1051

NM_026437	1810055E12Rik	-0.599	-1.588	2416	2536	2851	324	527	525
NM_212470	0610007C21Rik	-0.439	-1.585	4144	4618	3042	244	217	39
NM_170755	Fam134a	-0.701	-1.585	2923	4312	5184	546	178	108
NM_053267	Sepm	-0.944	-1.584	1718	2169	2853	518	163	71
NM_080837	D17Wsu104e	-0.198	-1.584	4174	4524	4089	273	210	181
NM_028005	2310047M10Rik	-0.600	-1.581	5122	5876	6868	213	36	45
NM_172525	Arhgap29	-0.741	-1.578	2879	4783	6431	2303	2139	831
NM_016719	Grb14	-0.376	-1.578	2801	3036	3572	4311	2433	3564
NM_029614	Prss23	-0.597	-1.574	1727	5134	3235	1454	309	127
NM_001007568	Zfp251	-0.308	-1.568	3966	4145	5841	725	335	384
NM_026775	Tmed10	-0.506	-1.565	2954	3141	1831	760	950	453
NM_011421	Smpd1	-0.606	-1.562	1184	2013	2325	436	75	61
NM_021428	Dexi	-0.822	-1.562	1640	3480	5099	894	278	345
NM_001003911	Adamts7	-0.505	-1.562	3912	3826	5847	3741	832	916
NM_001029983	Man1b1	-0.556	-1.560	5536	4086	5410	506	441	471
NM_145434	Nr1d1	-0.519	-1.559	1577	11110	8120	988	243	148
NM_028077	1810055G02Rik	-0.783	-1.557	3888	3315	4735	464	386	192
NM_010577	Itga5	0.619	-1.553	4172	7624	8216	612	593	584
NM_016794	Vamp8	0.255	-1.548	1317	4997	3974	241	117	122
NM_011609	Tnfrsf1a	-0.509	-1.548	1633	8410	10288	2120	355	258
NM_008349	Il10rb	-0.013	-1.548	1509	2902	2978	1704	552	594
NM_145410	Fam173a	0.483	-1.548	3787	3579	3953	106	121	63
NM_007881	Atn1	0.292	-1.548	5188	7244	4908	618	396	217
NM_013653	Ccl5	-0.592	-1.547	96	92	67	409	158	149
NM_146206	Tpcn2	0.075	-1.544	929	1724	1543	832	699	372
NM_025800	Ppp1r2	-0.391	-1.544	1696	4888	5245	633	677	716
NM_027905	1300018J18Rik	-0.588	-1.542	3069	4243	4895	373	464	289
NM_177583	Aph1b	-0.251	-1.541	3420	2208	2527	889	526	769
NM_172465	Zdhhc9	-0.625	-1.541	1959	2481	2890	723	325	393

NM_030245	Tada1l	-0.364	-1.538	2051	3479	3781	1598	248	335
NM_028186	Nkd2	2.648	-1.535	3074	4983	2588	2941	1305	1775
NM_001034168	Ank2	0.330	-1.533	70	209	114	751	328	244
NM_009557	Zfp46	0.576	-1.532	2358	3497	2738	1091	198	103
NM_134100	D15Mgi27	-0.457	-1.531	4003	3325	4737	121	72	49
NM_172488	9030625A04Rik	-0.383	-1.528	1875	1734	2951	576	340	368
NM_009177	St3gal1	-0.943	-1.527	2481	6365	11863	3955	2127	1736
NM_030256	Bcl9l	-0.931	-1.523	2169	1857	3776	725	446	198
NM_010227	Flna	0.842	-1.523	2523	6138	4292	342	404	237
NM_134149	AI837181	-0.880	-1.520	4187	3748	5227	99	101	85
NM_019765	Clip1	0.254	-1.520	5183	4953	3919	4674	2321	1012
NM_027409	Mospd1	0.078	-1.518	1551	2316	3191	285	190	301
NM_001001979	Megf10	2.688	-1.514	1670	3940	2099	6047	3770	9223
NM_021899	Foxj2	-0.078	-1.514	3211	5269	4572	1402	747	459
NM_016747	Dlg3	0.177	-1.513	2004	3355	3568	1049	1553	949
NM_001013028	AI597468	-0.524	-1.512	4251	8583	7331	371	329	466
NM_008364	Il1rap	-0.267	-1.511	1740	6592	6108	4597	2878	3960
NM_172398	2310005E10Rik	-0.141	-1.510	1378	3108	2617	300	455	255
NM_011273	Xpr1	-0.282	-1.508	3294	4289	5252	3582	3125	4012
NM_013509	Eno2	1.094	-1.507	2525	4606	3974	939	429	254
NM_023348	Snap29	-0.984	-1.501	4329	3844	4594	712	689	525
NM_198862	Nlgn2	-0.725	-1.495	3236	296	414	1945	448	358
NM_183171	Fez1	1.131	-1.493	252	257	255	1283	716	817
NM_017380	Sept9	0.675	-1.490	1758	1709	1610	1182	817	492
NM_009938	Copa	-0.644	-1.488	4908	6768	6812	908	893	939
NM_013630	Pkd1	-0.180	-1.487	4938	5619	6066	1761	1665	1045
NM_013863	Bag3	-0.131	-1.483	3573	5486	4808	1023	728	426
NM_172261	Ppp1r9b	-0.573	-1.481	4186	6430	8767	1724	549	354
NM_145516	Plekhb2	-0.573	-1.480	1310	1809	3016	1344	1096	779

NM_138315	Mical1	-0.466	-1.479	2394	4040	5097	1032	333	279
NM_001037878	Tcf25	-0.862	-1.477	4549	4070	7965	862	546	1279
NM_025982	Sas	0.034	-1.475	3473	6932	3869	74	141	131
NM_008749	Nucb1	-0.761	-1.475	1595	1958	2086	468	420	189
NM_029148	Txndc13	0.690	-1.474	1609	2671	2976	755	437	1109
NM_019914	Mllt11	1.084	-1.474	3915	7137	4377	161	383	84
NM_008853	Pja1	-0.157	-1.473	1236	1859	2313	143	97	65
NM_001081979	Mecp2	0.550	-1.473	2720	3041	2953	485	818	759
NM_026617	Tmbim4	-0.857	-1.470	3253	4305	3268	361	555	431
NM_020003	0610031J06Rik	-0.888	-1.470	3734	3129	2979	49	72	37
NM_198602	Cux1	0.284	-1.470	10315	9798	12048	9133	7586	7347
NM_013755	Gyg	-0.144	-1.469	2013	2561	2648	1710	800	421
NM_010360	Gstm5	-0.497	-1.468	527	2026	1272	539	30	25
NM_011026	P2rx4	-0.751	-1.468	3338	3689	2262	656	437	175
NM_174857	Mamdc2	-0.873	-1.464	1461	3156	3371	7800	6335	8891
NM_021292	Evc	-0.177	-1.463	2028	3350	3047	1926	1035	558
NM_029239	Prkd3	0.082	-1.462	571	791	635	1014	1475	1889
NM_007920	Elf1	-0.102	-1.462	5264	5995	7730	2500	2147	2350
NM_133749	2900064A13Rik	-0.604	-1.462	3991	3288	3963	285	84	221
NM_030750	Sgpp1	-0.727	-1.459	3945	6114	3951	1265	745	418
NM_008898	Por	0.099	-1.458	4511	5082	5511	1426	1128	699
NM_026418	Rgs10	-0.610	-1.457	3178	1774	2393	3365	1413	1332
NM_001001144	Scap	-0.607	-1.456	3743	2884	4141	1742	994	1159
NM_029862	5930434B04Rik	-0.962	-1.453	2177	2819	4294	363	282	233
NM_021493	4933428G20Rik	-0.904	-1.449	4963	2528	4695	3438	1342	1104
NM_172447	A330021E22Rik	-0.601	-1.448	2688	2918	1556	1964	1612	1007
NM_020050	Tmem9b	-0.160	-1.445	3880	4072	3677	732	347	183
NM_175134	Ankrd46	-0.297	-1.443	2695	2881	3211	498	648	608
NM_021518	Rab2a	0.653	-1.442	3252	5779	2957	863	1403	841

NM_008359	Il17ra	-0.622	-1.440	2578	4023	5579	1268	910	310
NM_145495	Rin1	1.331	-1.440	1822	6776	5216	560	232	186
NM_011893	Sh3bp2	1.613	-1.440	3287	5334	4311	2651	1095	383
NM_007897	Ebf1	-0.611	-1.440	4740	8849	14760	26777	11871	10676
NM_013758	Add3	-0.071	-1.440	2151	2946	3887	2622	2505	2682
NM_144794	Tmem63a	-0.240	-1.439	2490	3877	4376	1900	864	823
NM_178761	Zfp672	-0.329	-1.438	4168	2331	3333	1028	213	215
NM_198411	2610204M08Rik	1.139	-1.437	2604	5678	4463	2431	922	276
NM_013497	Creb3	-0.374	-1.434	2624	5570	3424	138	110	102
NM_008969	Ptgs1	-0.595	-1.432	603	2409	2758	1651	1247	552
NM_023063	Lima1	0.157	-1.431	728	12174	7628	826	1003	849
NM_026170	Ergic1	-0.363	-1.430	4614	6240	5993	2423	3742	2226
NM_026911	Spcs1	-0.361	-1.429	5477	4481	5345	201	102	49
NM_177648	Dolk	-0.592	-1.429	7494	4736	6758	96	154	52
NM_011925	Cd97	-0.588	-1.429	1492	3370	4047	440	366	365
NM_009105	Rsu1	-0.379	-1.428	2335	4431	4436	3994	2694	4191
NM_133187	1110032E23Rik	1.902	-1.427	532	10719	3016	1679	901	790
NM_009686	Apbb2	0.312	-1.427	8572	7472	5742	8259	6372	3381
NM_008549	Man2a1	-0.507	-1.426	4457	9238	8354	3660	4875	4309
NM_031373	Ogfr	-0.835	-1.425	3546	5114	5395	361	180	168
NM_008663	Myo7a	0.229	-1.422	1491	1908	2832	5105	1942	823
NM_001001566	Chpf	-0.994	-1.419	2917	2896	4316	453	326	861
NM_178760	Gpr107	-0.838	-1.419	3865	4795	5627	1592	1463	1323
NM_008980	Ptpra	-0.507	-1.418	3373	4017	5254	2723	1509	2296
NM_009610	Actg2	2.007	-1.415	245	489	264	1343	1661	1785
NM_173397	D930001I22Rik	-0.251	-1.415	3104	3239	3459	187	115	111
NM_026819	Dhrs1	-0.087	-1.415	2924	3085	3802	149	88	201
NM_020618	Smarce1	-0.382	-1.414	4190	5008	5926	597	362	618
NM_025911	Ccdc91	-0.196	-1.411	3193	5690	4636	3470	5005	3966

NM_016860	Actr1a	0.109	-1.409	3406	4385	4250	510	535	339
NM_001013380	Dync1li2	0.199	-1.409	4755	5050	6367	591	534	507
NM_008548	Man1a	-0.989	-1.407	4069	6348	9989	5319	3586	4761
NM_011866	Pde10a	0.012	-1.405	4882	6390	9091	9878	15998	5181
NM_016965	Nckap1	0.279	-1.404	3855	5453	5075	1210	1028	1804
NM_198113	Ssh3	-0.959	-1.403	1653	1379	3793	600	501	195
NM_028846	Usp20	-0.203	-1.399	508	281	341	1052	468	495
NM_020296	Rbms1	-0.105	-1.399	7142	13542	13934	6215	3782	4930
NM_145629	Pls3	-0.714	-1.398	1686	2787	3123	843	845	911
NM_207215	Mycbp2	-0.826	-1.398	3356	4353	9029	4113	4040	6318
NM_026669	Tmbim6	-0.110	-1.397	3179	7633	5761	345	564	441
NM_029924	Mbd5	-0.342	-1.397	4182	7049	8052	6516	4156	8003
NM_033475	Rab34	-0.665	-1.396	4245	4116	4742	198	175	509
NM_010345	Grb10	0.030	-1.396	4252	3192	3931	10065	5324	8377
NM_172605	Tdrd3	-0.325	-1.394	4040	4842	5689	2476	2241	3163
NM_028766	Tmem43	-0.072	-1.393	5386	7229	6564	610	759	310
NM_146012	Ctdsp2	-0.335	-1.393	89	62	58	67	80	75
NM_153088	Ctdsp1	-0.657	-1.387	3392	3677	5562	909	438	612
NM_023912	Scyl1	-0.611	-1.387	2909	4007	5157	465	259	212
NM_031494	Zfp275	-0.072	-1.386	2011	1947	2759	218	344	213
NM_011448	Sox9	-0.436	-1.384	3285	3446	6346	2545	324	391
NM_025576	2810004N20Rik	-0.345	-1.383	2574	4163	5174	213	63	197
NM_009073	Rom1	-0.972	-1.383	2344	1931	3354	161	204	90
NM_016920	Atp6v0a1	-0.364	-1.382	1933	2615	2164	2010	1505	1419
NM_022419	Abhd8	-0.815	-1.382	3363	3722	5758	1389	251	272
NM_181585	Pik3r3	0.449	-1.381	1954	4740	2329	1901	1504	911
NM_028521	Phospho2	-0.673	-1.376	1651	2717	2752	206	109	202
NM_009875	Cdkn1b	-0.147	-1.373	4912	13259	8916	455	151	76
NM_011050	Pdcd4	-0.946	-1.370	2941	5855	5423	1348	1298	1065

NM_013876	Rnf11	0.796	-1.370	3676	6738	3302	491	719	325
NM_026254	Tbc1d23	-0.956	-1.367	2564	3007	3635	1045	1107	1333
NM_008994	Pex2	1.133	-1.367	2444	4746	2920	600	294	223
NM_029561	Ndfip2	-0.006	-1.364	1745	2664	3401	1099	961	1182
NM_080553	Itpr3	0.267	-1.363	5467	6746	7386	2186	2112	1322
NM_027920	March8	-0.174	-1.362	2958	4329	3554	1804	2292	1749
NM_025730	Lrrk2	1.501	-1.360	969	7548	4369	5489	4005	4144
NM_019829	Stx5a	-0.560	-1.358	3034	3951	5402	333	289	319
NM_172557	Rufy1	-0.468	-1.357	3438	2576	2882	1595	1347	1128
NM_133710	Ctdspl	-0.358	-1.354	4313	3861	6794	6658	2278	2179
NM_029836	DXBwg1396e	-0.331	-1.352	1388	2330	2926	123	119	51
NM_133926	Camk1	0.088	-1.352	986	3464	3168	269	663	299
NM_133978	Cmtm7	-0.991	-1.351	3157	3773	5791	650	522	364
NM_198103	Exoc8	-0.472	-1.350	6350	6280	11030	269	176	211
NM_178772	Aadacl1	2.152	-1.347	2970	5218	3072	1725	1432	839
NM_013691	Thbs3	-0.804	-1.345	2957	1843	1893	522	438	181
NM_175266	Epm2aip1	-0.511	-1.340	5086	8095	8453	219	148	219
NM_019642	Rpn2	-0.598	-1.337	5113	4098	5229	775	896	755
NM_178592	Bat5	-0.902	-1.336	2033	2829	3005	363	606	364
NM_008138	Gnai2	0.018	-1.335	4795	8115	10736	807	524	245
NM_029019	Stard6	-0.266	-1.333	2775	2761	3440	476	385	495
NM_028173	Tram1	-0.995	-1.332	3203	3777	4975	619	572	614
NM_015807	Nt5c	-0.988	-1.329	2175	2368	3332	101	91	40
NM_022410	Myh9	0.152	-1.328	8284	13399	12904	2324	2698	1903
NM_022309	Cbfb	0.391	-1.327	4171	5785	5589	926	883	847
NM_145220	Appl2	-0.302	-1.326	3704	5876	4421	1169	1343	1096
NM_024189	Yaf2	0.295	-1.323	3066	4498	4002	2543	1433	1467
NM_029761	Dok5	-0.045	-1.323	1724	2104	4319	8528	2814	4110
NM_173392	Zfyve16	-0.198	-1.322	2518	4333	4519	1128	1091	1137

NM_029922	Parp6	-0.312	-1.315	1749	2993	3239	704	547	736
NM_025620	2210417D09Rik	-0.420	-1.312	43	51	23	94	162	112
NM_133771	Memo1	0.277	-1.311	5180	7068	6266	1685	2498	2549
NM_153538	Zcchc6	-0.286	-1.310	3753	3467	5332	1160	1394	1468
NM_133673	Tor1b	-0.829	-1.310	2641	2725	3490	174	124	128
NM_019955	Ripk3	-0.691	-1.309	945	1711	3323	868	744	87
NM_175300	Anapc2	-0.952	-1.306	5006	5517	7109	327	220	189
NM_178597	Camk2g	-0.148	-1.301	4842	4227	5661	1814	1735	1671
NM_016666	Aip	-0.997	-1.299	2638	3883	4089	337	440	320
NM_001047433	Dph3	-0.281	-1.299	2776	3054	2935	300	80	189
NM_011990	Slc7a11	2.527	-1.295	223	2204	706	2361	1642	1110
NM_053083	Loxl4	-0.814	-1.295	680	1348	2318	1586	808	407
NM_013870	Smtn	-0.445	-1.292	4432	4960	5778	1300	697	495
NM_173863	Crtc3	-0.022	-1.291	4081	4091	4530	4403	1563	1007
NM_138583	D16H22S680E	-0.806	-1.290	273	208	228	910	621	510
NM_175160	Zdhhc1	-0.565	-1.289	2847	3296	3644	2039	787	456
NM_028117	Chst14	-0.507	-1.289	2151	3460	4898	381	10	33
NM_146145	Jak1	-0.180	-1.284	4378	5849	4350	3366	2628	1662
NM_001024458	Add1	0.436	-1.283	3693	5633	3546	1250	1343	509
NM_001081161	Fam171a1	-0.416	-1.281	2881	3232	4687	4319	2068	2538
NM_009338	Acat2	-0.343	-1.281	4429	2536	3317	283	389	356
NM_025964	Fam119a	-0.639	-1.280	4275	5489	5391	597	96	406
NM_025646	Crls1	0.077	-1.279	2002	3247	3182	449	363	463
NM_175101	Tmem111	-0.162	-1.274	3406	5671	3769	276	692	432
NM_008854	Prkaca	-0.798	-1.272	3945	4272	8238	993	599	635
NM_001081156	2300002D11Rik	-0.148	-1.271	2724	2420	1500	1327	1654	432
NM_009045	Rela	-0.046	-1.271	3840	3840	4668	296	466	243
NM_026047	Rnf219	0.037	-1.270	1375	2605	3362	2058	1053	1239
NM_007916	Ddx19a	-0.018	-1.270	3998	3667	3606	316	326	341

NM_013757	Sytl4	-0.600	-1.270	478	660	2032	1221	1439	801
NM_011658	Twist1	0.079	-1.268	4280	6070	3863	866	335	282
NM_029432	4930402H24Rik	0.262	-1.268	5202	5783	6575	2847	2320	2655
NM_010140	Epha3	-0.743	-1.265	2659	3469	4295	9717	7442	16083
NM_011366	Sorbs3	-0.245	-1.264	1875	4949	8450	3249	549	493
NM_022331	Herpud1	-0.053	-1.262	6206	7755	7493	246	164	203
NM_025829	Eif4e3	1.007	-1.261	2005	4491	3528	3891	2535	1430
NM_171826	Cldnd1	0.074	-1.259	1640	2381	2424	164	153	221
NM_178066	1110012D08Rik	0.740	-1.257	4434	8283	5719	280	245	104
NM_176837	Arhgap18	-0.031	-1.256	1083	2541	2680	4704	3067	4008
NM_009357	Tex261	0.333	-1.253	3149	5452	4671	304	255	100
NM_033565	Aff4	-0.437	-1.250	4712	5140	5211	1744	1776	1774
NM_010753	Mxd4	-0.686	-1.250	3856	4591	5071	1262	487	67
NM_010329	Gp38	-0.537	-1.248	2993	4684	4892	682	1096	276
NM_019980	Litaf	-0.042	-1.245	3948	7598	6151	703	929	591
NM_153578	Nipa1	0.068	-1.245	3120	2711	2811	1724	790	702
NM_182994	Arl5a	0.484	-1.243	3138	8410	3833	464	584	460
NM_019390	Lmna	1.261	-1.241	1315	1980	879	1101	239	64
NM_134094	Ncald	1.136	-1.241	4699	8422	6002	19805	25301	15252
NM_175446	Zmat1	0.000	-1.240	839	1401	1255	1025	583	556
NM_145568	Krcc1	0.590	-1.240	3575	5735	3749	274	390	300
NM_133732	4931406C07Rik	-0.579	-1.239	103	160	201	429	295	376
NM_172772	B230380D07Rik	0.180	-1.236	4008	5441	5729	1636	929	1482
NM_020611	Srd5a3	0.279	-1.235	2386	3411	1778	413	347	148
NM_026760	2310036O22Rik	-0.515	-1.231	3188	3479	5164	109	100	166
NM_026139	Armcx2	0.499	-1.229	1009	2377	2558	153	50	48
NM_015817	Ppap2c	-0.390	-1.228	2376	2214	3542	417	290	268
NM_025976	Bfar	-0.916	-1.228	4346	3604	4205	516	594	716
NM_013471	Anxa4	0.093	-1.224	2657	5222	4404	2059	1715	1316

NM_021494	Dennd5a	1.034	-1.221	3209	5084	2686	1336	1310	711
NM_007498	Atf3	1.749	-1.220	4801	6138	4544	2547	323	816
NM_008013	Fgl2	-0.977	-1.219	339	986	1535	670	225	210
NM_053273	Ttyh2	-0.627	-1.218	4631	4850	6350	2002	1756	1269
NM_028000	Ppapdc1b	-0.273	-1.218	2850	2230	2150	124	229	104
NM_001004164	Gnptab	-0.472	-1.217	3205	5373	5039	2053	1394	1824
NM_008621	Mpp1	-0.762	-1.216	1324	2346	2662	205	316	313
NM_016903	Esd	-0.243	-1.215	2141	3349	3903	328	428	512
NM_011657	Tulp3	0.314	-1.215	2873	4993	3387	661	868	540
NM_011930	Clcn7	-0.499	-1.213	3261	4252	3820	808	875	757
NM_145984	D030028O16Rik	0.089	-1.212	4267	5289	4823	703	728	824
NM_010320	Gng8	0.275	-1.210	202	1122	454	673	146	122
NM_178644	Oaf	-0.402	-1.210	1826	6954	7865	2686	296	230
NM_053014	Agpat3	-0.235	-1.209	2933	3233	4011	4417	2160	1970
NM_012060	Bcap31	-0.709	-1.203	1932	2462	3457	247	504	303
NM_026172	Decr1	0.064	-1.203	1670	2421	1731	794	643	670
NM_011631	Hsp90b1	-0.156	-1.202	4770	8556	6418	152	511	318
NM_022018	Fam129a	0.560	-1.202	2788	4214	5447	4933	3679	4193
NM_025272	Atp6v0e	-0.575	-1.201	2684	4502	3664	721	1079	482
NM_010431	Hif1a	-0.679	-1.198	4549	6778	5479	760	875	676
NM_026416	S100a16	-0.305	-1.198	130	320	651	135	110	61
NM_019777	Ikbke	-0.670	-1.196	274	1546	4664	3254	1563	716
NM_030692	Sacm1l	-0.022	-1.194	3464	4424	4893	1130	1048	1481
NM_007737	Col5a2	0.141	-1.192	310	14315	7495	3307	2373	4192
NM_145496	Dak	-0.271	-1.192	1674	1464	1840	252	254	285
NM_134029	Nt5m	-0.035	-1.190	3090	3142	3903	1477	579	637
NM_178900	Prkd2	-0.518	-1.189	1518	3137	4016	1485	334	263
NM_027352	Gorasp2	-0.456	-1.187	4377	5636	6173	478	489	711
NM_001077638	Prmt2	0.077	-1.185	2076	3028	3546	1022	825	671

NM_144500	Osbpl2	-0.455	-1.182	3095	4210	5183	1159	710	983
NM_022432	Sirt2	0.461	-1.182	2253	3224	2415	715	422	210
NM_008917	Ppt1	0.296	-1.179	1771	2108	1575	354	556	382
NM_007569	Btg1	0.671	-1.177	4441	15795	7285	167	254	109
NM_007746	Map3k8	-0.605	-1.176	1746	3384	4941	1514	573	726
NM_001033172	Rab11fip2	-0.181	-1.175	2176	3792	4041	1219	1048	1397
NM_024454	Rab21	0.336	-1.175	4311	9289	5733	951	747	572
NM_133201	Mfn2	0.706	-1.175	3413	3605	2365	536	539	395
NM_028132	Pgm2	0.106	-1.174	3986	4628	3311	1652	1255	708
NM_027309	Lysmd2	-0.725	-1.173	4087	1939	3781	824	378	237
NM_133753	Errfi1	0.738	-1.171	1388	4827	2918	613	370	196
NM_181516	Taz	-0.267	-1.171	2066	2532	2886	107	194	72
NM_010434	Hipk3	-0.566	-1.169	3740	4659	6812	1524	1067	1281
NM_025674	Tcf19	1.295	-1.164	1111	4234	4292	155	230	115
NM_010176	Fah	-0.914	-1.163	1677	2099	1876	899	491	377
NM_019727	Snx1	-0.366	-1.158	2309	4204	4385	964	507	909
NM_001081241	2310066E14Rik	-0.375	-1.157	4901	6076	9284	1207	466	365
NM_028019	Rnf135	-0.707	-1.155	3523	3437	3355	525	411	462
NM_176785	Hps6	-0.339	-1.154	2861	3252	4152	191	109	72
NM_027219	Cdc42ep1	0.090	-1.152	1179	7626	6900	1382	235	168
NM_010073	Dpm2	-0.540	-1.152	2353	2721	3682	86	46	104
NM_021435	Slc35b4	0.062	-1.148	2764	4775	3585	547	630	474
NM_023908	Slco3a1	-0.814	-1.148	3658	3504	4697	10256	7895	3715
NM_025588	Exoc2	-0.110	-1.147	3730	3818	4555	3186	3677	4778
NM_021457	Fzd1	-0.918	-1.147	4817	4947	4747	1284	84	203
NM_027773	2310047D13Rik	0.032	-1.144	3346	2549	3063	1026	298	150
NM_029884	Hgsnat	0.283	-1.144	2344	3113	2147	733	514	265
NM_019657	Hsd17b12	-0.022	-1.143	4679	5906	4843	2222	2436	3443
NM_029657	Mgrn1	-0.606	-1.142	3864	3356	4984	1458	1348	1079

NM_029789	Lass2	0.819	-1.139	3962	4989	3842	186	125	95
NM_177757	Kif26b	-0.637	-1.139	840	357	639	4238	1836	1740
NM_008615	Mod1	-0.466	-1.138	3123	3264	4348	2764	1869	2430
NM_015828	Gne	-0.633	-1.138	5381	3911	3761	1992	1868	661
NM_001042534	Capg	0.469	-1.137	311	3360	3076	641	508	194
NM_019776	Snd1	-0.221	-1.136	10805	13588	9325	11580	11862	9780
NM_145920	Evc2	0.049	-1.134	1543	2890	2772	2692	1570	1005
NM_133656	Crk	0.283	-1.134	5259	4274	5079	729	753	592
NM_172843	Tor1aip2	-0.638	-1.134	179	215	67	305	280	304
NM_019713	Rassf1	0.719	-1.133	3080	7652	7219	360	187	190
NM_024206	Sec13	0.189	-1.132	3635	5976	4580	268	338	214
NM_008551	Mapkapk2	-0.009	-1.132	4507	5217	7658	2383	955	973
NM_172442	Dtx4	0.303	-1.132	4274	5153	4778	2578	1160	1156
NM_016963	Tmod3	-0.783	-1.131	4359	3929	5283	1232	957	1196
NM_013761	Srr	-0.395	-1.130	3991	4224	4154	522	641	574
NM_016740	S100a11	0.474	-1.129	1404	6254	2984	68	120	60
NM_026221	Ppfibp1	0.300	-1.128	3240	11521	8152	3701	3339	3256
NM_029956	Mmab	-0.012	-1.128	2548	2742	2284	396	300	217
NM_028015	Lass5	-0.353	-1.126	3330	4885	4350	947	903	976
NM_009658	Akr1b3	-0.726	-1.124	1941	4818	3635	508	477	219
NM_013770	Slc25a10	0.588	-1.124	4512	2703	3897	349	230	120
NM_181588	Cmbl	-0.224	-1.123	178	480	306	919	690	541
NM_145575	Cald1	0.285	-1.119	1841	9570	5653	2376	2031	1532
NM_019750	Nat6	-0.078	-1.118	1396	3133	3221	191	134	100
NM_173413	Rab8b	0.679	-1.118	2309	3622	3727	2446	1434	1713
NM_013785	Ihpk1	-0.273	-1.117	5622	5646	6707	719	772	689
NM_009506	Vegfc	1.062	-1.116	3106	4432	2992	1535	1699	1670
NM_025340	Sharpin	-0.296	-1.113	5787	6794	7410	122	170	141
NM_146234	Tmem32	0.302	-1.112	2714	1630	2406	148	92	154

NM_009758	Bmpr1a	-0.516	-1.112	4871	5981	6026	1741	1803	5053
NM_001012667	AI316807	0.397	-1.111	3451	3604	2435	470	338	147
NM_011868	Peci	-0.654	-1.109	1716	2385	2726	389	517	448
NM_019953	Cnpy2	-0.942	-1.108	3944	5866	4647	117	187	158
NM_010470	Hp1bp3	1.060	-1.108	7243	6759	4496	482	689	192
NM_144560	Gas2l1	-0.143	-1.108	4965	4208	5144	885	265	265
NM_025813	Mfsd1	-0.012	-1.107	2107	3505	2638	894	331	165
NM_016921	Tcirg1	-0.420	-1.106	780	2158	3001	509	208	181
NM_134135	Slc39a3	-0.220	-1.102	769	132	125	569	396	153
NM_026155	Ssr3	0.673	-1.102	2868	5271	3058	245	227	150
NM_009057	Rag1ap1	-0.463	-1.101	404	1576	1251	174	173	31
NM_010167	Eya4	-0.330	-1.099	4084	5598	9155	10222	6270	11563
NM_138741	Sdpr	1.342	-1.098	189	3823	1601	877	267	503
NM_001033633	Slc2a13	0.501	-1.097	5009	4788	5153	10220	10903	12371
NM_173431	Rpgrip1l	-0.351	-1.096	3937	3726	4995	2842	2293	2400
NM_144888	D430028G21Rik	-0.226	-1.095	1433	2686	3317	451	141	369
NM_001039180	Bicd2	-0.241	-1.095	5038	5429	8021	1290	1060	1162
NM_145599	Tmem184c	-0.091	-1.093	2698	2805	3128	260	362	414
NM_023409	Npc2	-0.633	-1.093	3829	6042	3448	419	607	295
NM_009427	Tob1	-0.680	-1.091	3775	3236	3787	234	119	137
NM_007460	Ap3d1	0.317	-1.091	4332	8222	6561	1170	1140	914
NM_031392	Wdr6	-0.746	-1.089	3369	2290	3690	266	244	185
NM_199200	Fam171a2	-0.687	-1.088	5688	2475	3685	2590	563	317
NM_153150	Slc25a1	-0.880	-1.087	3904	3777	5899	246	172	58
NM_024267	Ipo4	-0.425	-1.086	2086	2319	3378	475	242	167
NM_025442	Alg5	0.192	-1.086	5266	5171	4241	237	285	122
NM_009397	Tnfaip3	-0.452	-1.085	2598	3813	4378	1307	523	552
NM_009061	Rgs2	-0.642	-1.085	1046	1695	2216	1063	123	128
NM_175334	Maml1	-0.131	-1.082	5777	5612	5359	1293	681	854

NM_199448	Fez2	0.560	-1.078	2189	3502	2782	1293	1585	1090
NM_145392	Bag2	-0.101	-1.075	1523	2785	3459	1066	320	274
NM_138594	D6Wsu163e	0.193	-1.074	2927	4918	3538	723	804	702
NM_015742	Myo9b	0.465	-1.071	4061	4302	6246	2381	2325	2966
NM_024190	Chmp1b	-0.612	-1.071	3167	4230	4423	167	84	81
NM_007984	Fscn1	-0.741	-1.070	11287	12723	13289	969	410	350
NM_025931	Rabl4	-0.980	-1.070	2091	2549	3656	546	648	390
NM_199198	Hdac10	-0.232	-1.070	2596	3109	4974	400	178	116
NM_031256	Plekha3	0.176	-1.068	2252	3676	3184	419	435	421
NM_008062	G6pdx	-0.235	-1.065	3524	4376	3580	181	347	224
NM_007682	Cenpb	-0.001	-1.063	4499	4852	5955	146	130	44
NM_008761	Fxyd5	1.776	-1.063	215	1448	1249	1154	200	90
NM_153406	Specc1l	-0.154	-1.063	2830	3191	4077	1989	2644	2603
NM_019586	Ube2j1	-0.102	-1.062	3151	4830	2957	863	365	403
NM_007672	Cdr2	0.382	-1.062	3900	5974	4173	560	474	367
NM_001012517	Fut10	0.492	-1.061	1913	2745	2537	2463	1312	745
NM_133836	Il15ra	-0.824	-1.061	467	2823	4724	2225	888	544
NM_011864	Papss2	-0.322	-1.058	366	417	293	1545	1270	1229
NM_001033228	Itga1	0.598	-1.058	4498	8639	8564	6100	3953	4770
NM_172648	Ifi205	-0.849	-1.058	20	1697	1356	122	181	232
NM_024196	Tbc1d20	-0.803	-1.057	1859	3782	4022	385	281	361
NM_011639	Trip6	-0.325	-1.057	1526	4719	6933	182	141	84
NM_009640	Angpt1	-0.861	-1.055	821	2743	3531	7363	6706	10624
NM_019575	Scamp4	0.141	-1.052	5248	3681	3671	509	497	319
NM_001081170	Pacs2	0.192	-1.051	5441	4514	4052	2104	1732	962
NM_009367	Tgfb2	-0.424	-1.051	2635	7886	8004	4525	1792	2126
NM_021880	Prkar1a	0.117	-1.050	4186	6930	6754	520	435	399
NM_011961	Plod2	-0.442	-1.050	3477	4241	6431	1546	1264	1727
NM_016985	Mtmr1	-0.150	-1.050	1065	1700	2296	1146	369	738

NM_175489	Osbpl8	0.177	-1.049	3651	7123	5851	3522	3569	3233
NM_181344	C1rl	-0.728	-1.049	303	626	495	749	1313	309
NM_023041	Pex19	-0.614	-1.046	2417	3137	2833	277	278	218
NM_199146	AI451617	-0.748	-1.044	271	265	408	570	228	274
NM_026609	Leprotl1	0.918	-1.044	2574	6353	2586	340	326	98
NM_009504	Vdr	-0.959	-1.043	2336	2009	2920	4515	4364	2253
NM_016898	Cd164	-0.236	-1.043	5771	7208	7255	290	295	322
NM_009502	Vcl	0.373	-1.041	5357	7278	7504	2164	1682	2492
NM_010223	Fkbp8	-0.897	-1.036	2352	2117	3209	195	128	217
NM_017366	Acadvl	-0.892	-1.035	1346	1295	2265	233	141	100
NM_133977	Trf	0.181	-1.032	655	330	436	1691	739	799
NM_013759	Sepx1	-0.610	-1.032	1754	2445	3085	194	237	134
NM_025826	Acadsb	-0.880	-1.031	3828	3947	2962	1100	637	489
NM_008841	Pik3r2	-0.606	-1.030	5048	4457	5917	494	400	208
NM_016858	Rab33b	0.121	-1.027	4295	4930	3834	493	350	168
NM_145940	Wipi1	-0.249	-1.027	2899	3896	4841	1604	1212	702
NM_207670	Gripap1	0.269	-1.026	852	1149	1050	372	412	314
NM_011728	Xpa	0.180	-1.025	3803	3573	2997	1026	426	244
NM_011989	Slc27a4	-0.425	-1.022	2887	2788	3266	613	384	273
NM_145138	Nek9	0.237	-1.021	4302	4219	3273	958	1006	579
NM_031196	Slc19a1	-0.403	-1.020	3757	2977	3788	690	513	370
NM_178874	Tmcc2	0.283	-1.019	3059	3686	3724	2325	1598	900
NM_181278	B230219D22Rik	0.203	-1.018	3699	4066	3698	201	224	280
NM_023598	Arid5b	-0.055	-1.018	9407	14149	13544	5333	4807	4681
NM_001033420	Dock1	0.715	-1.014	8172	7191	7174	10765	9784	6191
NM_176987	4732471D19Rik	0.101	-1.014	2030	3182	4468	1276	1184	1004
NM_031185	Akap12	-0.765	-1.013	10111	6499	8787	1981	3472	2416
NM_028979	Cyp2j9	1.252	-1.013	192	1624	597	600	682	551
NM_007853	Degs1	-0.578	-1.013	3588	3798	4311	98	221	183

NM_152813	Plcd3	-0.207	-1.012	2850	4420	5615	2366	826	471
NM_010591	Jun	0.773	-1.012	10309	13230	7866	807	221	18
NM_207246	Rasgrp3	-0.967	-1.012	340	841	761	3005	4077	2406
NM_010453	Hoxa5	0.613	-1.007	2322	1318	2857	2212	643	124
NM_007893	E4f1	0.242	-1.007	2632	3187	3023	373	436	240
NM_008110	Gdf9	-0.518	-1.006	115	91	117	424	182	184
NM_178883	Scyl1bp1	-0.349	-1.006	1571	3581	4062	301	487	417
NM_199323	Tacc1	0.235	-1.003	4421	3048	3337	1271	804	493
NM_053207	Egln1	-0.356	-1.002	4745	6885	9186	1452	1047	1001
NM_016866	Stk39	0.625	-1.002	6038	4373	5638	12236	5891	7560
NM_025468	Sec11c	-0.089	-1.000	2595	3123	3470	883	385	302
