# Supplementary material for: Identification of the early and late responder genes during the generation of induced pluripotent stem cells from mouse fibroblasts
Source: PLoS One. 2017 Feb 2;12(2):e0171300. doi: 10.1371/journal.pone.0171300 (PMC5289558; doi:10.1371/journal.pone.0171300)
Supplement: S7 Table — (PDF) [file pone.0171300.s013.pdf]

S7 Table. KEGG pathways

Divergent up	Count	%	P-Value	Benjamini
Regulation of actin cytoskeleton	13	6.2	3.40E-05	3.50E-03
Focal adhesion	12	5.7	7.20E-05	3.70E-03
Melanoma	7	3.3	4.00E-04	1.30E-02
MAPK signaling pathway	12	5.7	9.10E-04	2.30E-02
Renal cell carcinoma	6	2.8	2.60E-03	5.30E-02
Prostate cancer	6	2.8	7.70E-03	1.20E-01
Glioma	5	2.4	1.10E-02	1.50E-01
Pathways in cancer	11	5.2	1.30E-02	1.50E-01
Pancreatic cancer	5	2.4	1.70E-02	1.80E-01
Bladder cancer	4	1.9	2.00E-02	1.80E-01

Convergent up	Count	%	P-Value	Benjamini
DNA replication	7	3	3.80E-06	3.30E-04
Spliceosome	10	4.2	2.10E-05	8.80E-04
Cell cycle	10	4.2	2.70E-05	7.60E-04
Oocyte meiosis	8	3.4	5.40E-04	1.10E-02
RNA degradation	6	2.5	8.60E-04	1.50E-02
Nucleotide excision repair	5	2.1	1.90E-03	2.70E-02
Mismatch repair	4	1.7	2.50E-03	2.90E-02
Progesterone-mediated oocyte maturation	6	2.5	4.10E-03	4.20E-02
Homologous recombination	4	1.7	4.50E-03	4.10E-02
Neurotrophin signaling pathway	6	2.5	2.30E-02	1.80E-01

Resistant up	Count	%	P-Value	Benjamini
DNA replication	11	1.1	1.30E-05	2.00E-03
Spliceosome	21	2.2	1.30E-05	1.00E-03
Pyrimidine metabolism	16	1.6	2.30E-04	1.20E-02
Nucleotide excision repair	10	1	4.80E-04	1.80E-02
Ribosome	14	1.4	1.20E-03	3.50E-02
Basal transcription factors	8	0.8	1.90E-03	4.70E-02
Purine metabolism	19	1.9	2.60E-03	5.50E-02
Base excision repair	8	0.8	5.90E-03	1.10E-01
Mismatch repair	6	0.6	6.20E-03	1.00E-01
Selenoamino acid metabolism	6	0.6	7.60E-03	1.10E-01

Divergent down	Count	%	P-Value	Benjamini
Leukocyte transendothelial migration	6	5.3	3.50E-03	2.70E-01
Alanine, aspartate and glutamate metabolism	3	2.7	2.80E-02	7.10E-01

Convergent down	Count	%	P-Value	Benjamini
Lysosome	16	3.9	1.70E-07	2.30E-05
Metabolism of xenobiotics by cytochrome P450	8	2	1.10E-03	7.00E-02
Glycosaminoglycan degradation	5	1.2	2.20E-03	9.10E-02
Focal adhesion	13	3.2	3.50E-03	1.10E-01
ECM-receptor interaction	8	2	4.20E-03	1.00E-01
Other glycan degradation	4	1	6.60E-03	1.30E-01
Cytokine-cytokine receptor interaction	14	3.4	7.10E-03	1.20E-01
Glutathione metabolism	6	1.5	8.80E-03	1.30E-01
Drug metabolism	7	1.7	1.00E-02	1.40E-01
Toll-like receptor signaling pathway	8	2	1.10E-02	1.30E-01

Resistant down	Count	%	P-Value	Benjamini
Focal adhesion	26	3.4	2.40E-07	3.60E-05
Lysosome	16	2.1	7.00E-05	5.20E-03
Apoptosis	13	1.7	1.60E-04	7.80E-03
ECM-receptor interaction	12	1.5	4.30E-04	1.60E-02
TGF-beta signaling pathway	11	1.4	2.30E-03	6.70E-02
Chronic myeloid leukemia	10	1.3	3.10E-03	7.40E-02
Dilated cardiomyopathy	11	1.4	3.50E-03	7.20E-02
Hypertrophic cardiomyopathy (HCM)	10	1.3	6.10E-03	1.10E-01
Pancreatic cancer	9	1.2	7.70E-03	1.20E-01
N-Glycan biosynthesis	7	0.9	9.50E-03	1.30E-01
